# Supplementary material for: Phosphorylation regulates viral biomolecular condensates to promote infectious progeny production
Source: EMBO J. 2024 Jan 2;43(2):6. doi: 10.1038/s44318-023-00021-0 (PMC10897327; doi:10.1038/s44318-023-00021-0)
Supplement: Supplementary file 2 — Movie EV1 [file 44318_2023_21_MOESM2_ESM.zip › Movie EV1/Movie EV1_Legend.docx]

**Movie EV1 – FRAP of punctate WT 52K-GFP nuclear bodies in transfected HEK293.**

**A.** Fluorescence recovery after photobleaching of a punctate nuclear body formed by expressing WT 52K-GFP via transfection of HEK293 cells for 24 hours. Diameter of punctate nuclear body < 1μM. Movie frame rate = 4 frames per second. Movie length = 100 frames, 25 seconds.
