# Supplementary material for: Phosphorylation regulates viral biomolecular condensates to promote infectious progeny production
Source: EMBO J. 2024 Jan 2;43(2):6. doi: 10.1038/s44318-023-00021-0 (PMC10897327; doi:10.1038/s44318-023-00021-0)
Supplement: Supplementary file 5 — Movie EV4 [file 44318_2023_21_MOESM5_ESM.zip › Movie EV4/Movie EV4_Legend.docx]

**Movie EV4 – FRAP of large WT 52K-GFP nuclear bodies in transfected HEK293.**

**A.** Fluorescence recovery after photobleaching within a large nuclear body formed by expressing WT 52K-GFP via transfection of HEK293 cells for 24 hours. Internal bleach spot diameter = 4.155 μm. Movie frame rate = 4 frames per second. Movie length = 100 frames, 25 seconds.
