# Supplementary figures and images for: Phosphorylation regulates viral biomolecular condensates to promote infectious progeny production
Source: EMBO J. 2024 Jan 2;43(2):6. doi: 10.1038/s44318-023-00021-0 (PMC10897327; doi:10.1038/s44318-023-00021-0)

Figure 1: Panel A

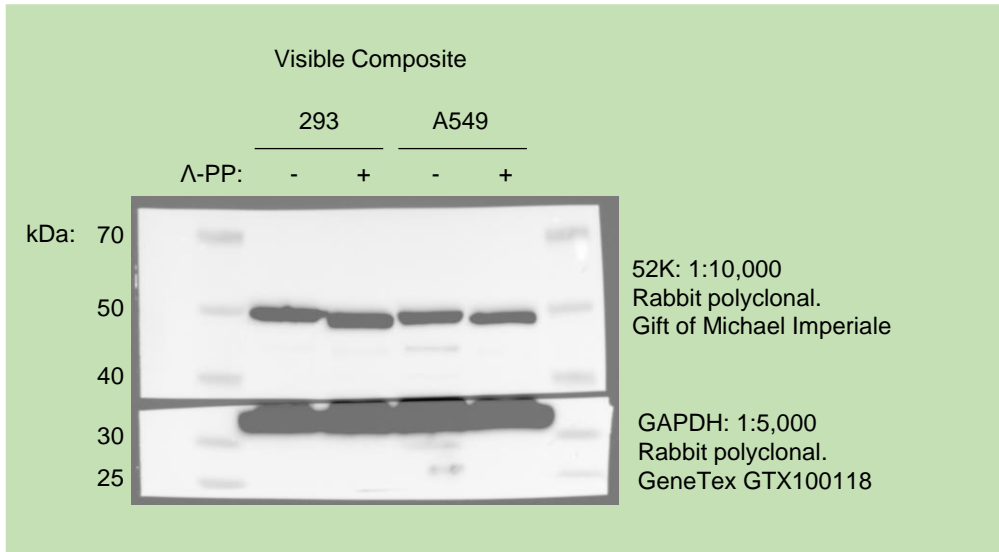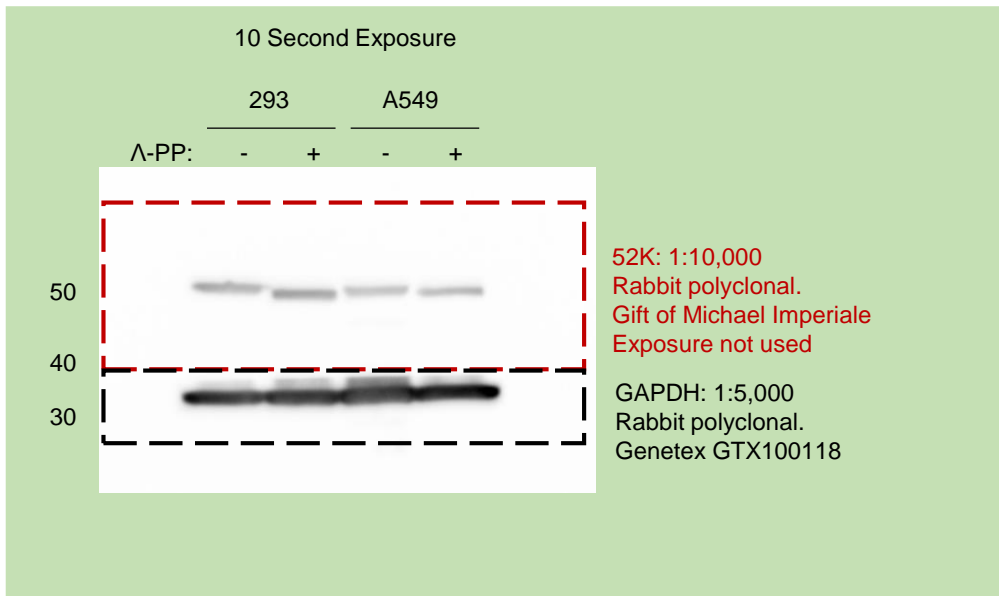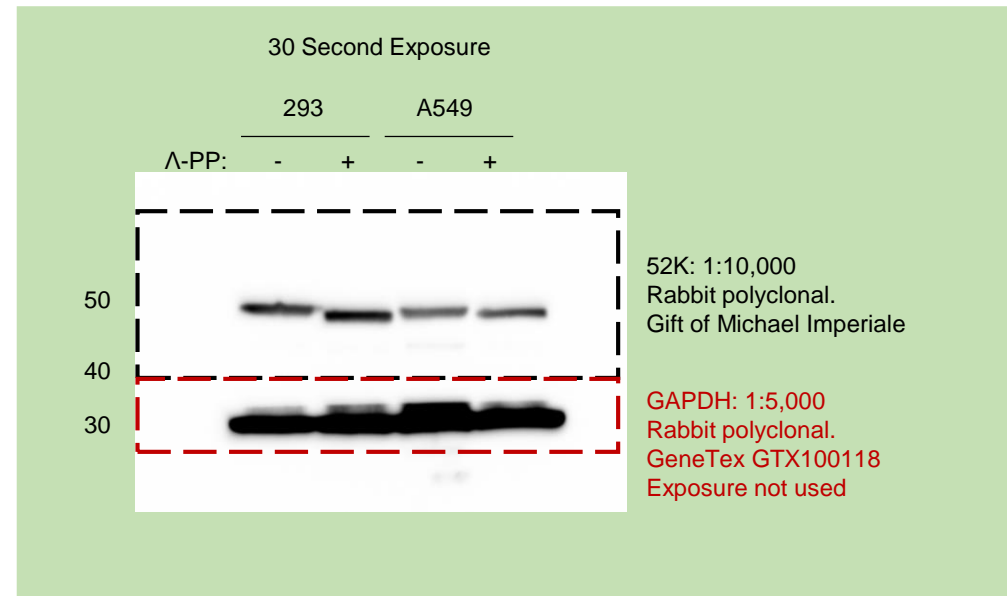

Supplement: Supplementary file 8 — Source Data Fig. 1 [file 44318_2023_21_MOESM8_ESM.zip › Figure 1/Figure 1A/Panel A_Lambda Protein Phosphatase Immunblot.pdf]

Figure 1: Panel G

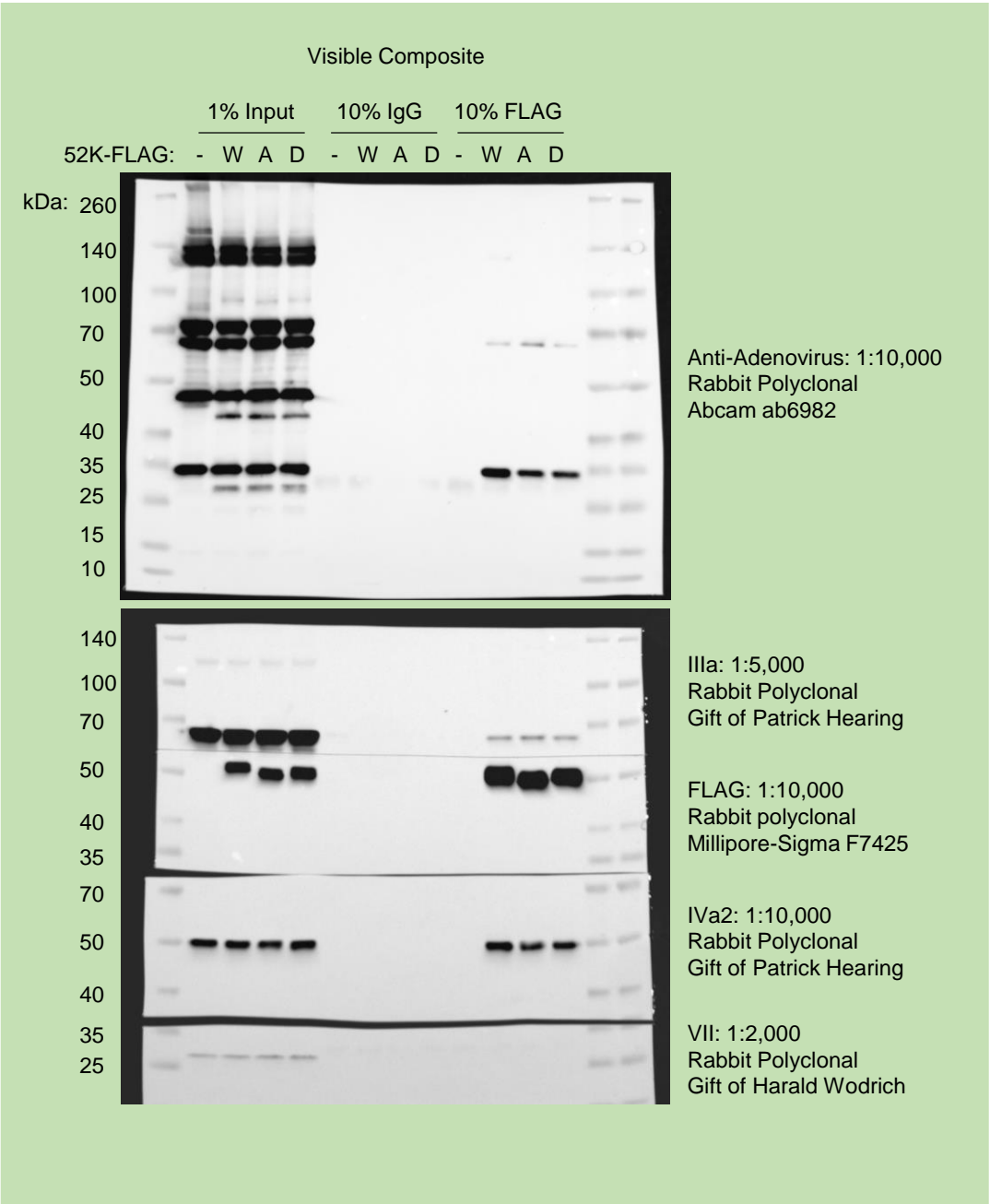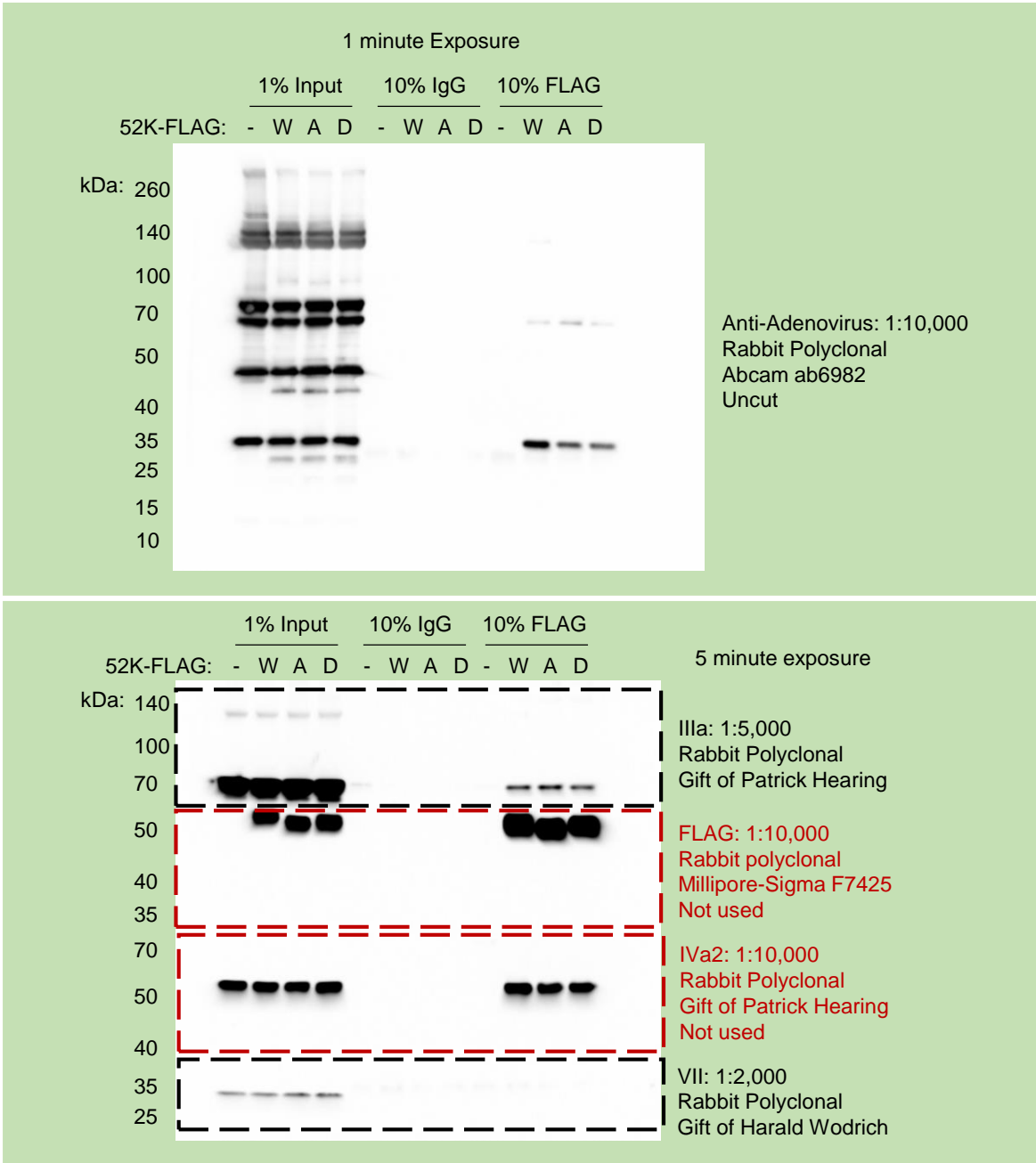

Figure 1: Panel G continued

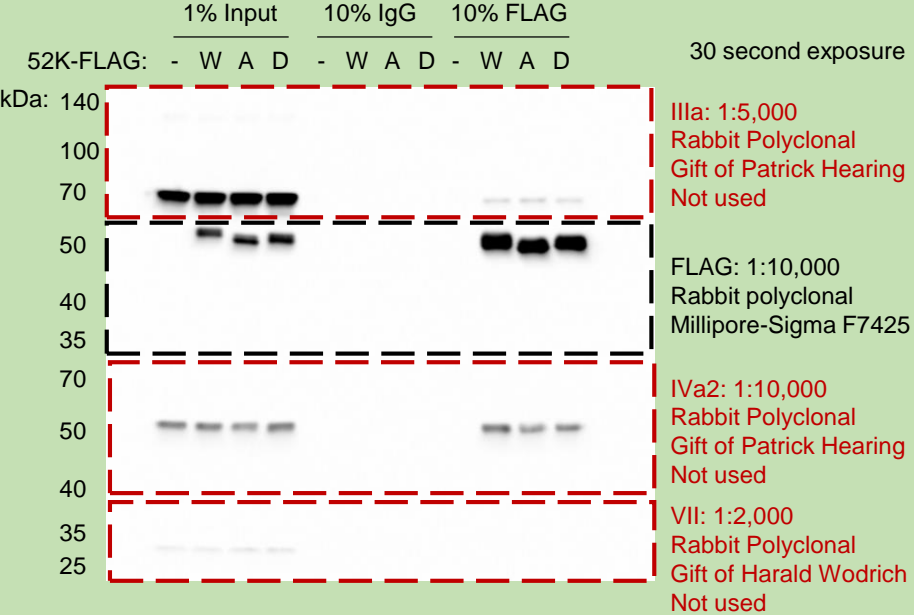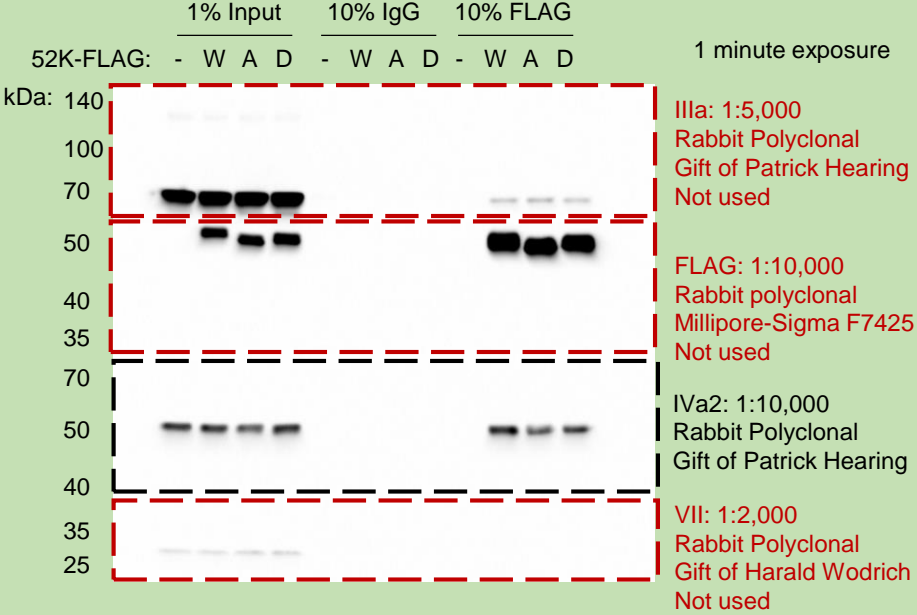

Supplement: Supplementary file 8 — Source Data Fig. 1 [file 44318_2023_21_MOESM8_ESM.zip › Figure 1/Figure 1G/Panel F_52K Co-IP-Immunoblot.pdf]

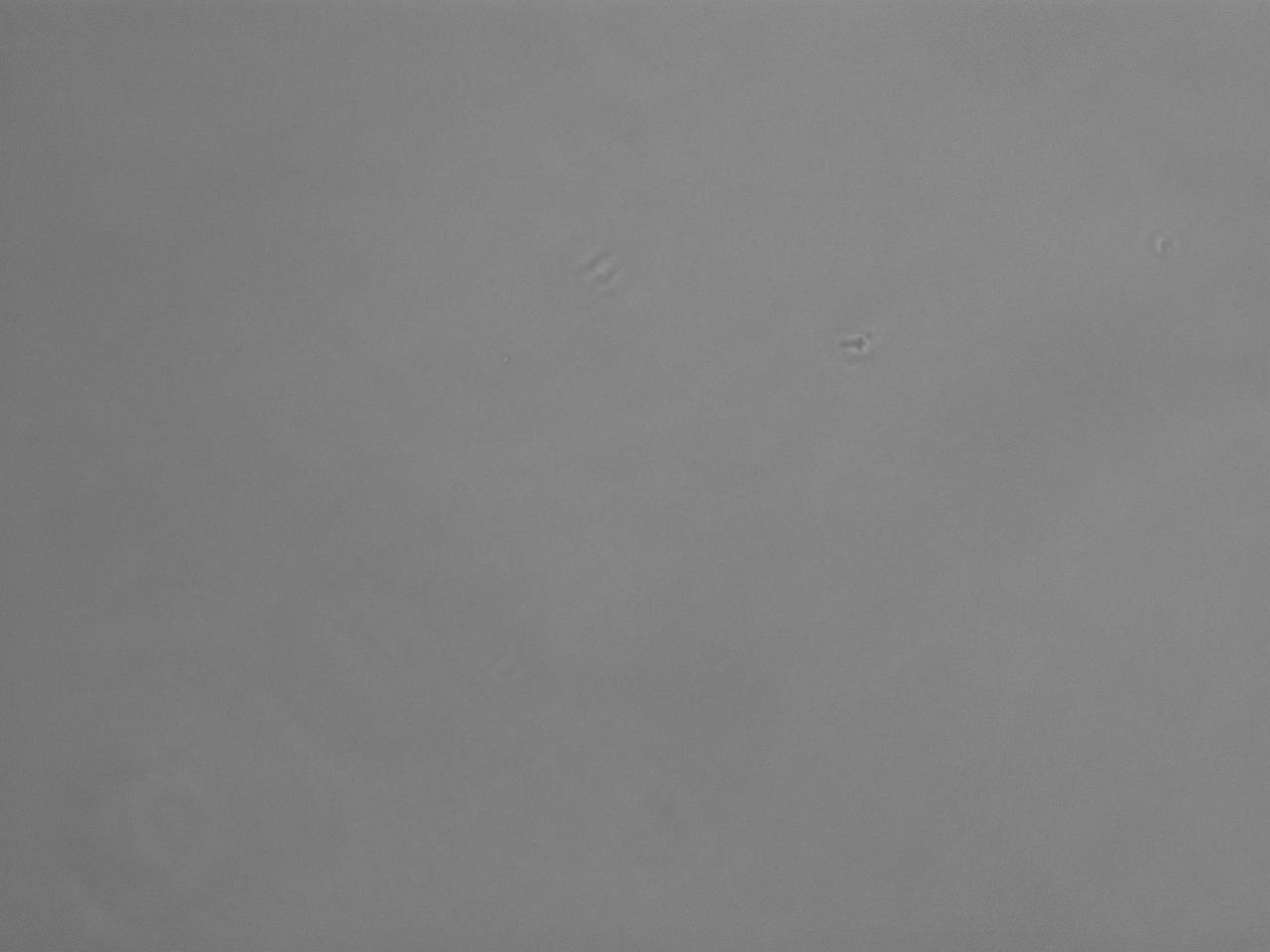

Supplement: Supplementary file 9 — Source Data Fig. 2 [file 44318_2023_21_MOESM9_ESM.zip › Figure 2/Figure 2A/MBP-52K S_A 0.3125 uM.tif]

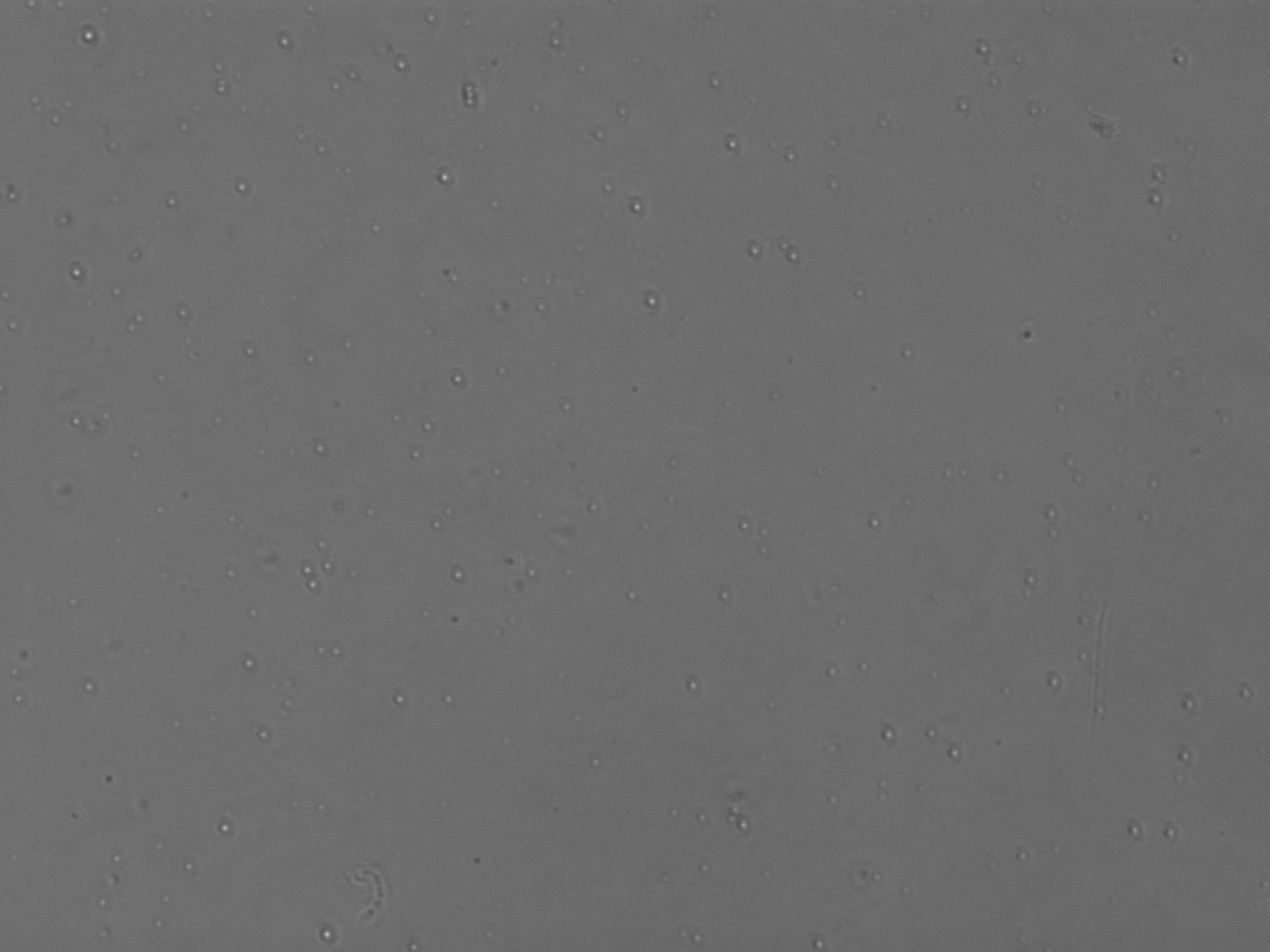

Supplement: Supplementary file 9 — Source Data Fig. 2 [file 44318_2023_21_MOESM9_ESM.zip › Figure 2/Figure 2A/MBP-52K S_A 0.625 uM.tif]

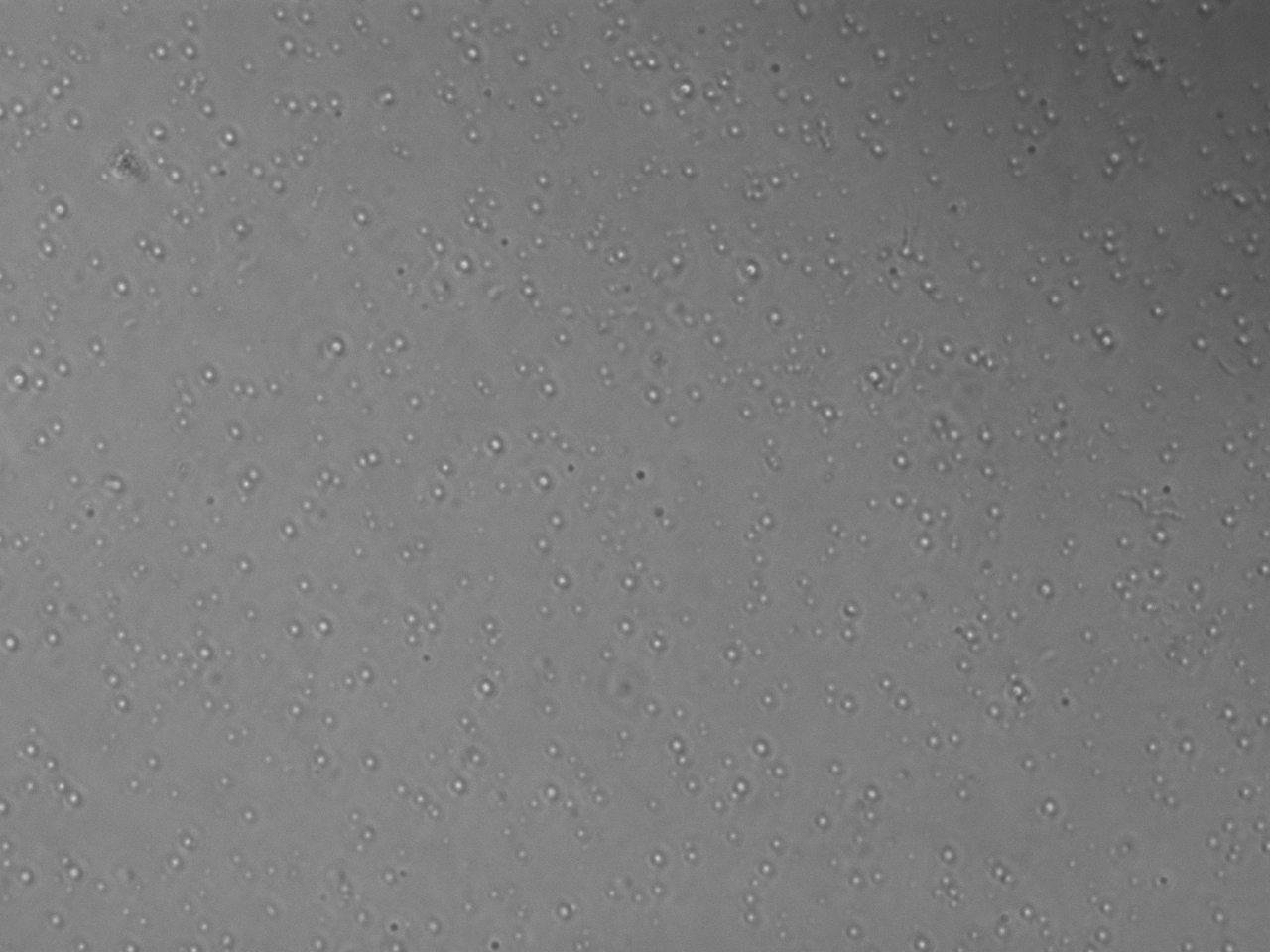

Supplement: Supplementary file 9 — Source Data Fig. 2 [file 44318_2023_21_MOESM9_ESM.zip › Figure 2/Figure 2A/MBP-52K S_A 1.25 uM.tif]

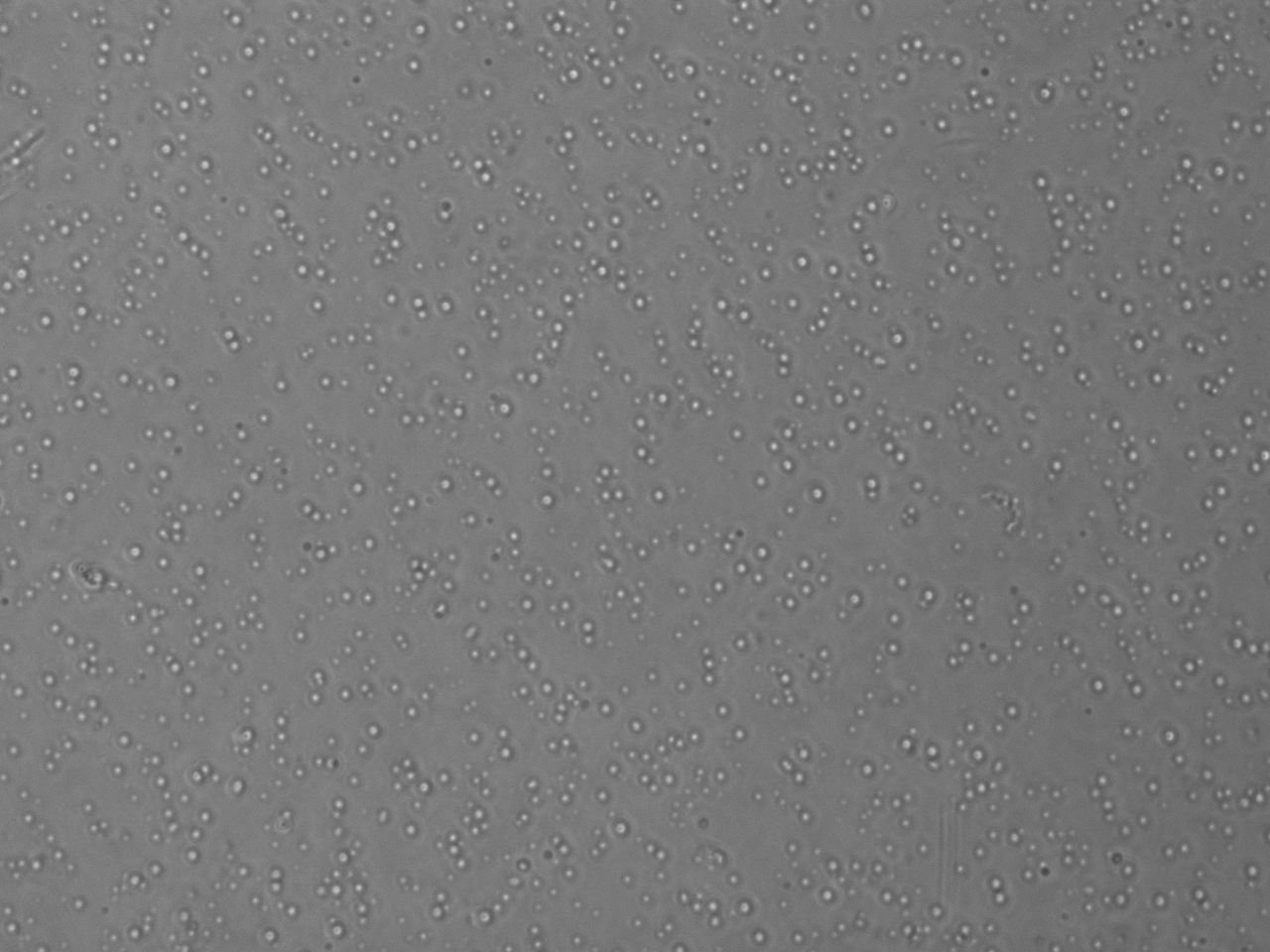

Supplement: Supplementary file 9 — Source Data Fig. 2 [file 44318_2023_21_MOESM9_ESM.zip › Figure 2/Figure 2A/MBP-52K S_A 2.5 uM.tif]

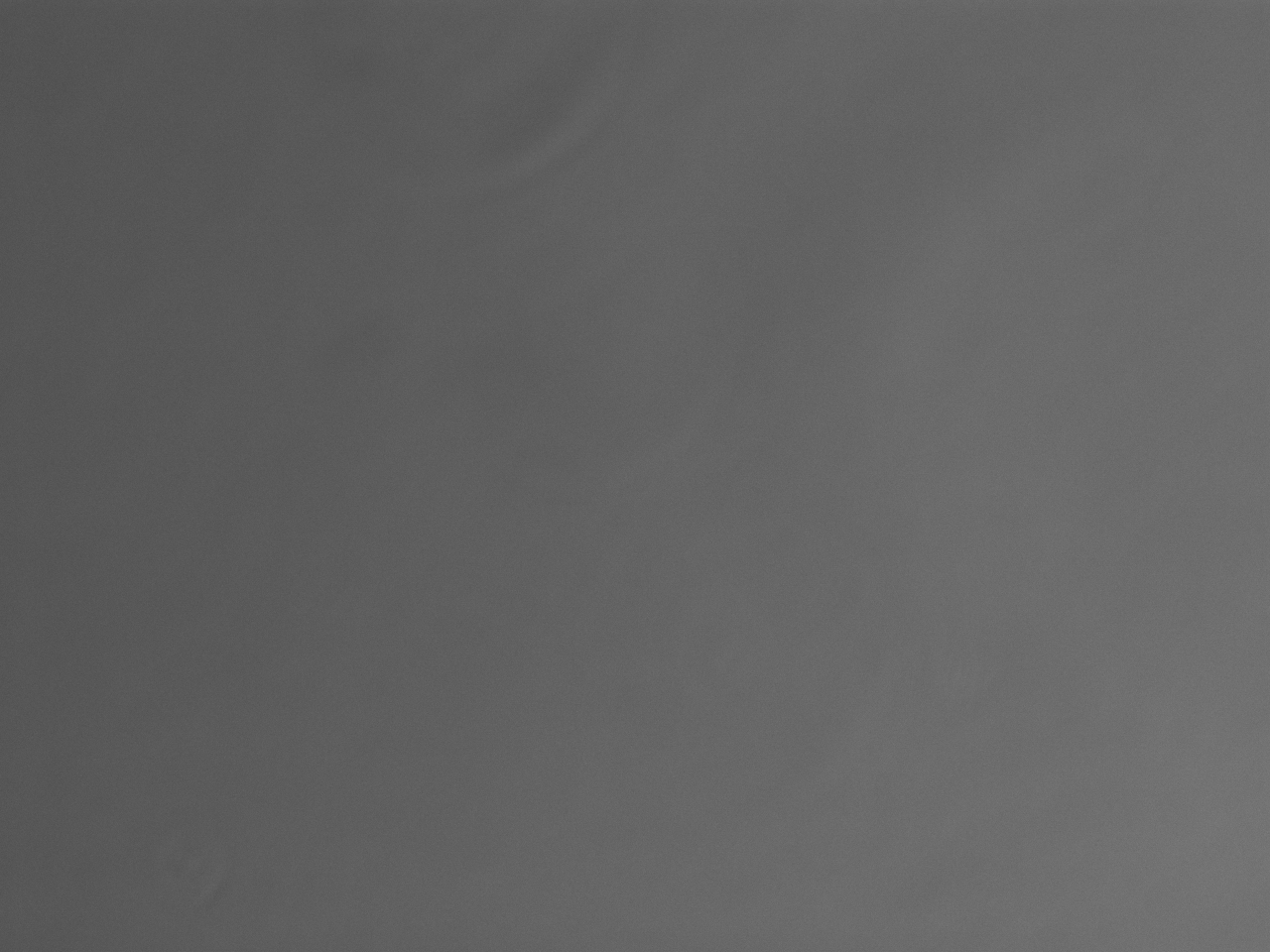

Supplement: Supplementary file 9 — Source Data Fig. 2 [file 44318_2023_21_MOESM9_ESM.zip › Figure 2/Figure 2A/MBP-52K S_A 2.5 uM_- TEV CTL.tif]

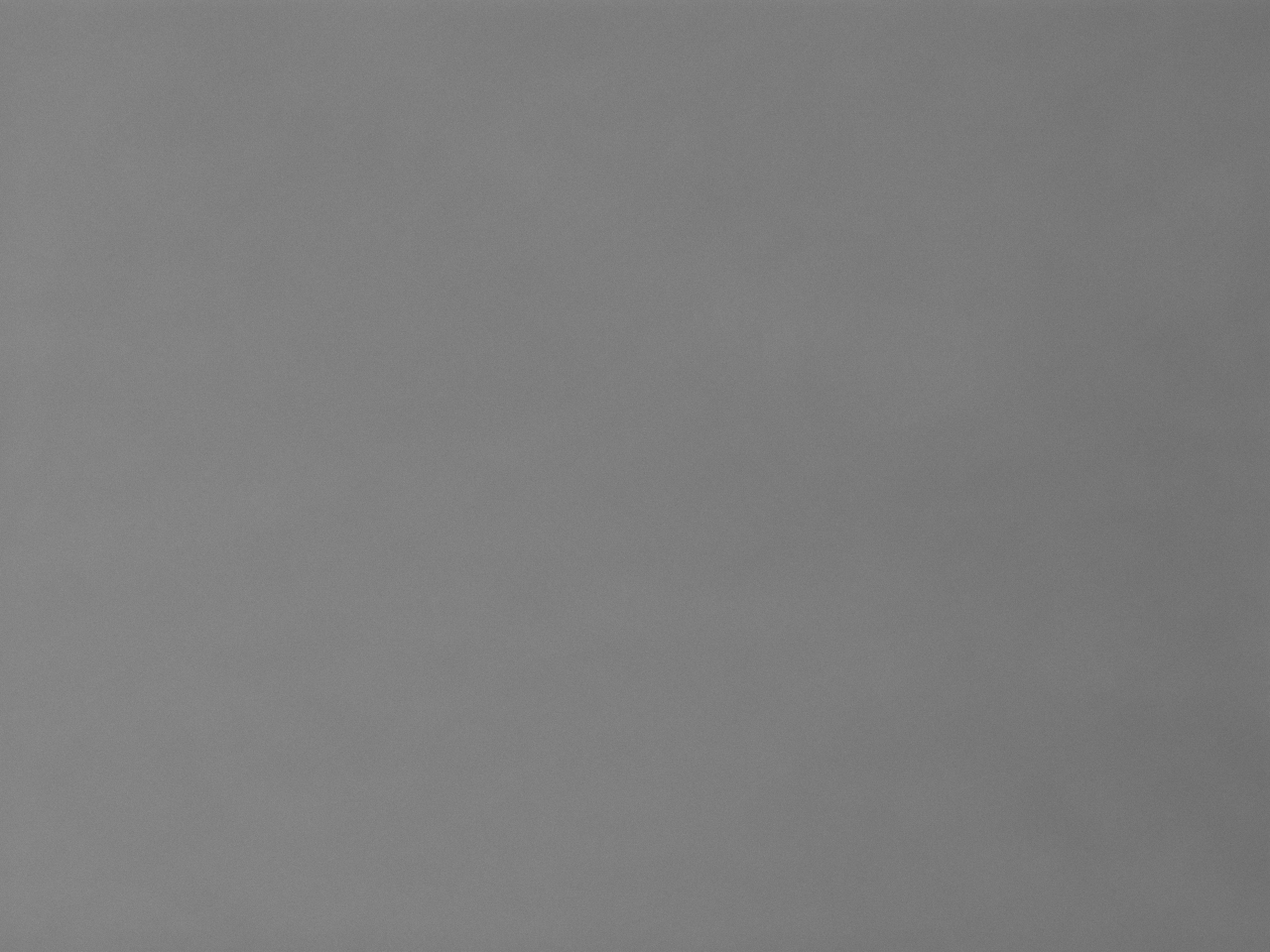

Supplement: Supplementary file 9 — Source Data Fig. 2 [file 44318_2023_21_MOESM9_ESM.zip › Figure 2/Figure 2A/MBP-52K S_D 0.3125 uM.tif]

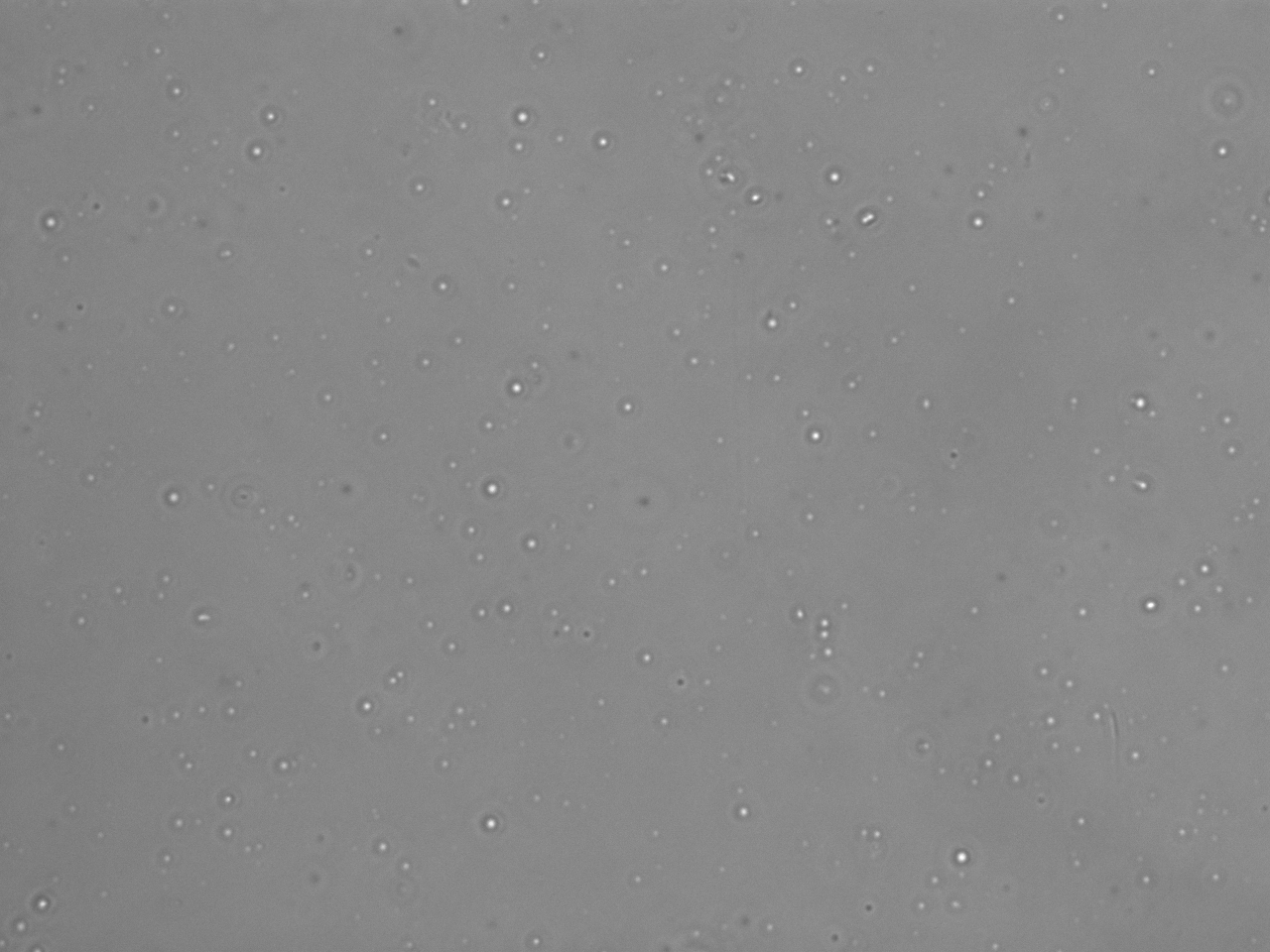

Supplement: Supplementary file 9 — Source Data Fig. 2 [file 44318_2023_21_MOESM9_ESM.zip › Figure 2/Figure 2A/MBP-52K S_D 0.625 uM.tif]

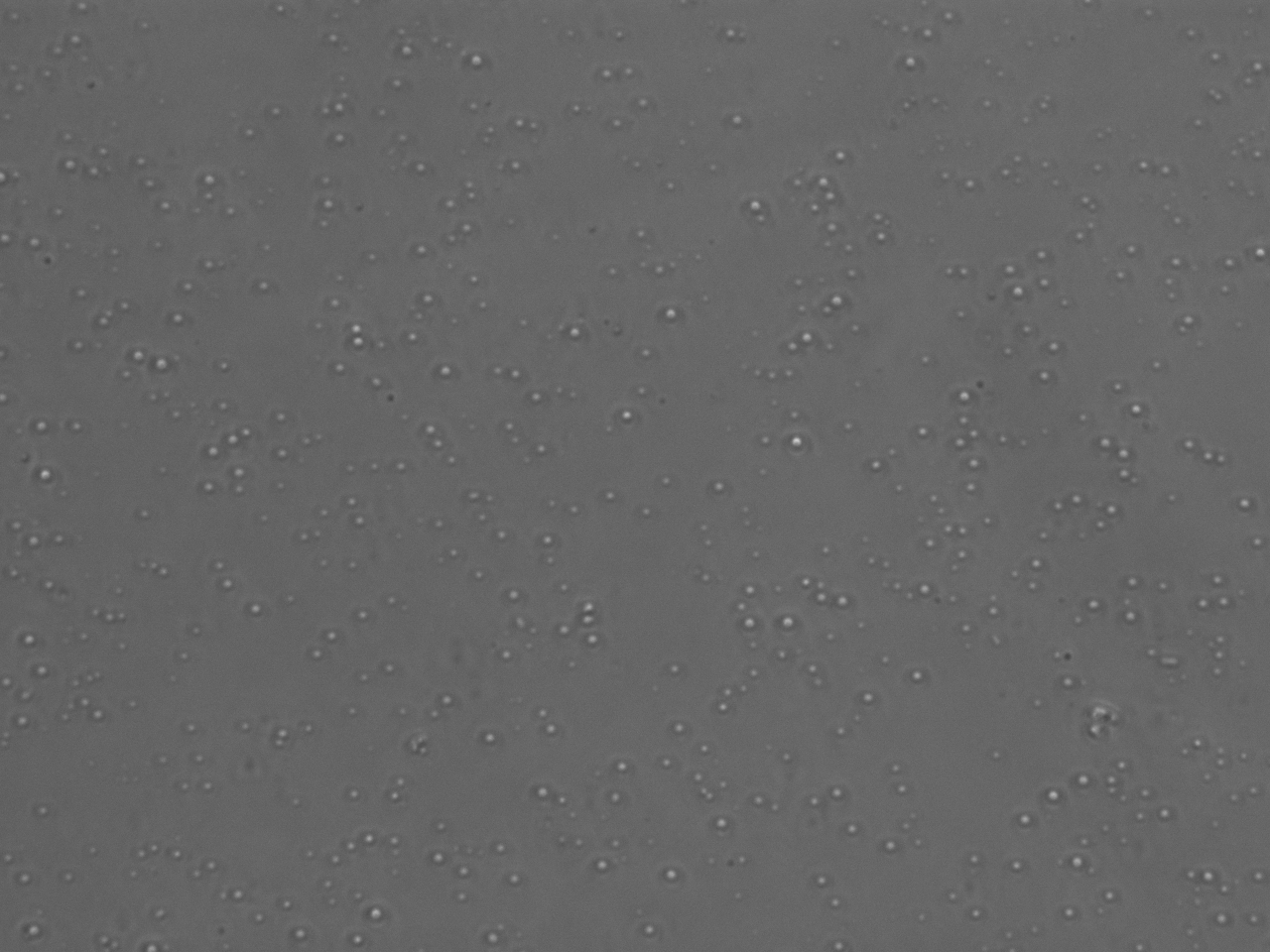

Supplement: Supplementary file 9 — Source Data Fig. 2 [file 44318_2023_21_MOESM9_ESM.zip › Figure 2/Figure 2A/MBP-52K S_D 1.25 uM.tif]

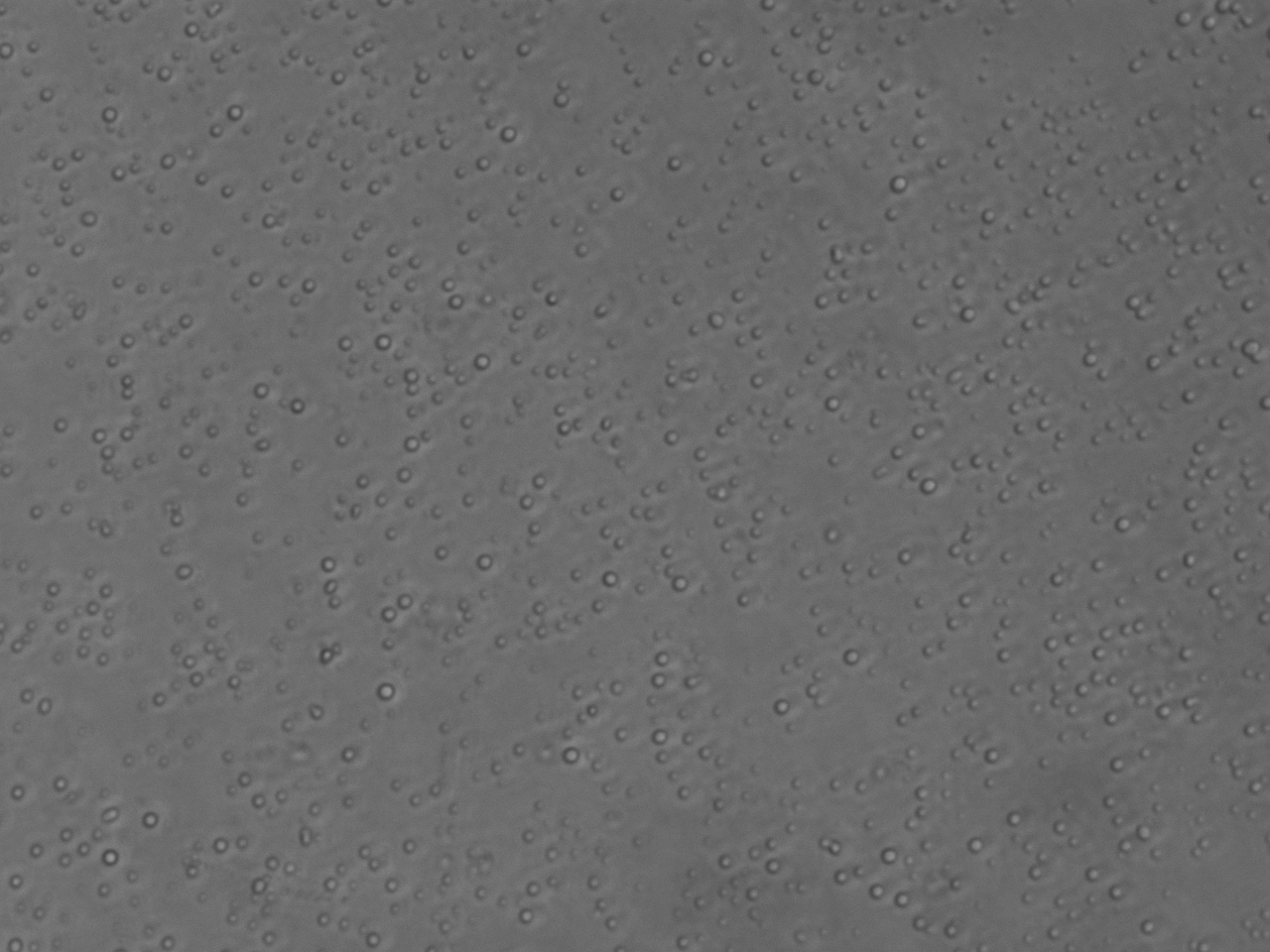

Supplement: Supplementary file 9 — Source Data Fig. 2 [file 44318_2023_21_MOESM9_ESM.zip › Figure 2/Figure 2A/MBP-52K S_D 2.5 uM.tif]

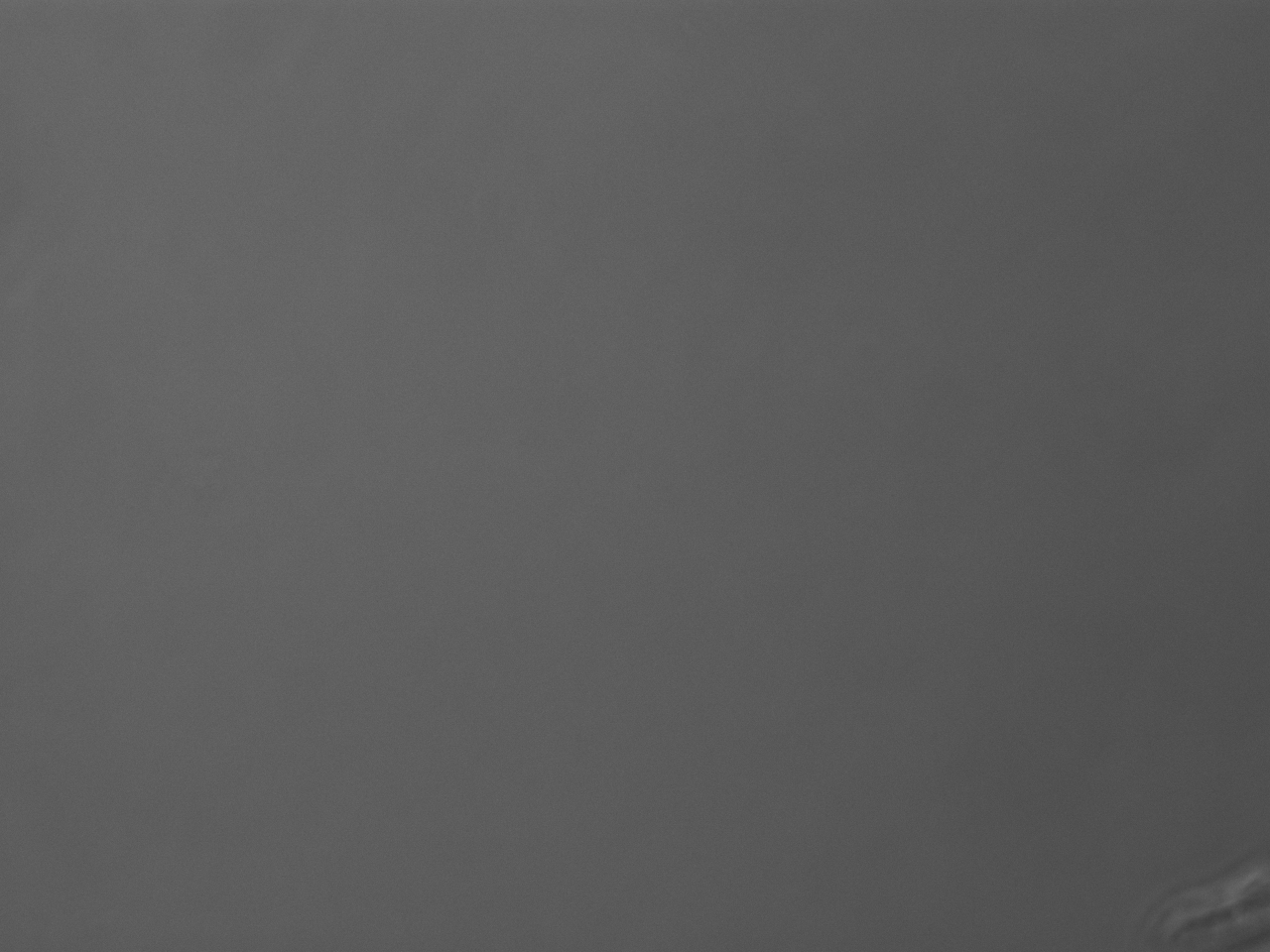

Supplement: Supplementary file 9 — Source Data Fig. 2 [file 44318_2023_21_MOESM9_ESM.zip › Figure 2/Figure 2A/MBP-52K S_D 2.5 uM_- TEV CTL.tif]

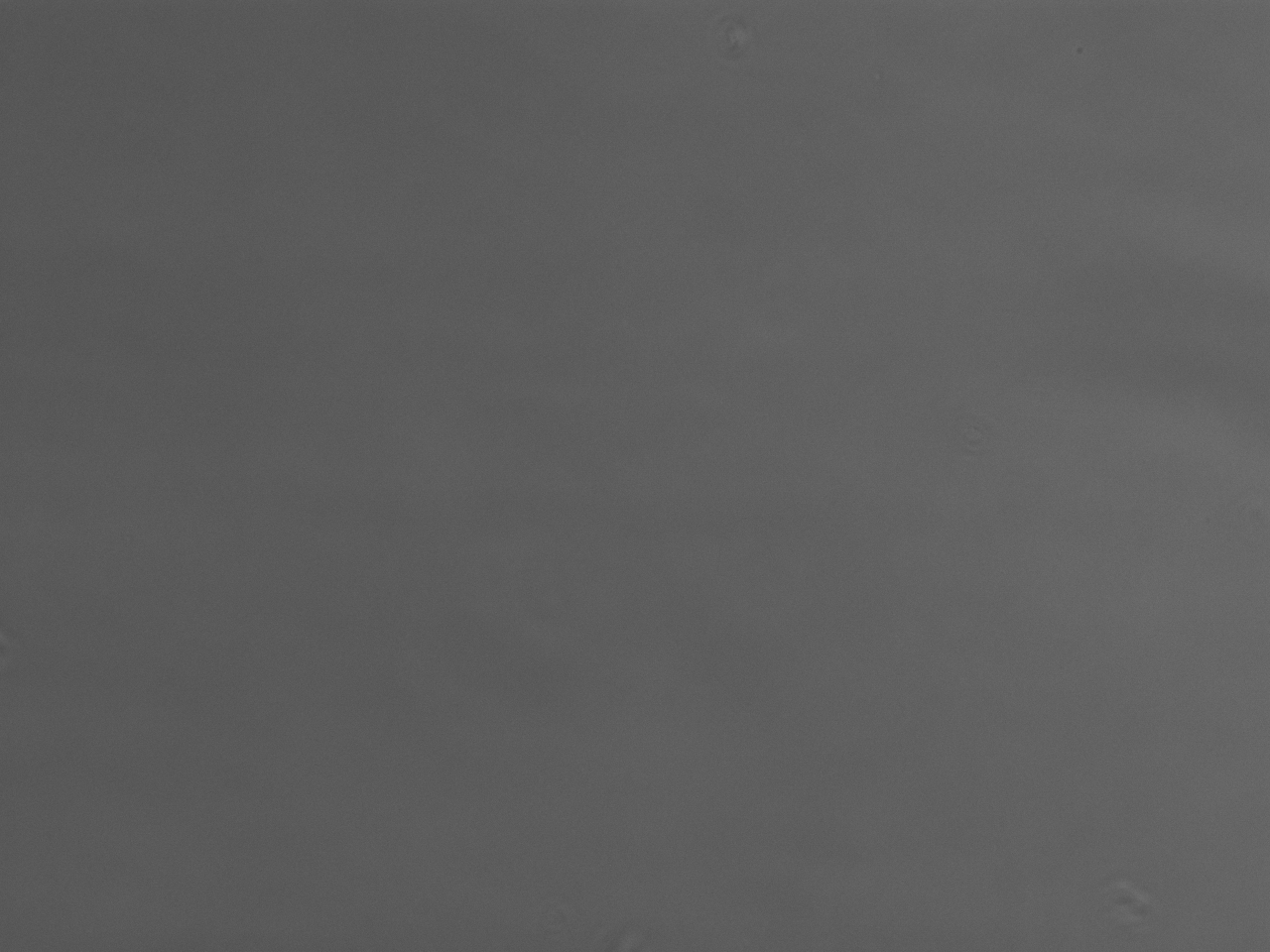

Supplement: Supplementary file 9 — Source Data Fig. 2 [file 44318_2023_21_MOESM9_ESM.zip › Figure 2/Figure 2A/MBP-52K WT 0.3125 uM.tif]

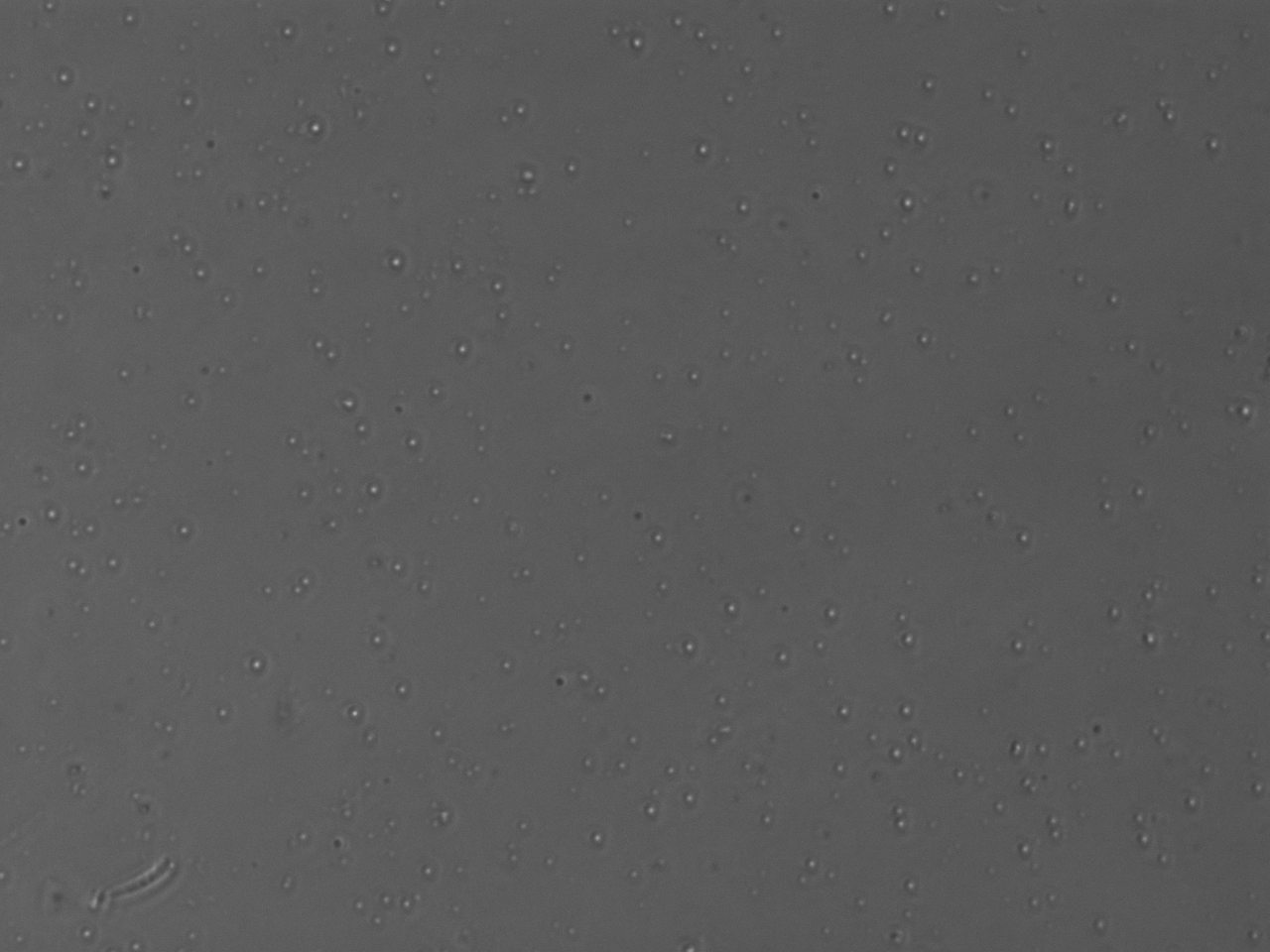

Supplement: Supplementary file 9 — Source Data Fig. 2 [file 44318_2023_21_MOESM9_ESM.zip › Figure 2/Figure 2A/MBP-52K WT 0.625 uM.tif]

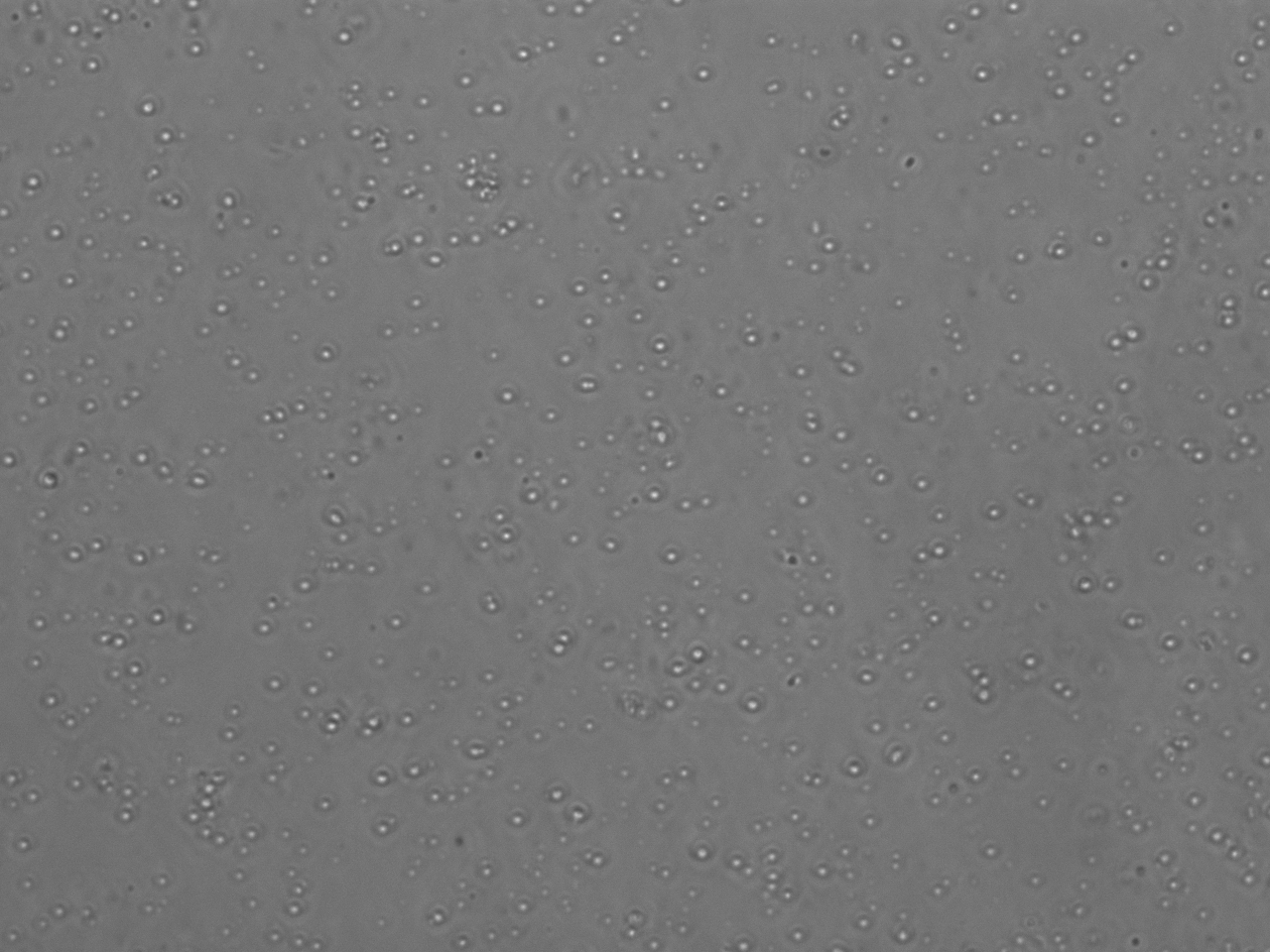

Supplement: Supplementary file 9 — Source Data Fig. 2 [file 44318_2023_21_MOESM9_ESM.zip › Figure 2/Figure 2A/MBP-52K WT 1.25 uM.tif]

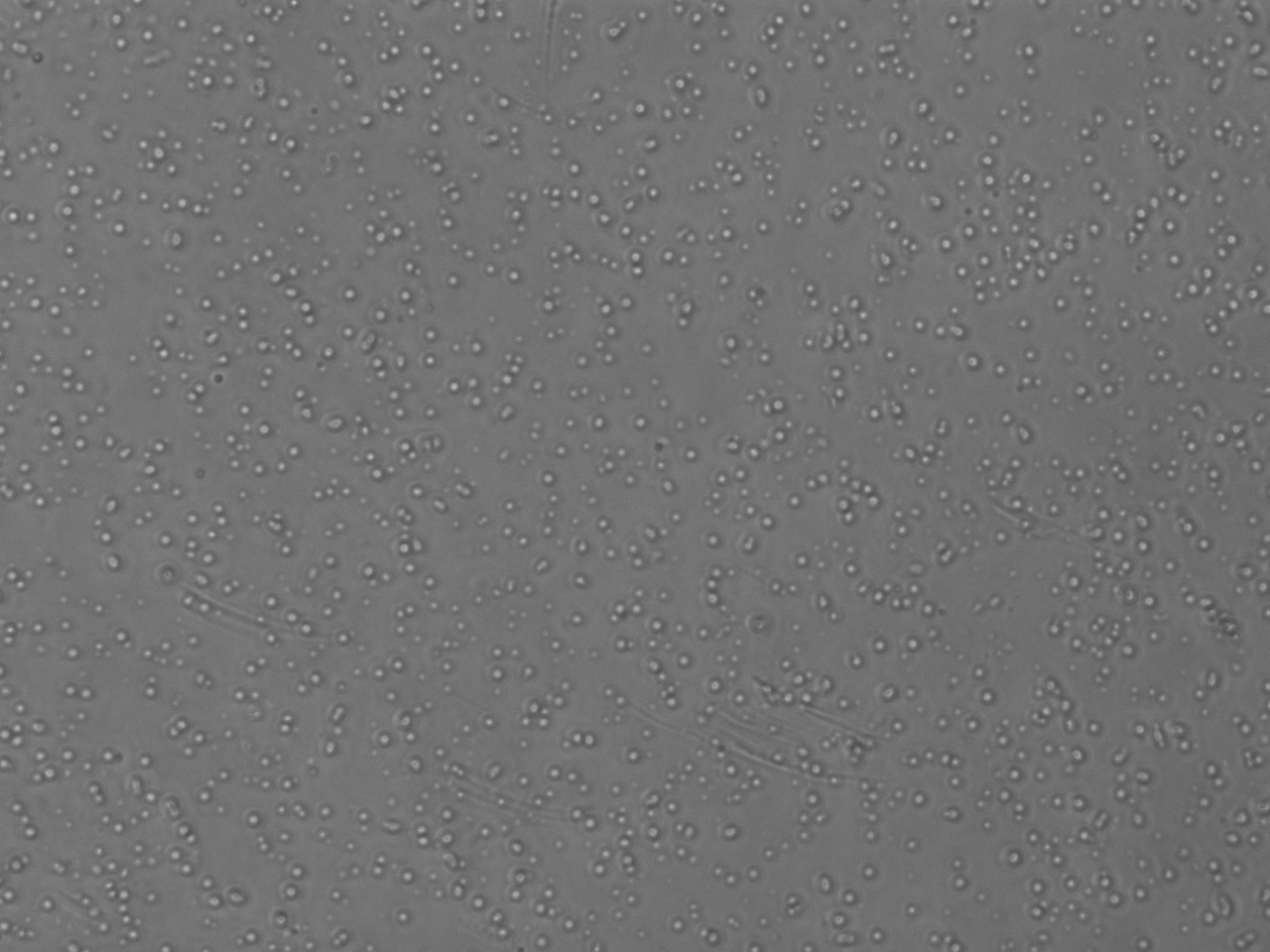

Supplement: Supplementary file 9 — Source Data Fig. 2 [file 44318_2023_21_MOESM9_ESM.zip › Figure 2/Figure 2A/MBP-52K WT 2.5 uM.tif]

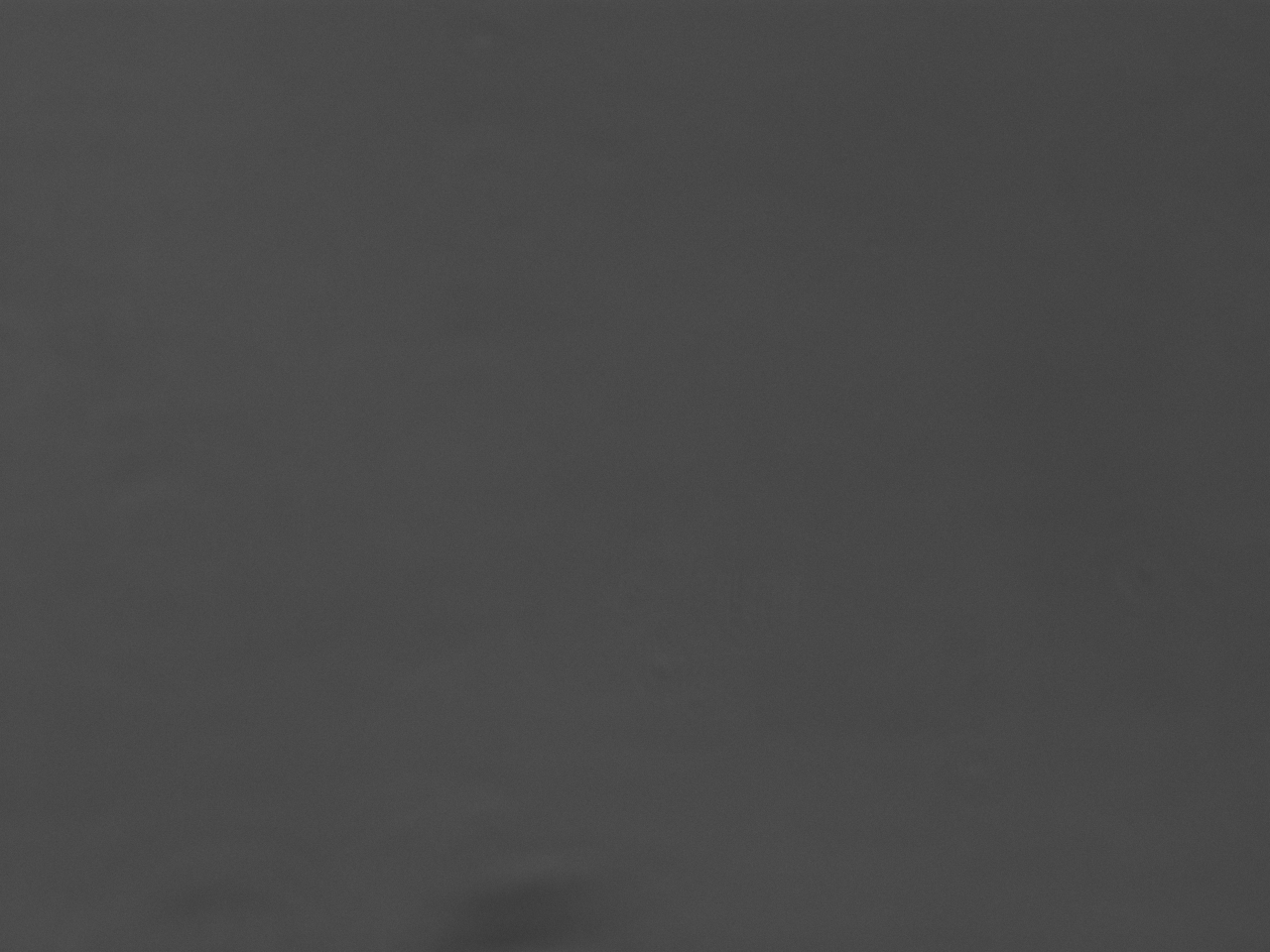

Supplement: Supplementary file 9 — Source Data Fig. 2 [file 44318_2023_21_MOESM9_ESM.zip › Figure 2/Figure 2A/MBP-52K WT 2.5 uM_- TEV CTL.tif]

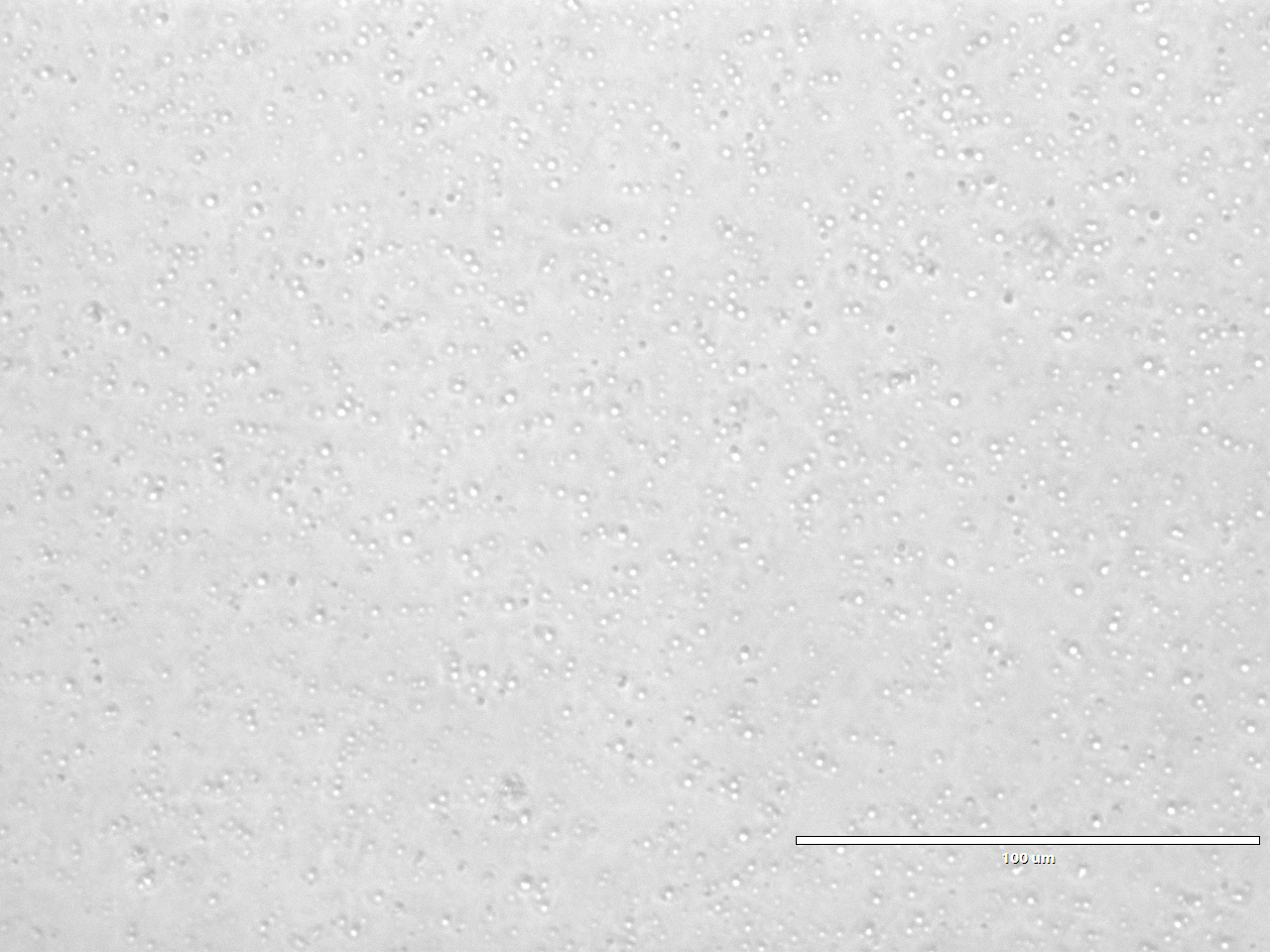

Supplement: Supplementary file 9 — Source Data Fig. 2 [file 44318_2023_21_MOESM9_ESM.zip › Figure 2/Figure 2B/MBP-52K S_A_10uM_20 min.tif]

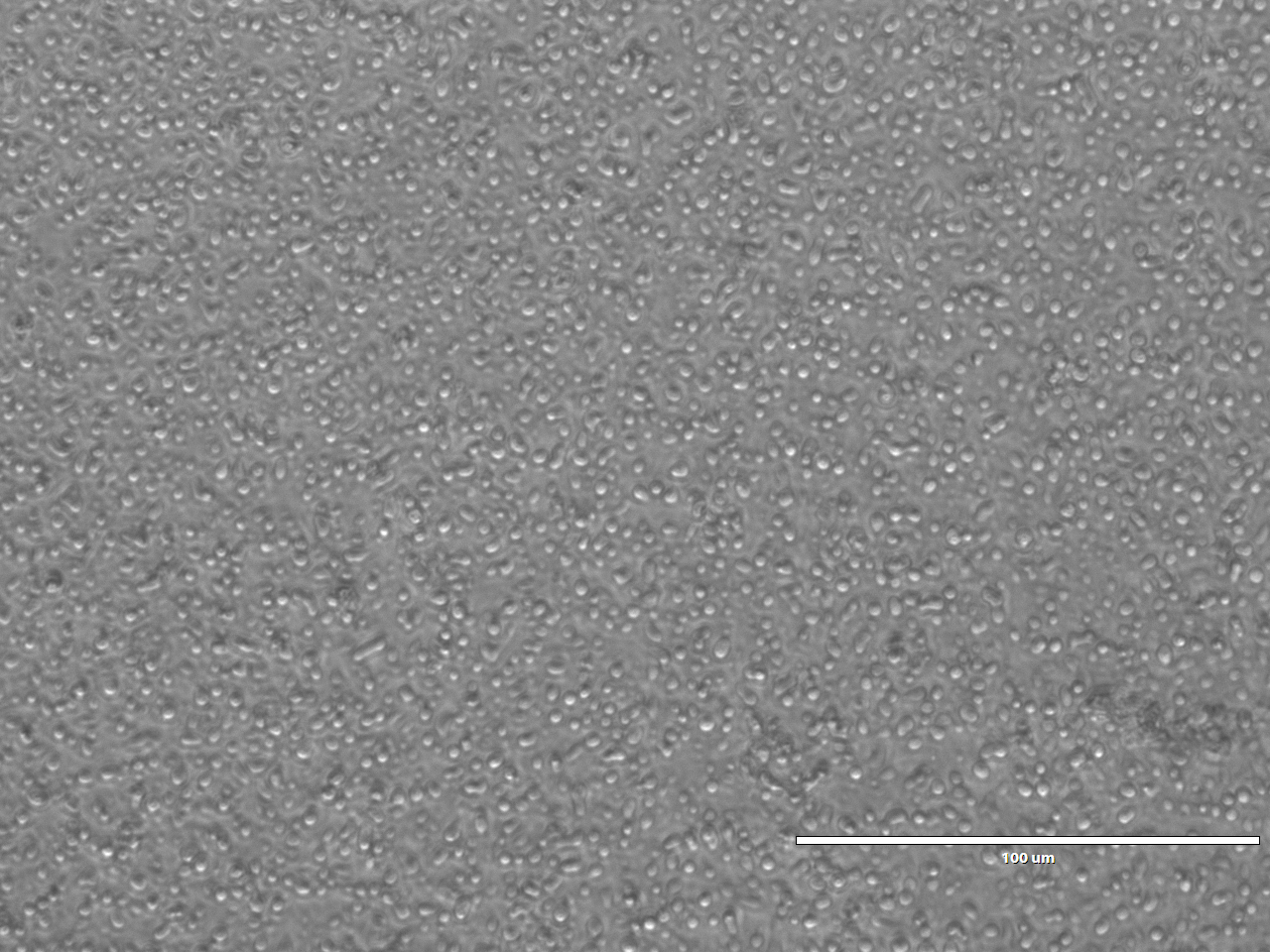

Supplement: Supplementary file 9 — Source Data Fig. 2 [file 44318_2023_21_MOESM9_ESM.zip › Figure 2/Figure 2B/MBP-52K S_A_10uM_2hr.tif]

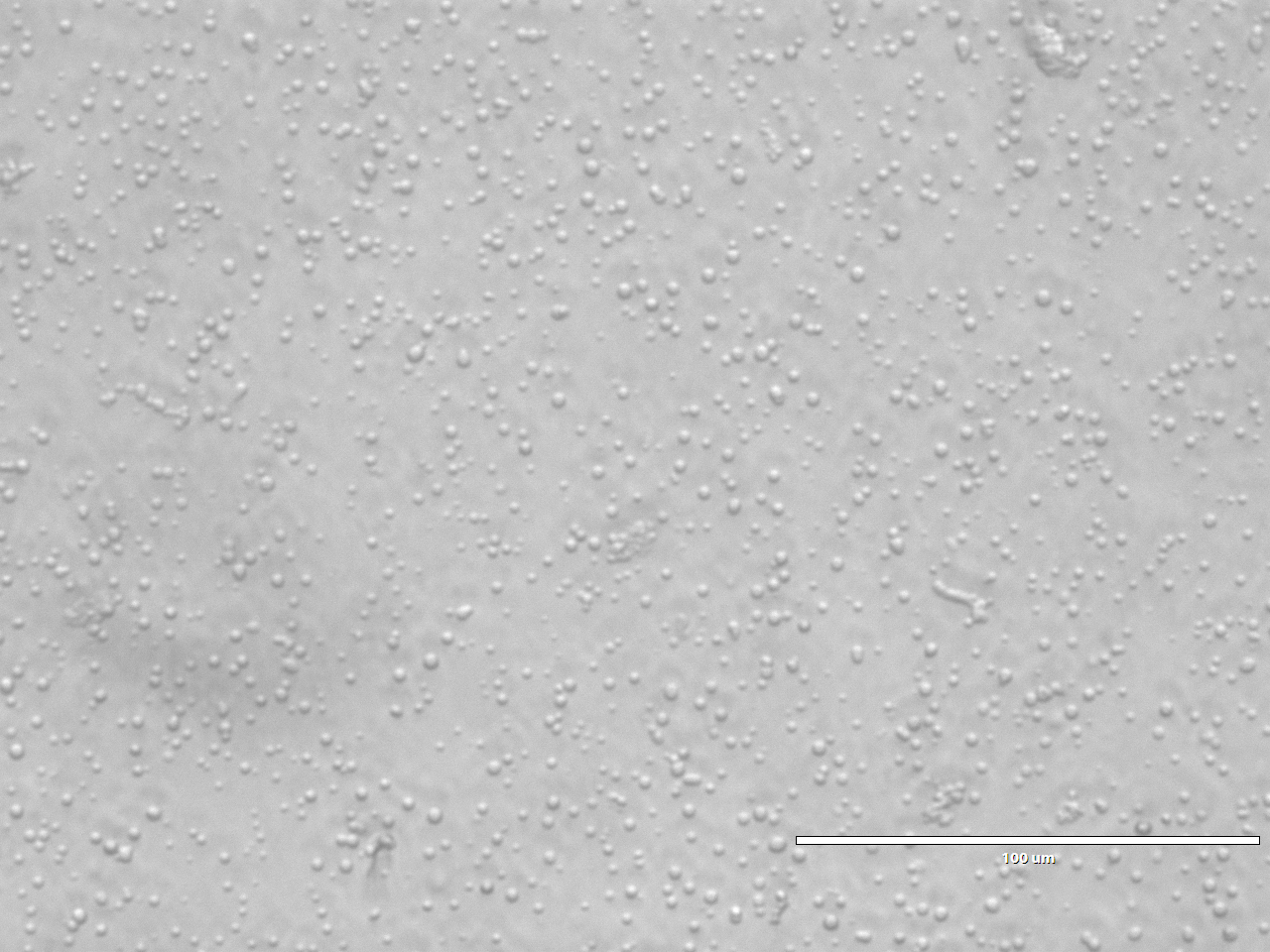

Supplement: Supplementary file 9 — Source Data Fig. 2 [file 44318_2023_21_MOESM9_ESM.zip › Figure 2/Figure 2B/MBP-52K S_D_10uM_20 min.tif]

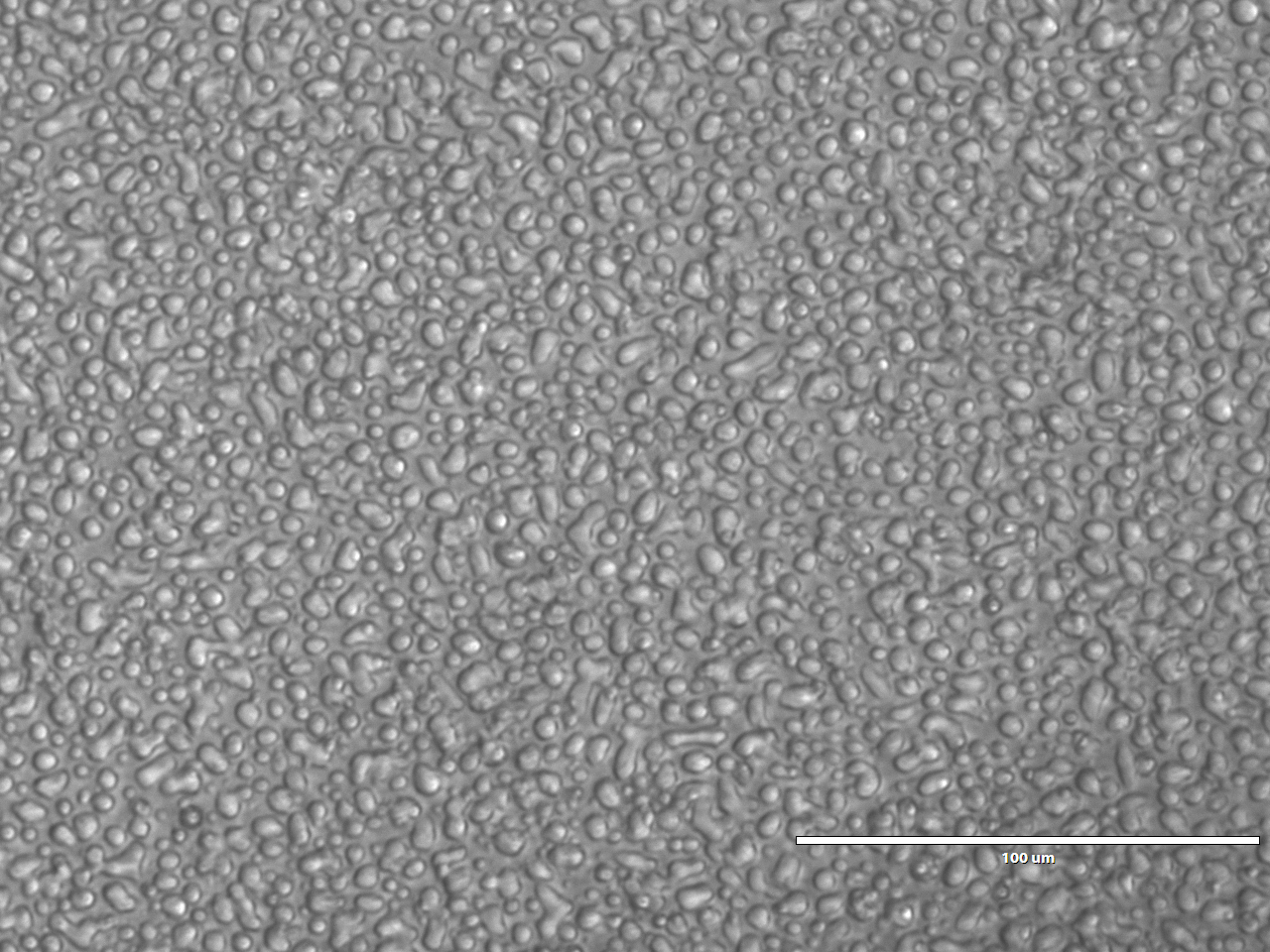

Supplement: Supplementary file 9 — Source Data Fig. 2 [file 44318_2023_21_MOESM9_ESM.zip › Figure 2/Figure 2B/MBP-52K S_D_10uM_2hr.tif]

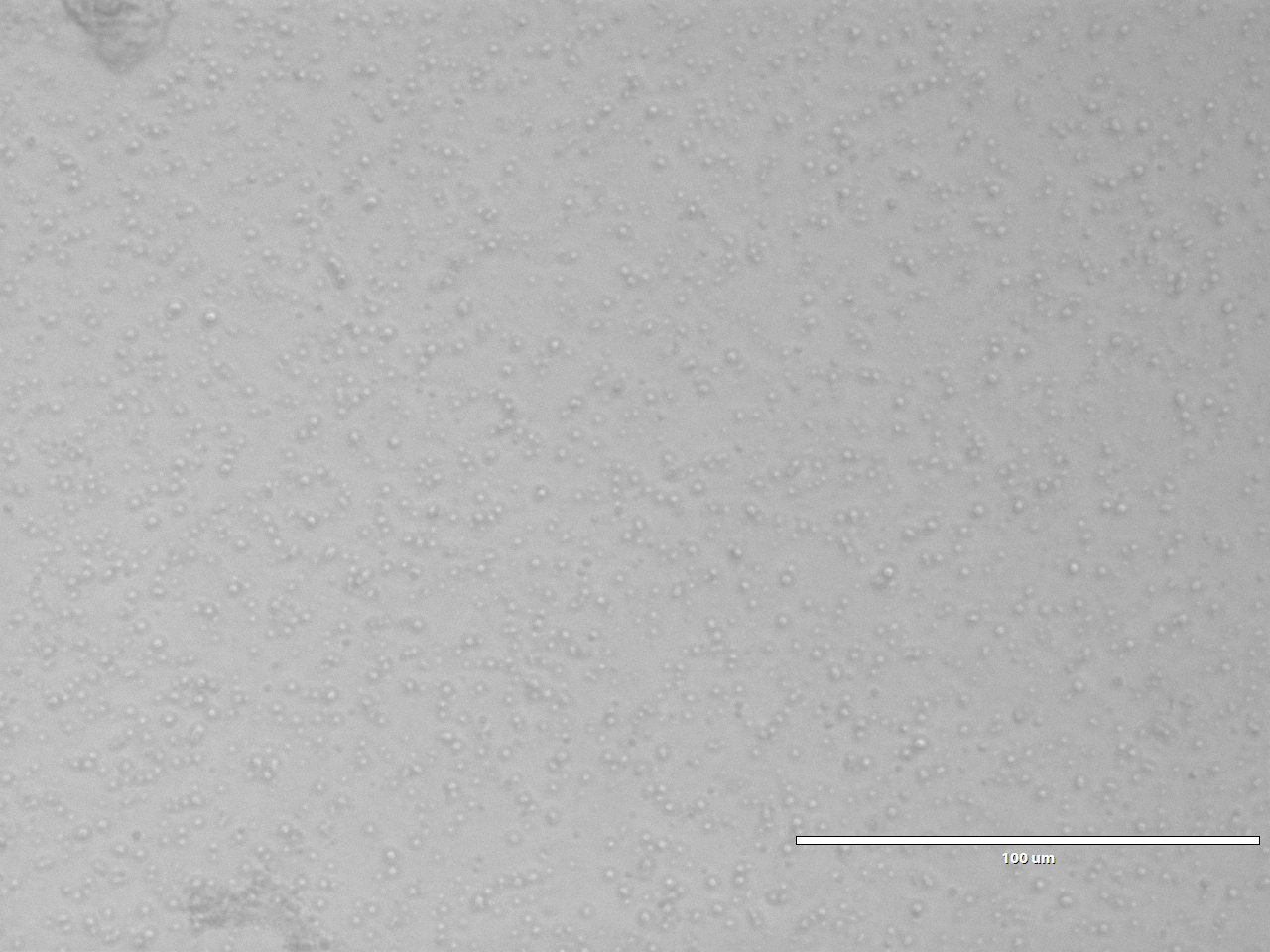

Supplement: Supplementary file 9 — Source Data Fig. 2 [file 44318_2023_21_MOESM9_ESM.zip › Figure 2/Figure 2B/MBP-52K WT_10uM_20 min.tif]

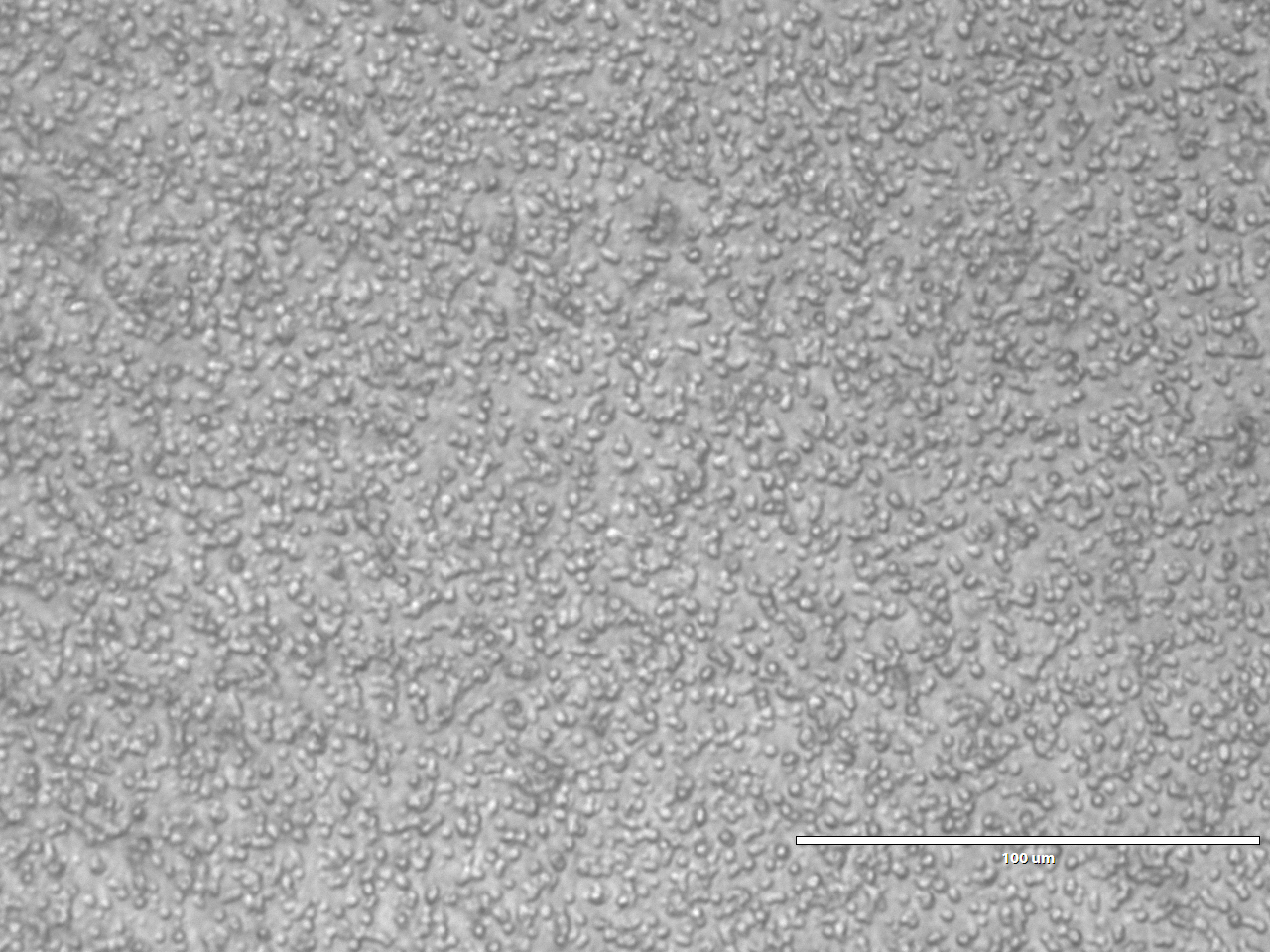

Supplement: Supplementary file 9 — Source Data Fig. 2 [file 44318_2023_21_MOESM9_ESM.zip › Figure 2/Figure 2B/MBP-52K WT_10uM_2hr.tif]

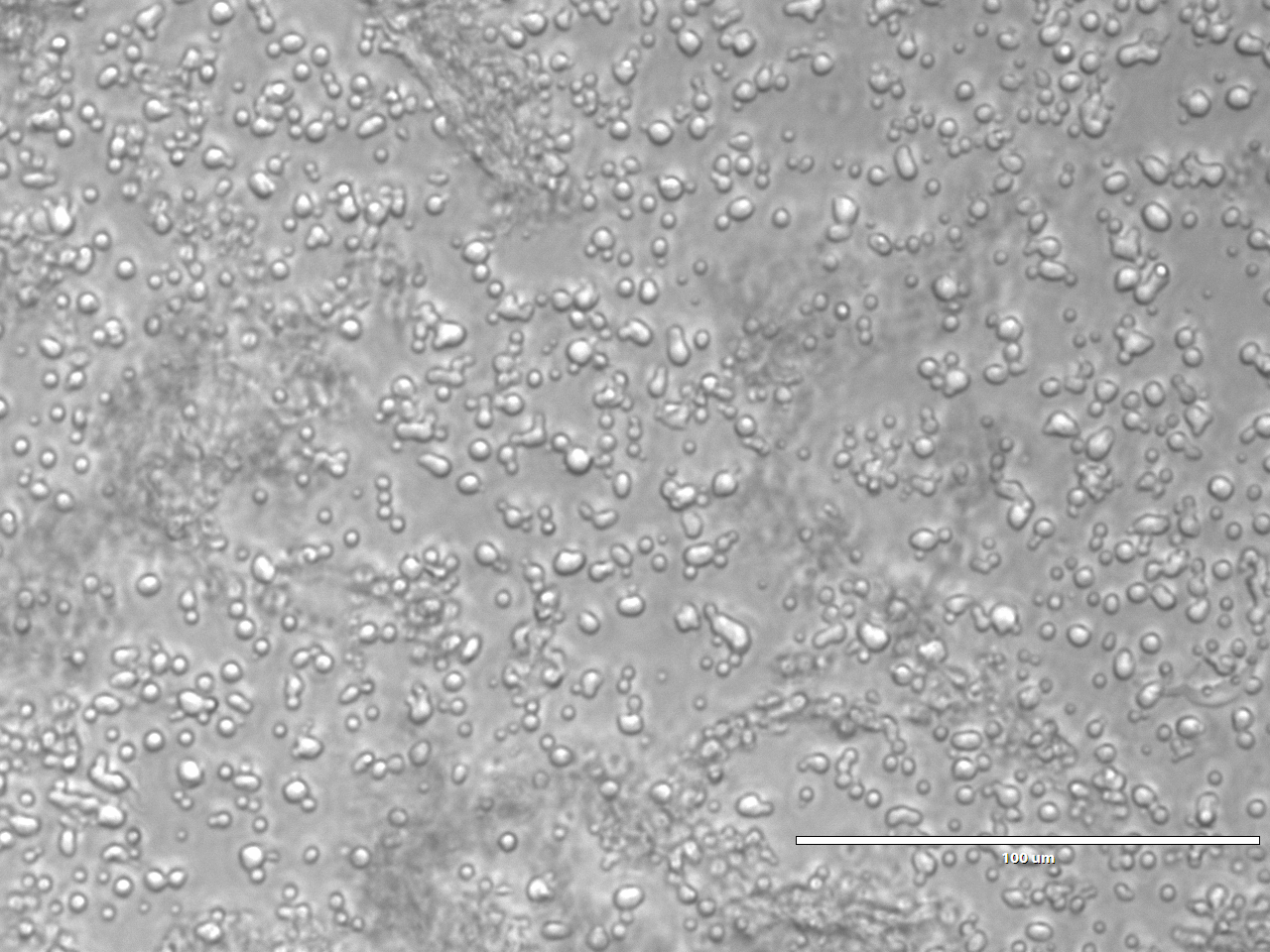

Supplement: Supplementary file 9 — Source Data Fig. 2 [file 44318_2023_21_MOESM9_ESM.zip › Figure 2/Figure 2C/MBP-52K S145_336A_10uM_JNK2_2 hrs.tif]

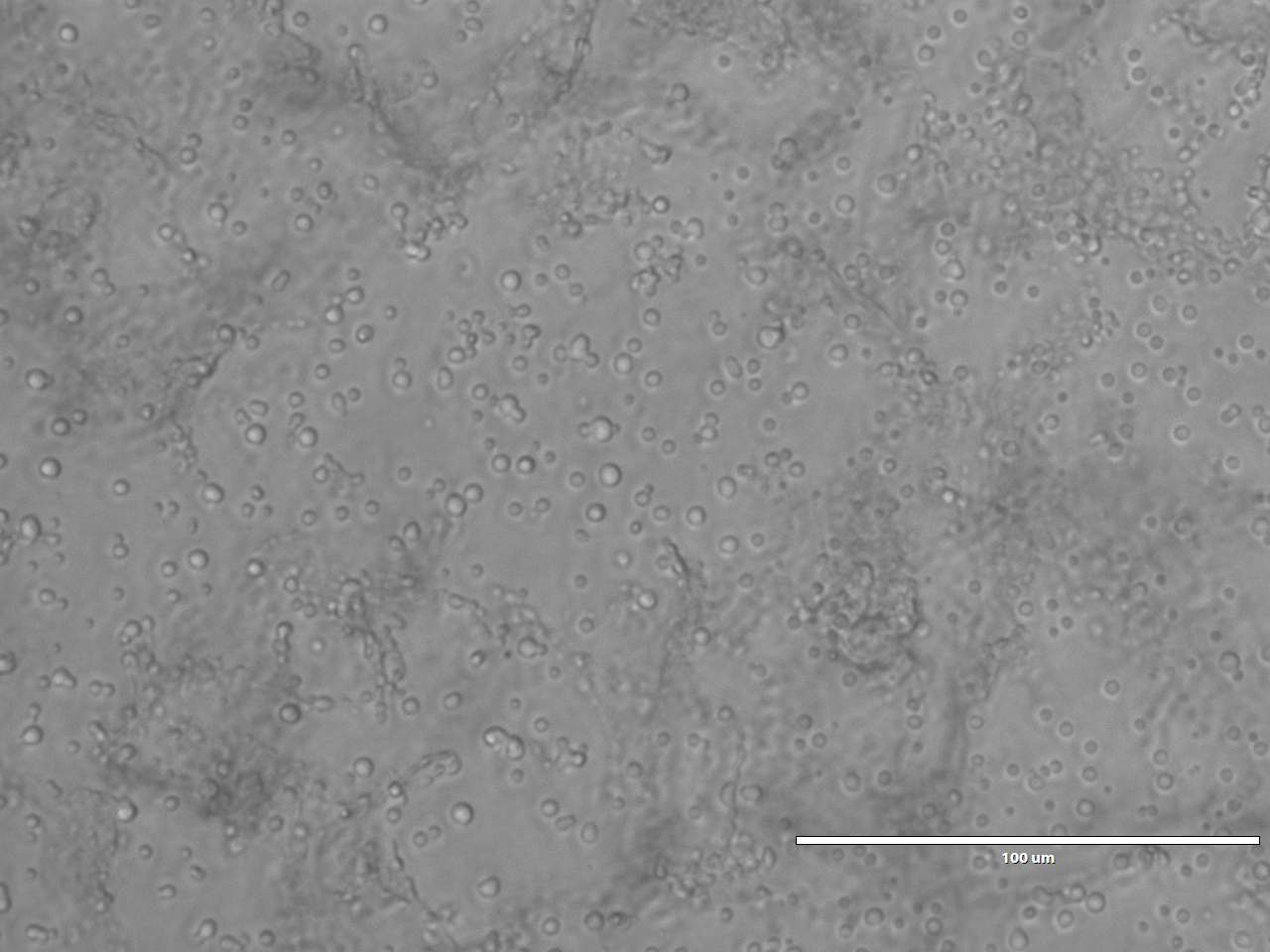

Supplement: Supplementary file 9 — Source Data Fig. 2 [file 44318_2023_21_MOESM9_ESM.zip › Figure 2/Figure 2C/MBP-52K S145_336A_10uM_JNK2_20 mins.tif]

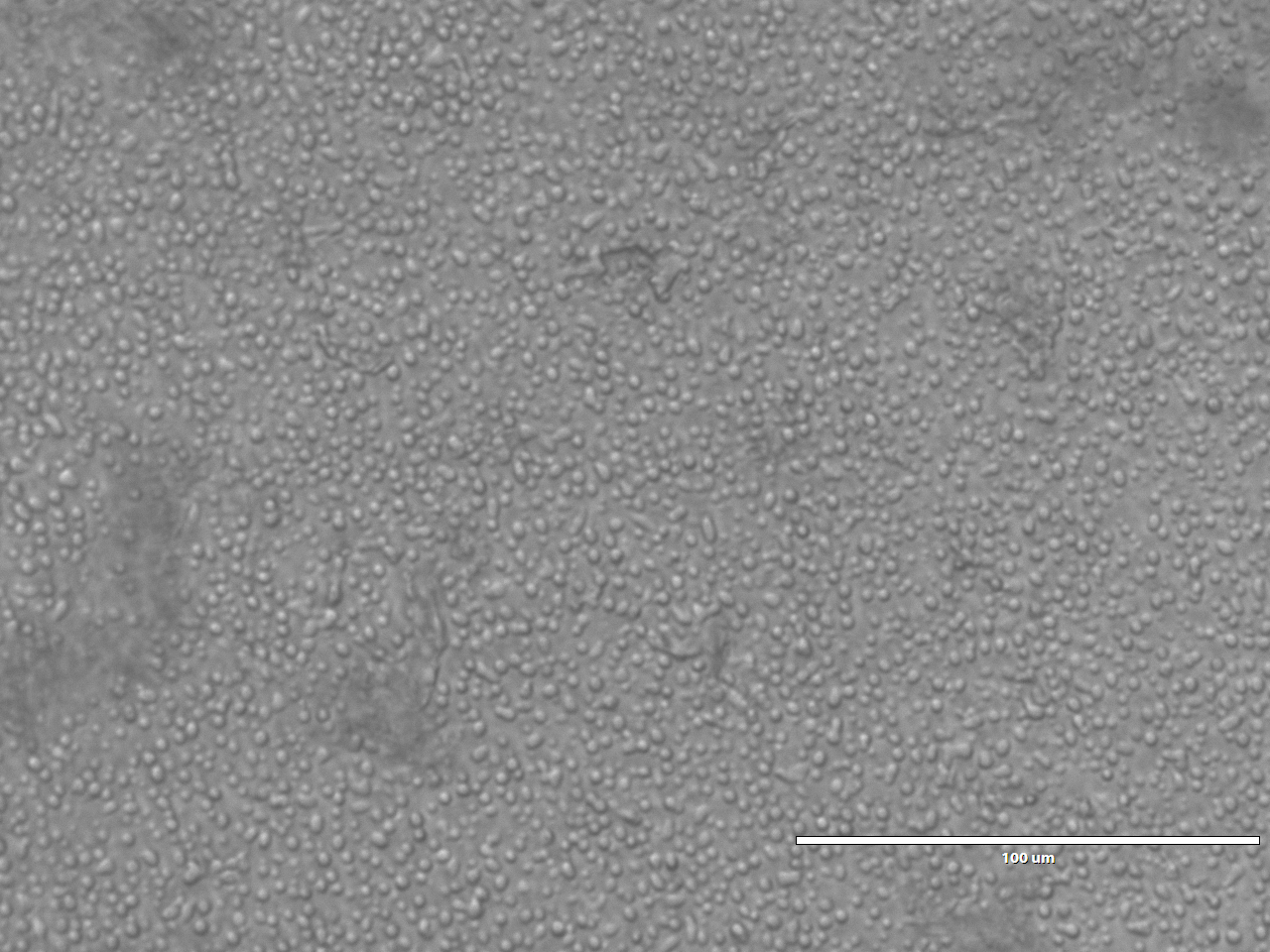

Supplement: Supplementary file 9 — Source Data Fig. 2 [file 44318_2023_21_MOESM9_ESM.zip › Figure 2/Figure 2C/MBP-52K S145_336A_10uM_No kinase_2 hrs.tif]

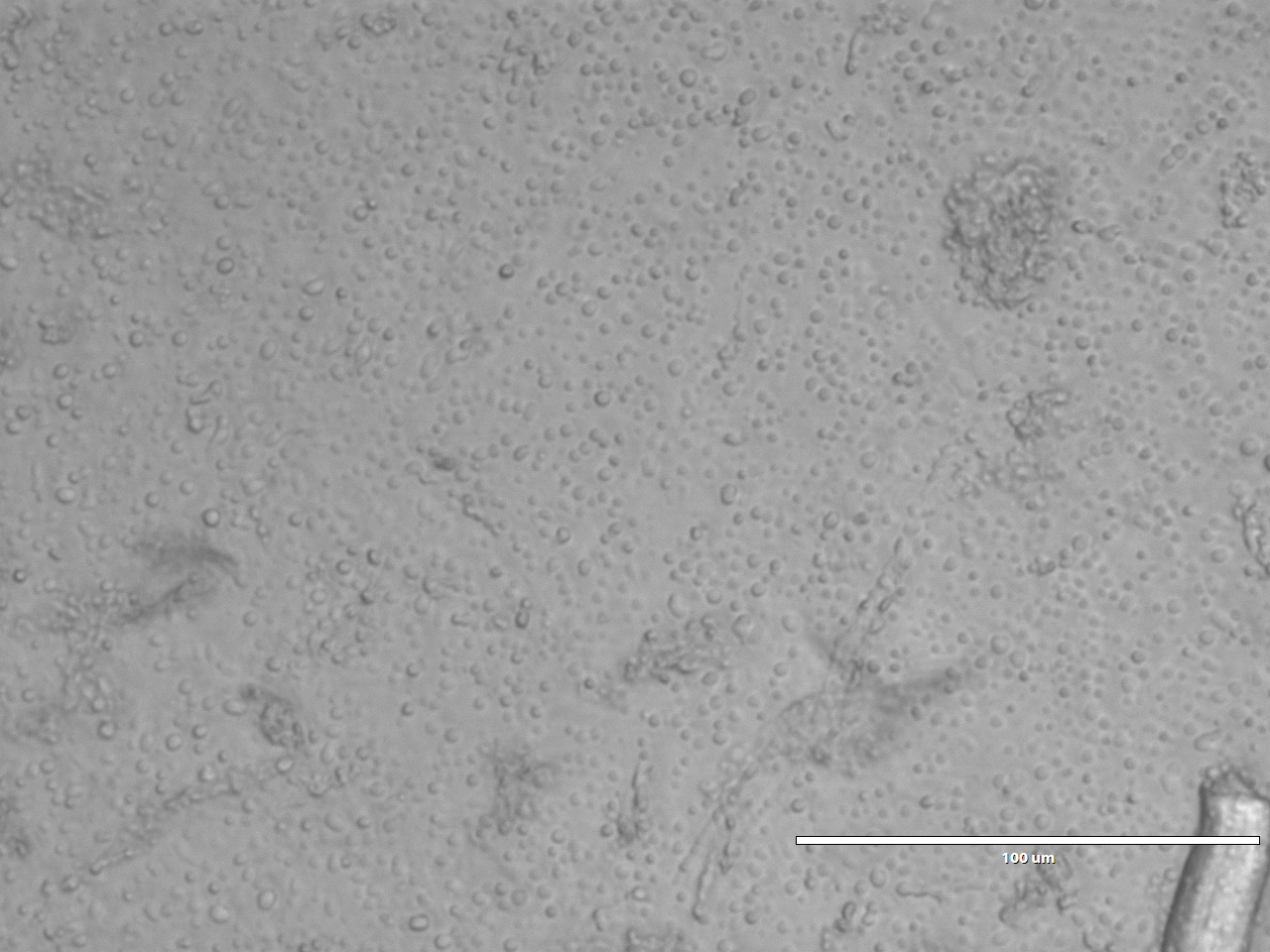

Supplement: Supplementary file 9 — Source Data Fig. 2 [file 44318_2023_21_MOESM9_ESM.zip › Figure 2/Figure 2C/MBP-52K S145_336A_10uM_No kinase_20 mins.tif]

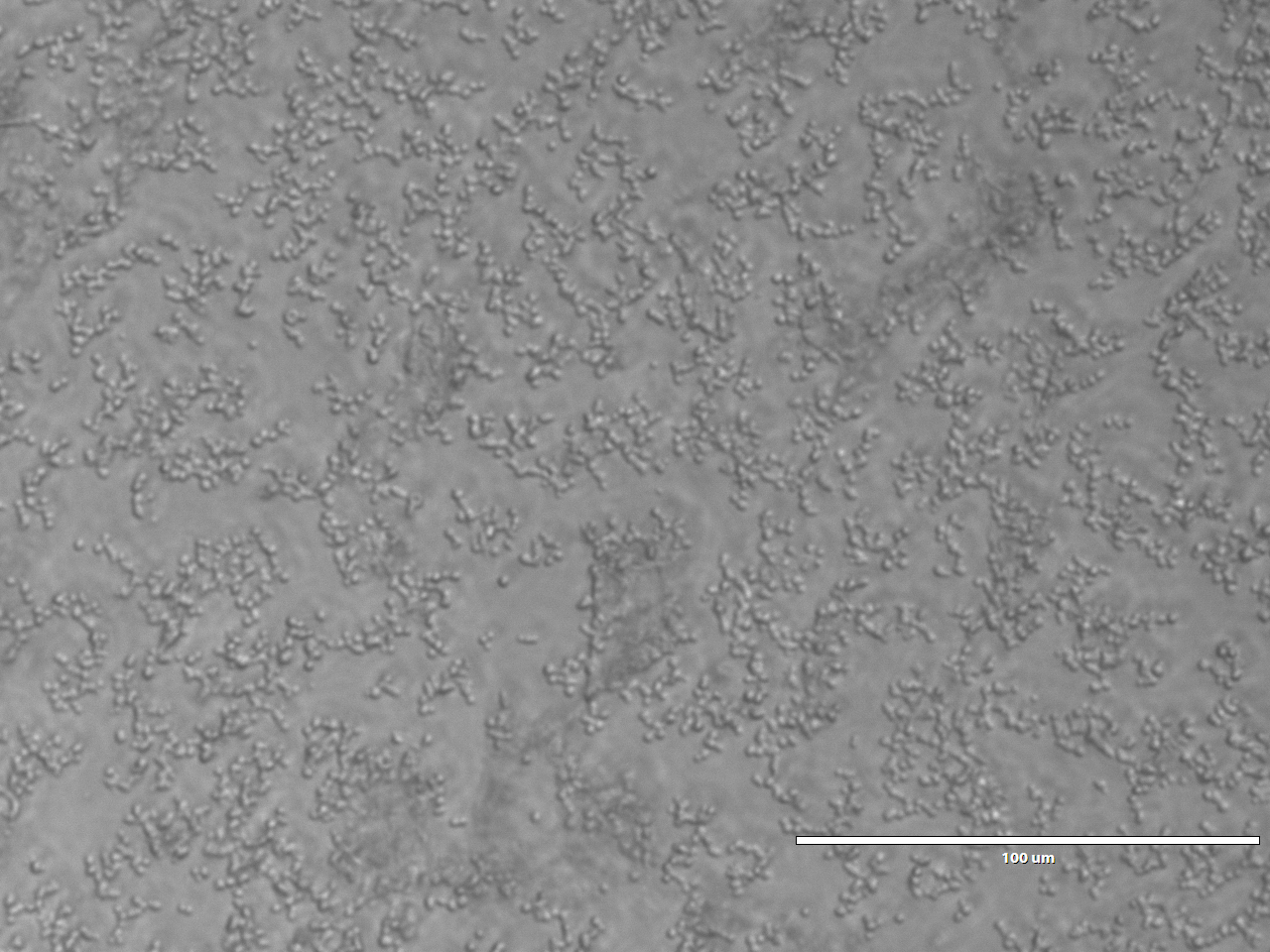

Supplement: Supplementary file 9 — Source Data Fig. 2 [file 44318_2023_21_MOESM9_ESM.zip › Figure 2/Figure 2C/MBP-52K S28_75A_10uM_JNK2_2 hrs.tif]

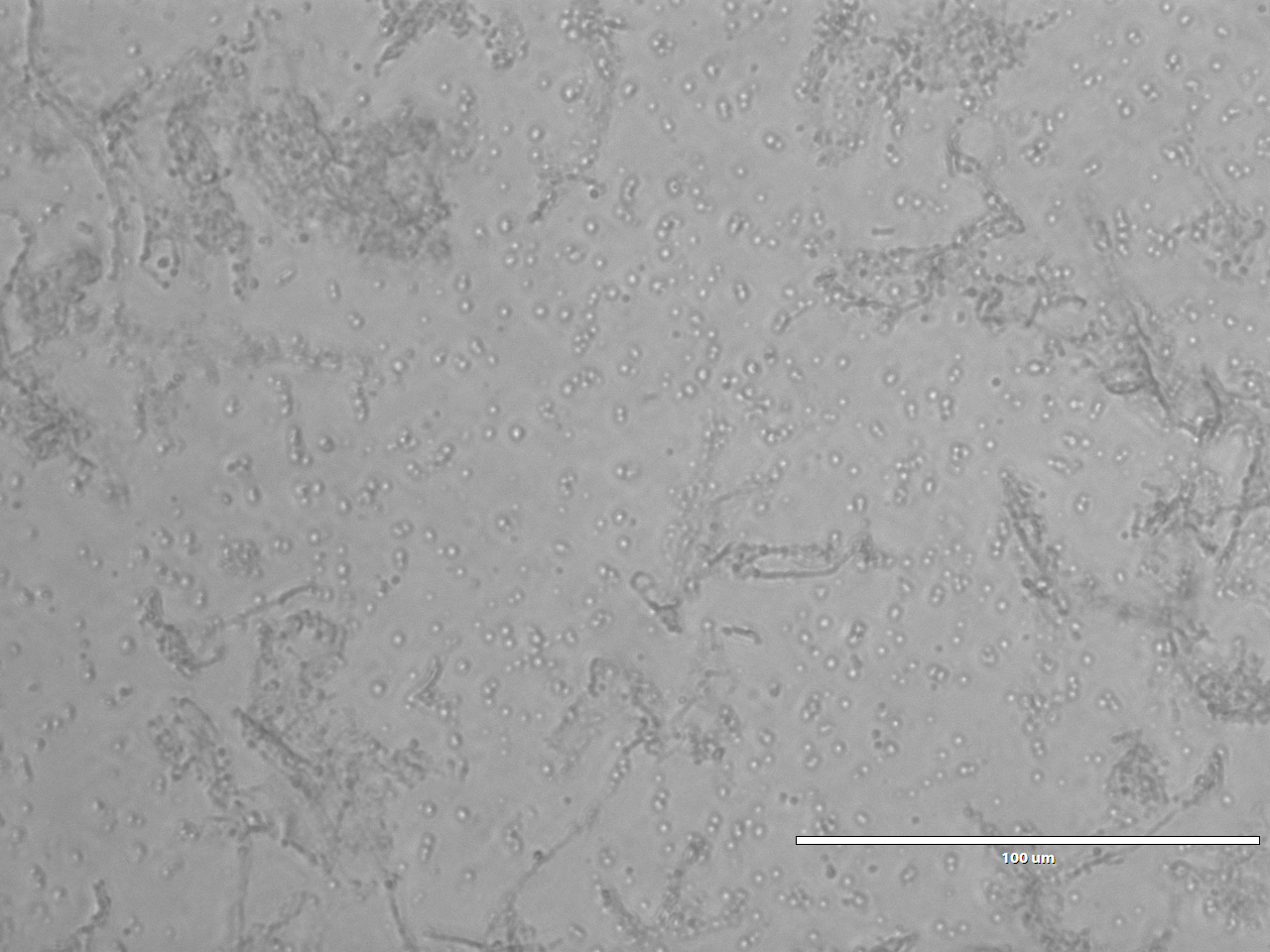

Supplement: Supplementary file 9 — Source Data Fig. 2 [file 44318_2023_21_MOESM9_ESM.zip › Figure 2/Figure 2C/MBP-52K S28_75A_10uM_JNK2_20 mins.tif]

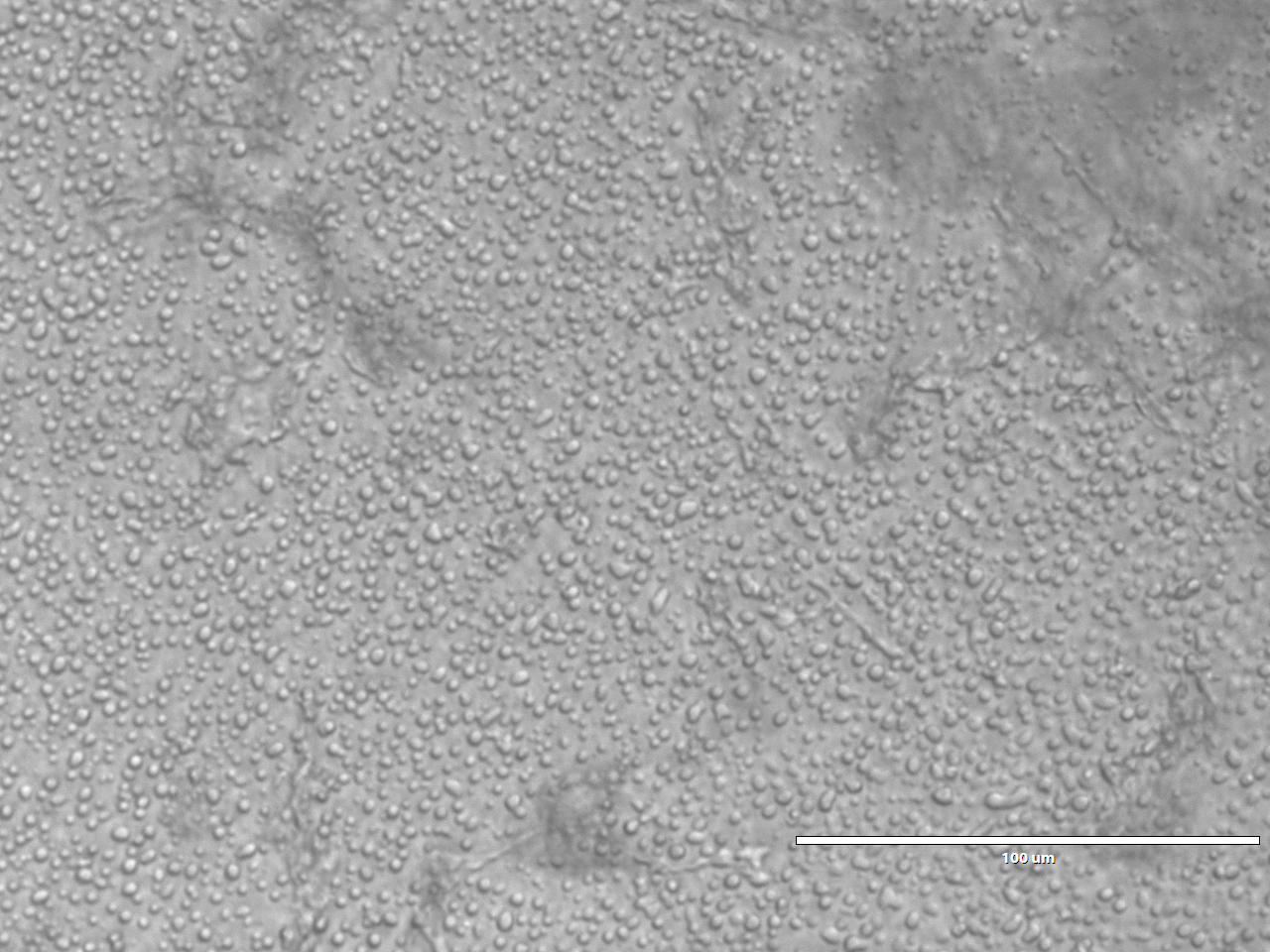

Supplement: Supplementary file 9 — Source Data Fig. 2 [file 44318_2023_21_MOESM9_ESM.zip › Figure 2/Figure 2C/MBP-52K S28_75A_10uM_No Kinase_2 hrs.tif]

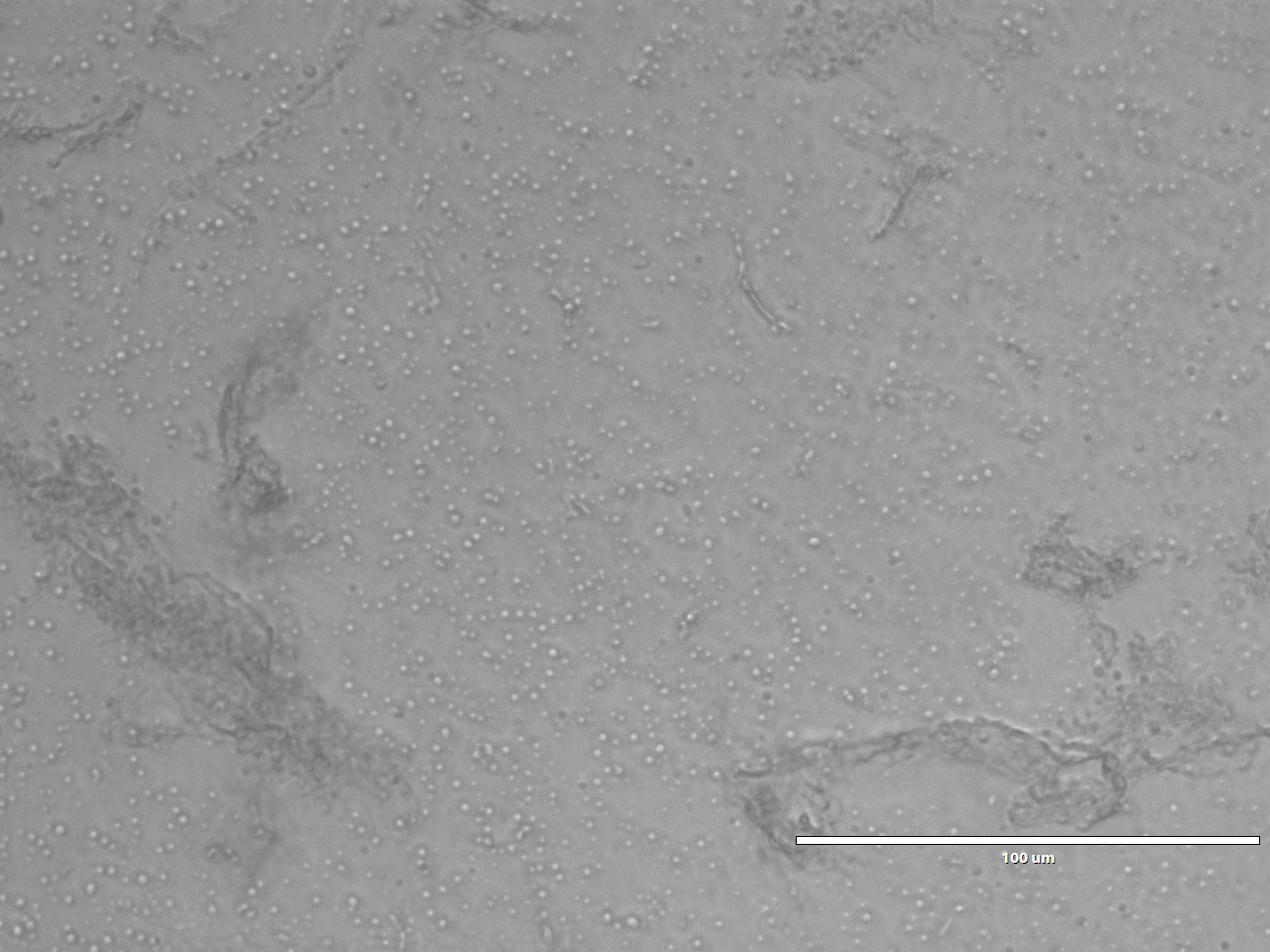

Supplement: Supplementary file 9 — Source Data Fig. 2 [file 44318_2023_21_MOESM9_ESM.zip › Figure 2/Figure 2C/MBP-52K S28_75A_10uM_No Kinase_20 mins.tif]

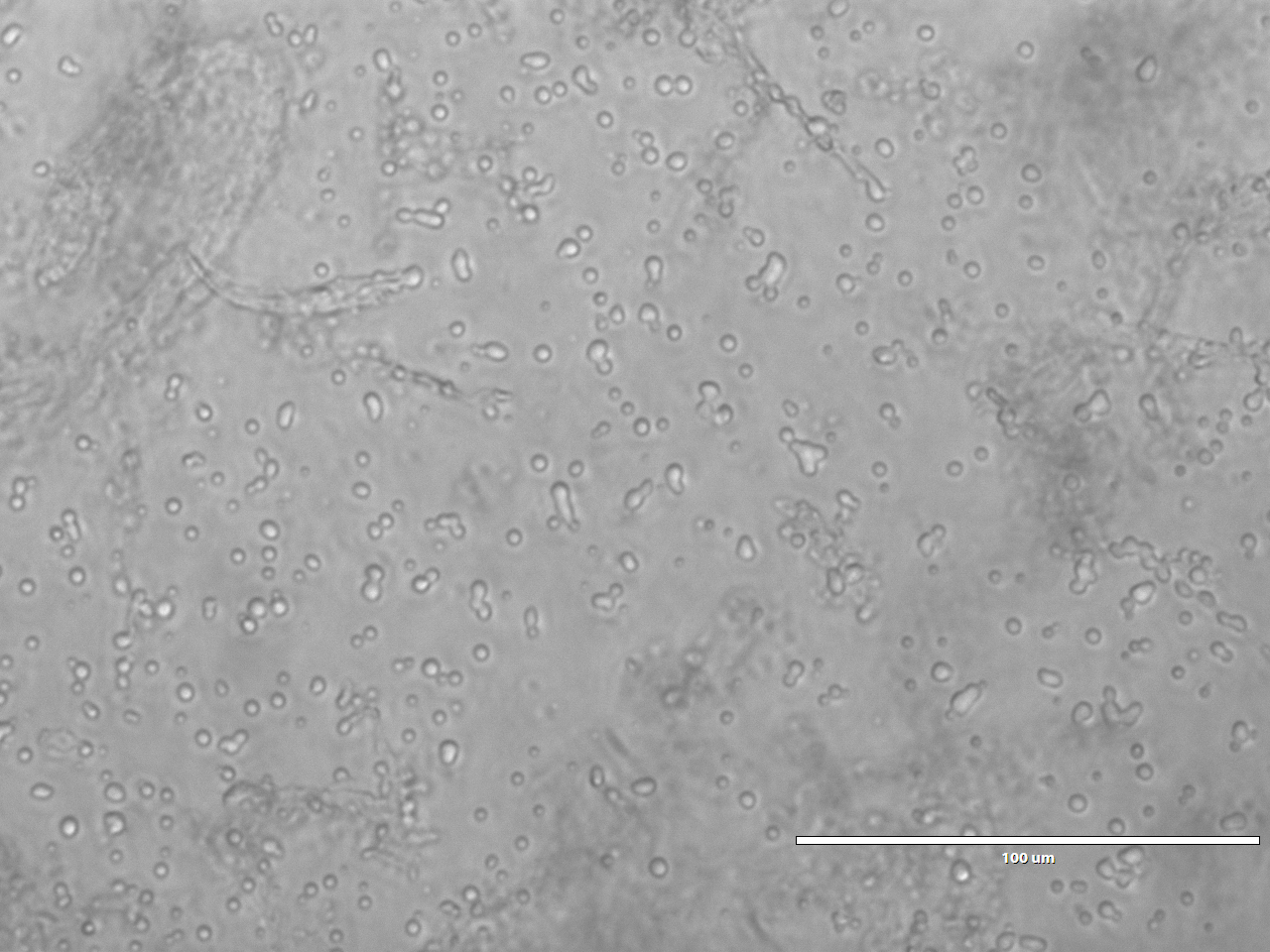

Supplement: Supplementary file 9 — Source Data Fig. 2 [file 44318_2023_21_MOESM9_ESM.zip › Figure 2/Figure 2C/MBP-52K WT_10uM_JNK2_20 mins.tif]

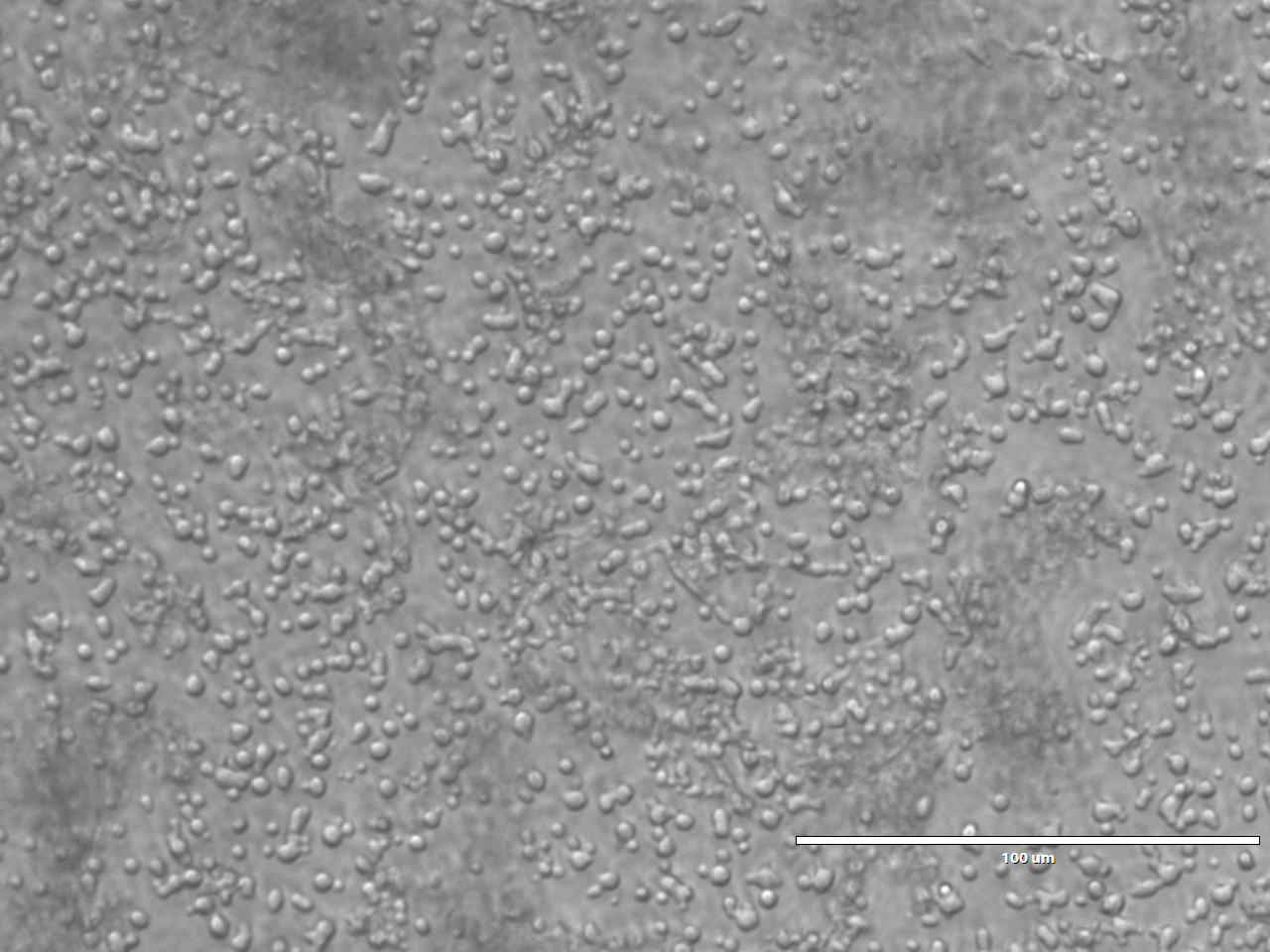

Supplement: Supplementary file 9 — Source Data Fig. 2 [file 44318_2023_21_MOESM9_ESM.zip › Figure 2/Figure 2C/MBP-52K WT_10uM_JNK2_2hrs.tif]

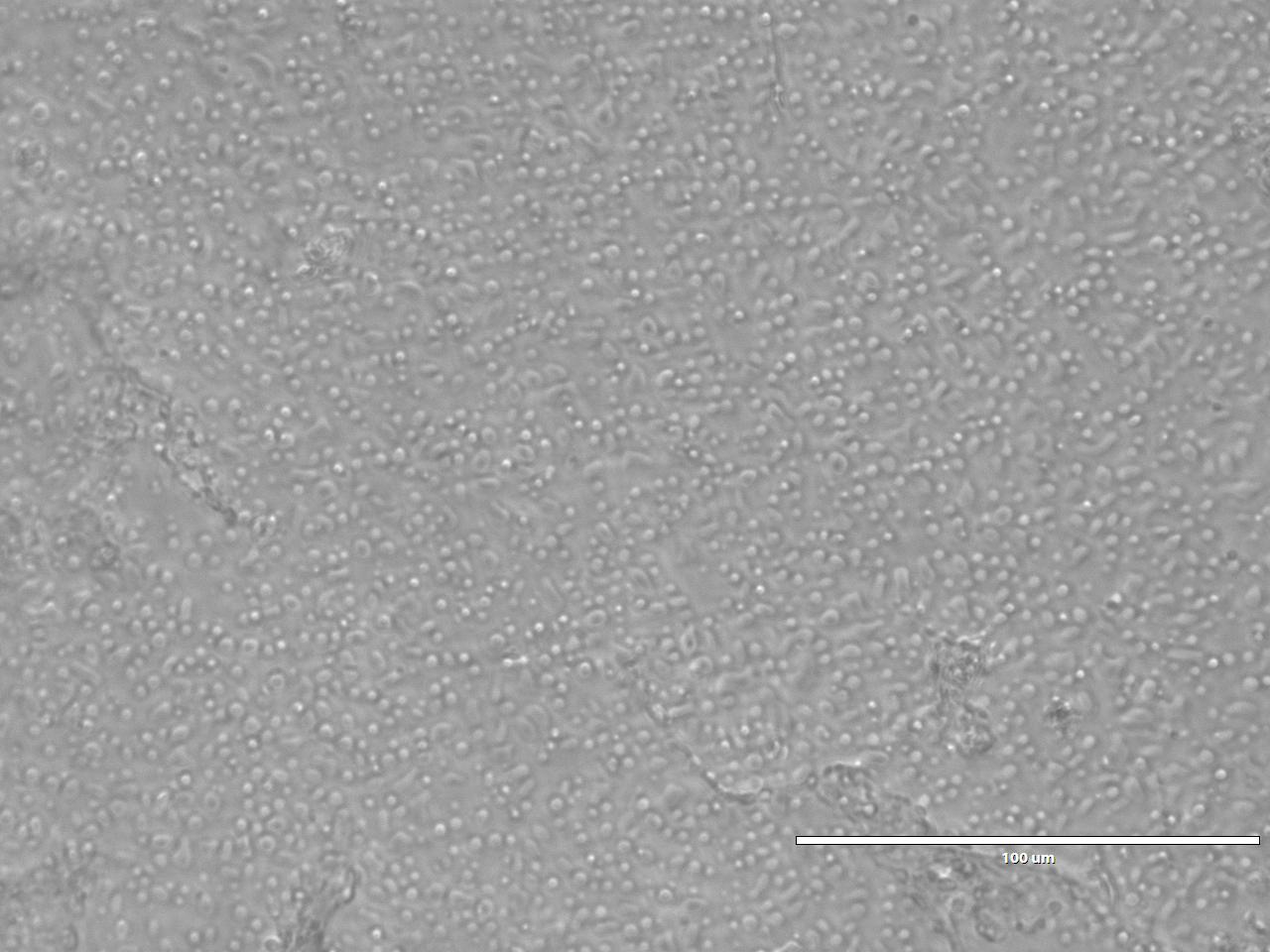

Supplement: Supplementary file 9 — Source Data Fig. 2 [file 44318_2023_21_MOESM9_ESM.zip › Figure 2/Figure 2C/MBP-52K WT_10uM_No kinase_2 hrs.tif]

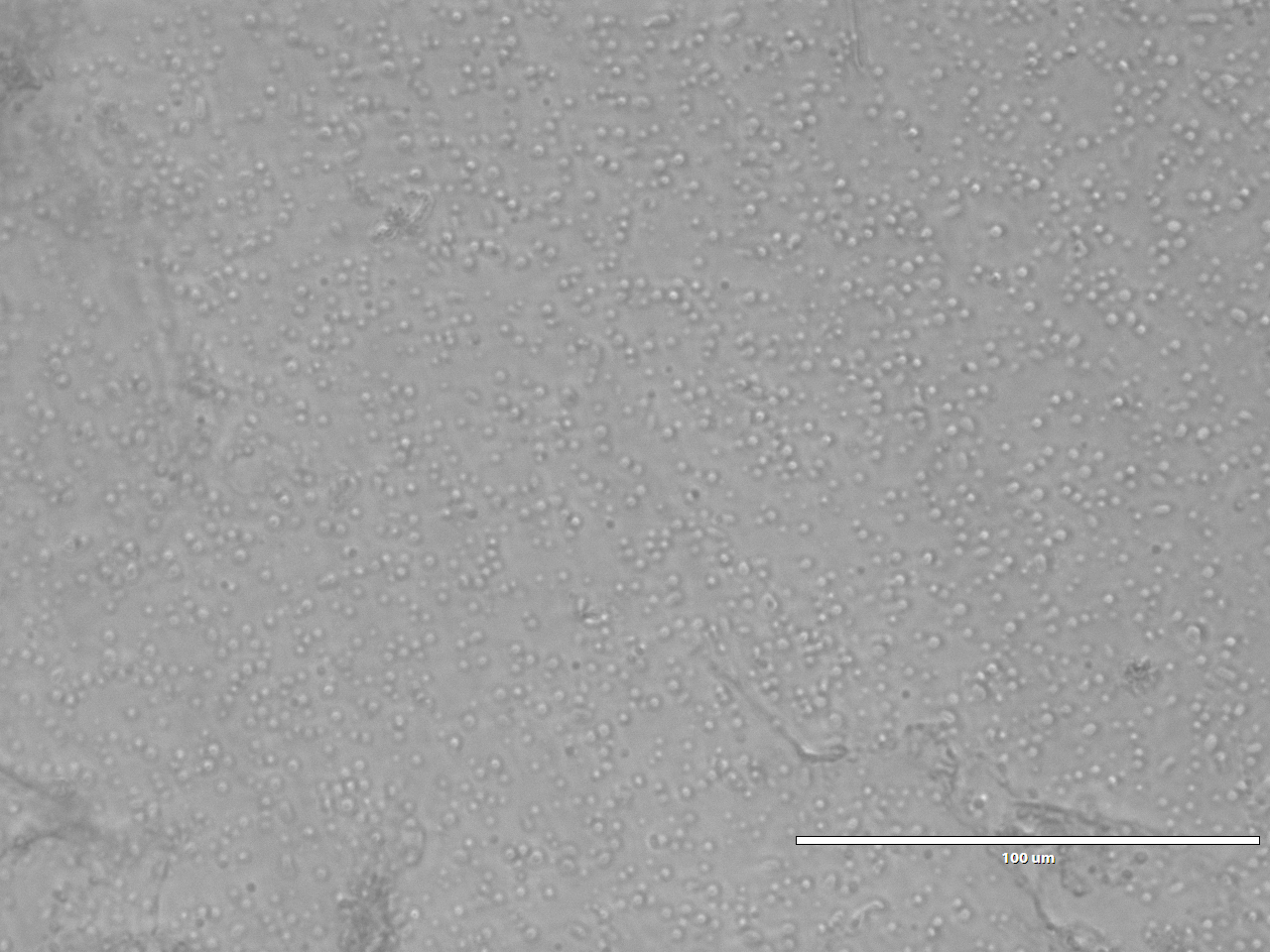

Supplement: Supplementary file 9 — Source Data Fig. 2 [file 44318_2023_21_MOESM9_ESM.zip › Figure 2/Figure 2C/MBP-52K WT_10uM_No kinase_20 mins.tif]

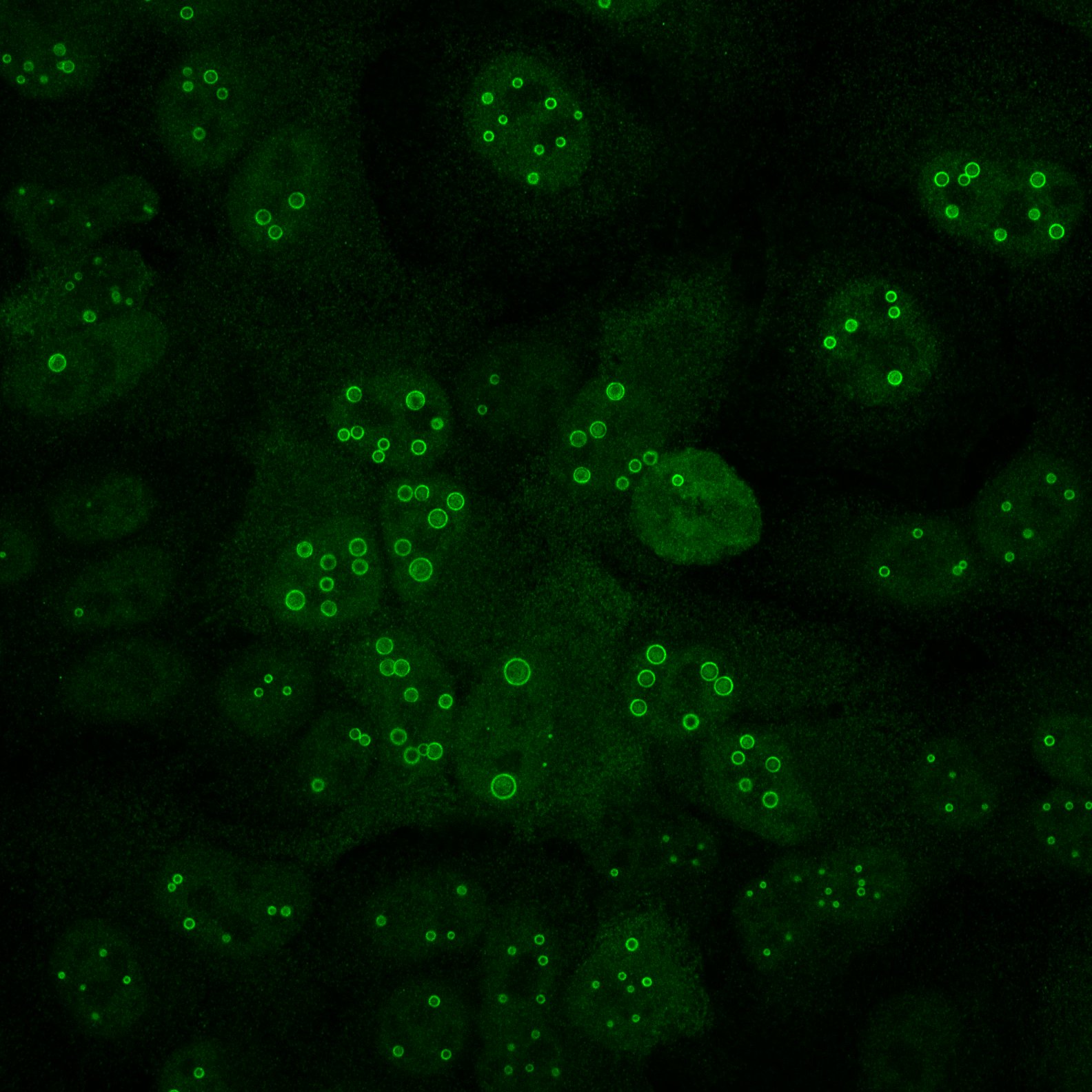

Supplement: Supplementary file 10 — Source Data Fig. 3 [file 44318_2023_21_MOESM10_ESM.zip › Figure 3/Figure 3B/ZMAX_A549 52K S_A_Dox 24 hrs_52K Channel (488).tif]

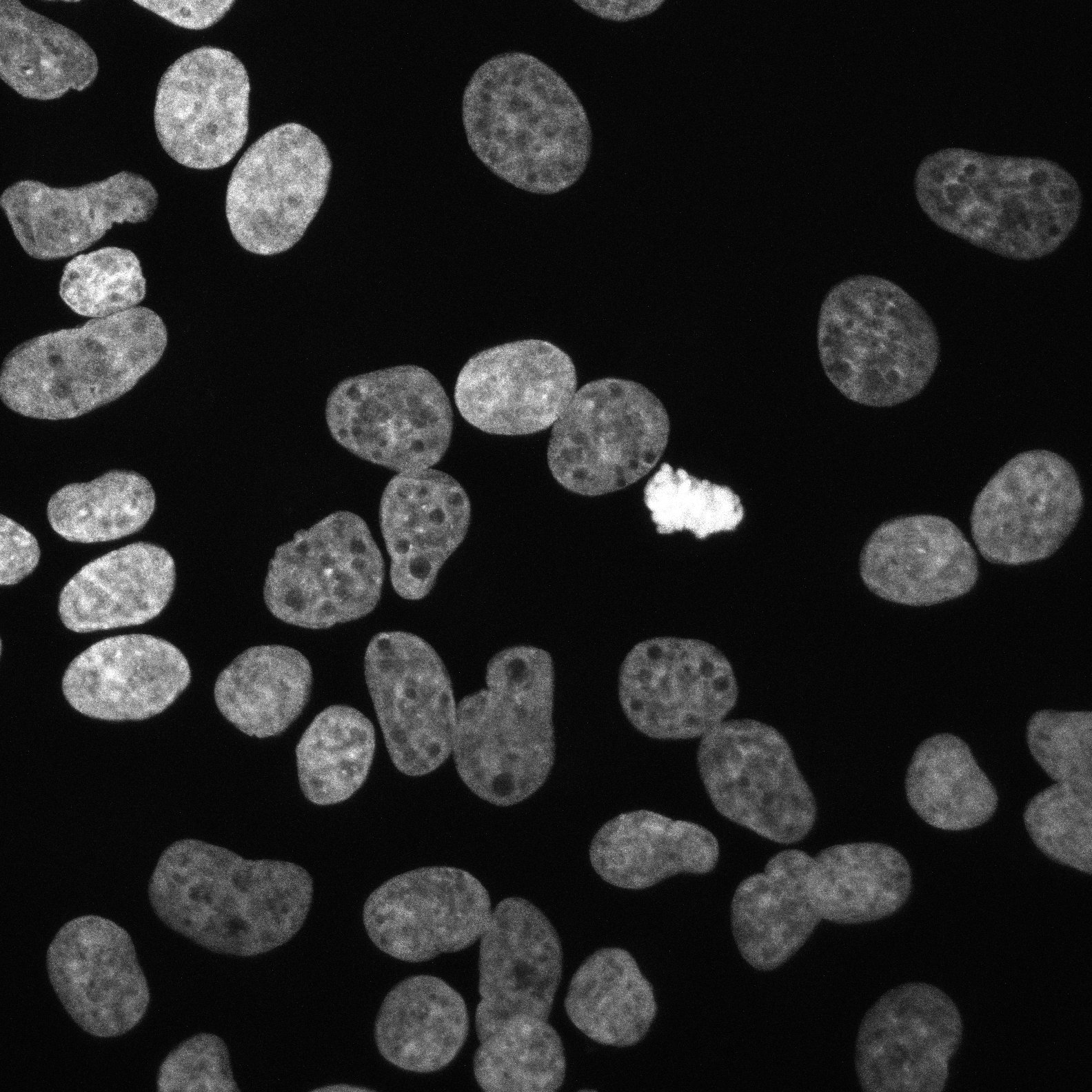

Supplement: Supplementary file 10 — Source Data Fig. 3 [file 44318_2023_21_MOESM10_ESM.zip › Figure 3/Figure 3B/ZMAX_A549 52K S_A_Dox 24 hrs_DAPI Channel (405).tif]

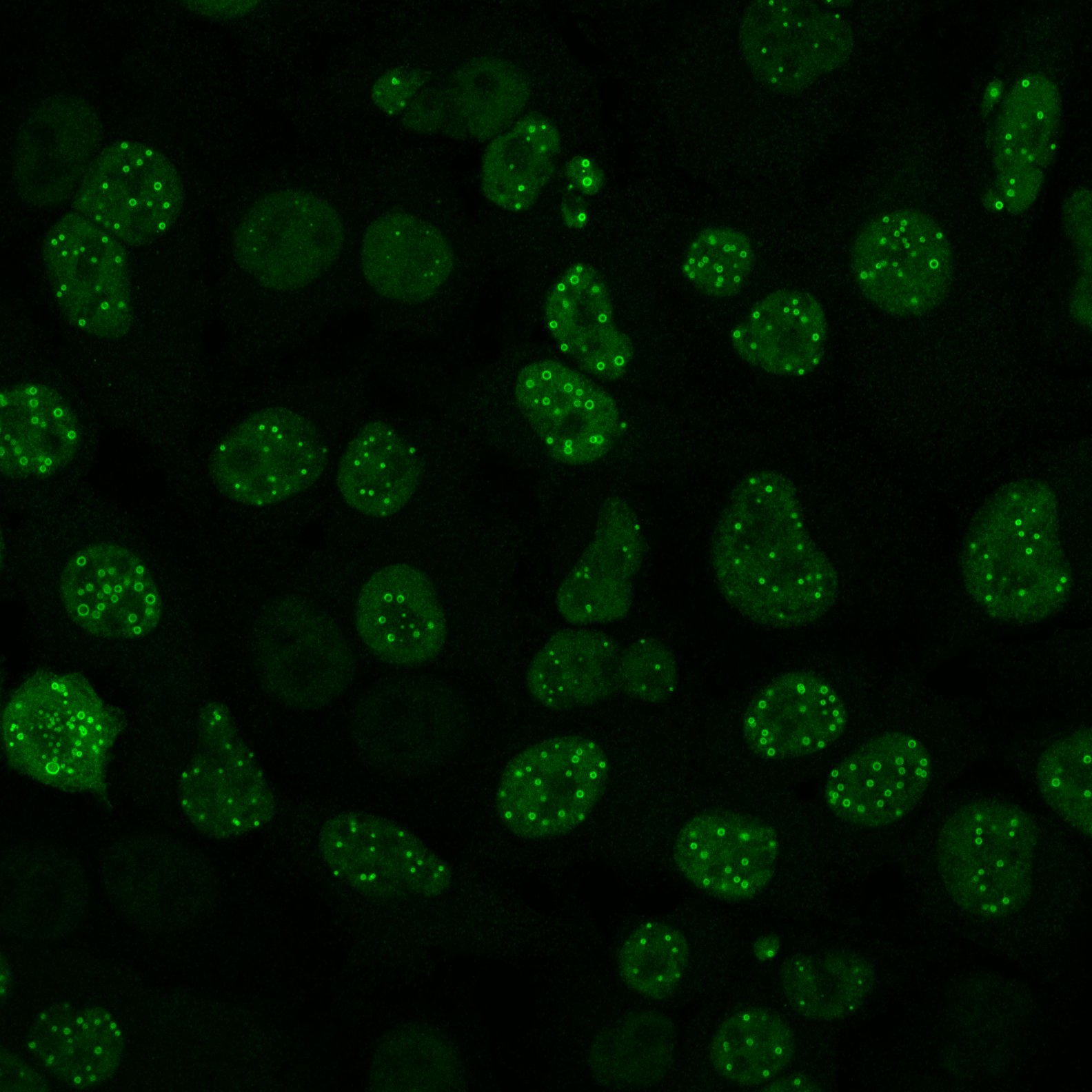

Supplement: Supplementary file 10 — Source Data Fig. 3 [file 44318_2023_21_MOESM10_ESM.zip › Figure 3/Figure 3B/ZMAX_A549 52K WT Dox 24hrs_52K Channel (488).tif]

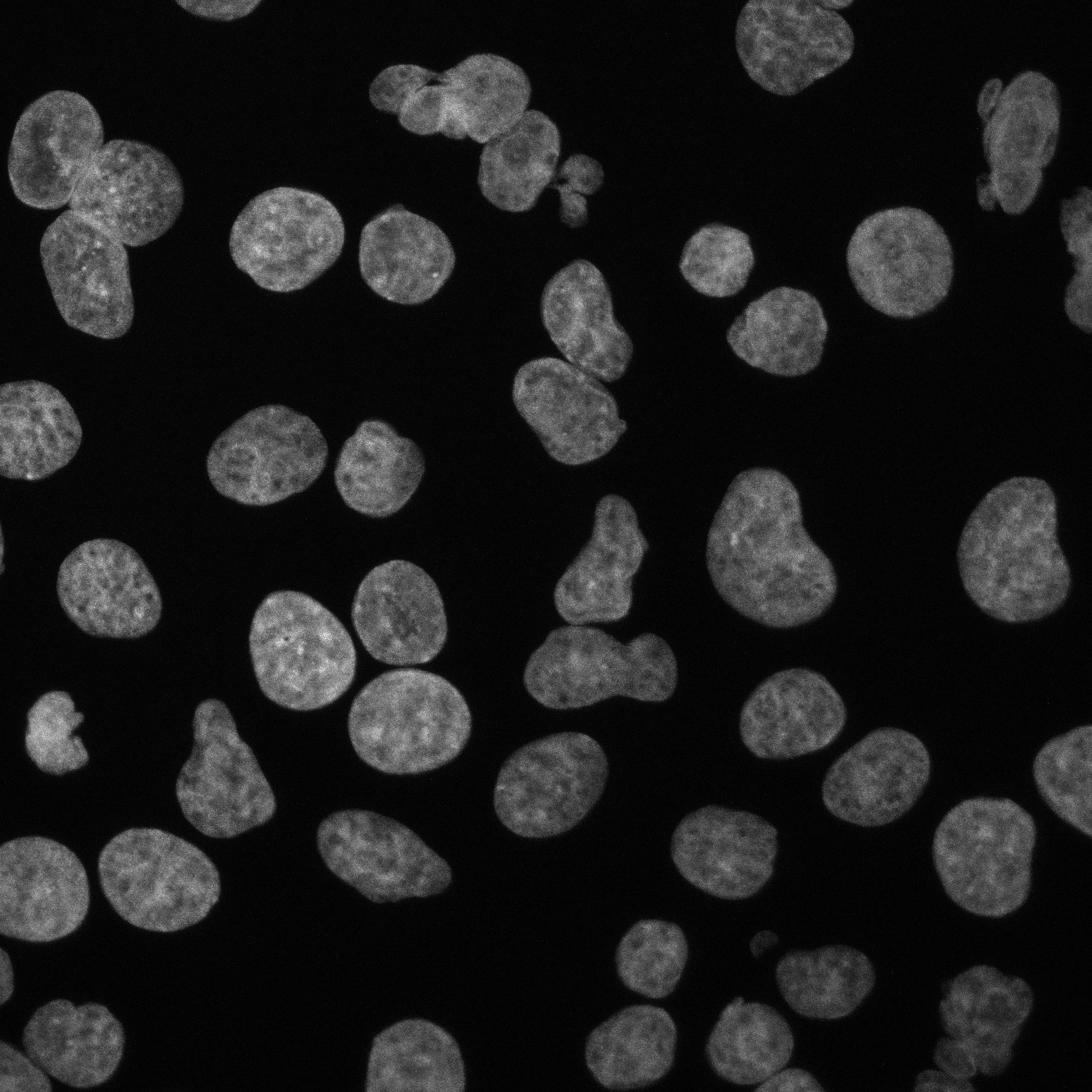

Supplement: Supplementary file 10 — Source Data Fig. 3 [file 44318_2023_21_MOESM10_ESM.zip › Figure 3/Figure 3B/ZMAX_A549 52K WT Dox 24hrs_DAPI Channel (405).tif]

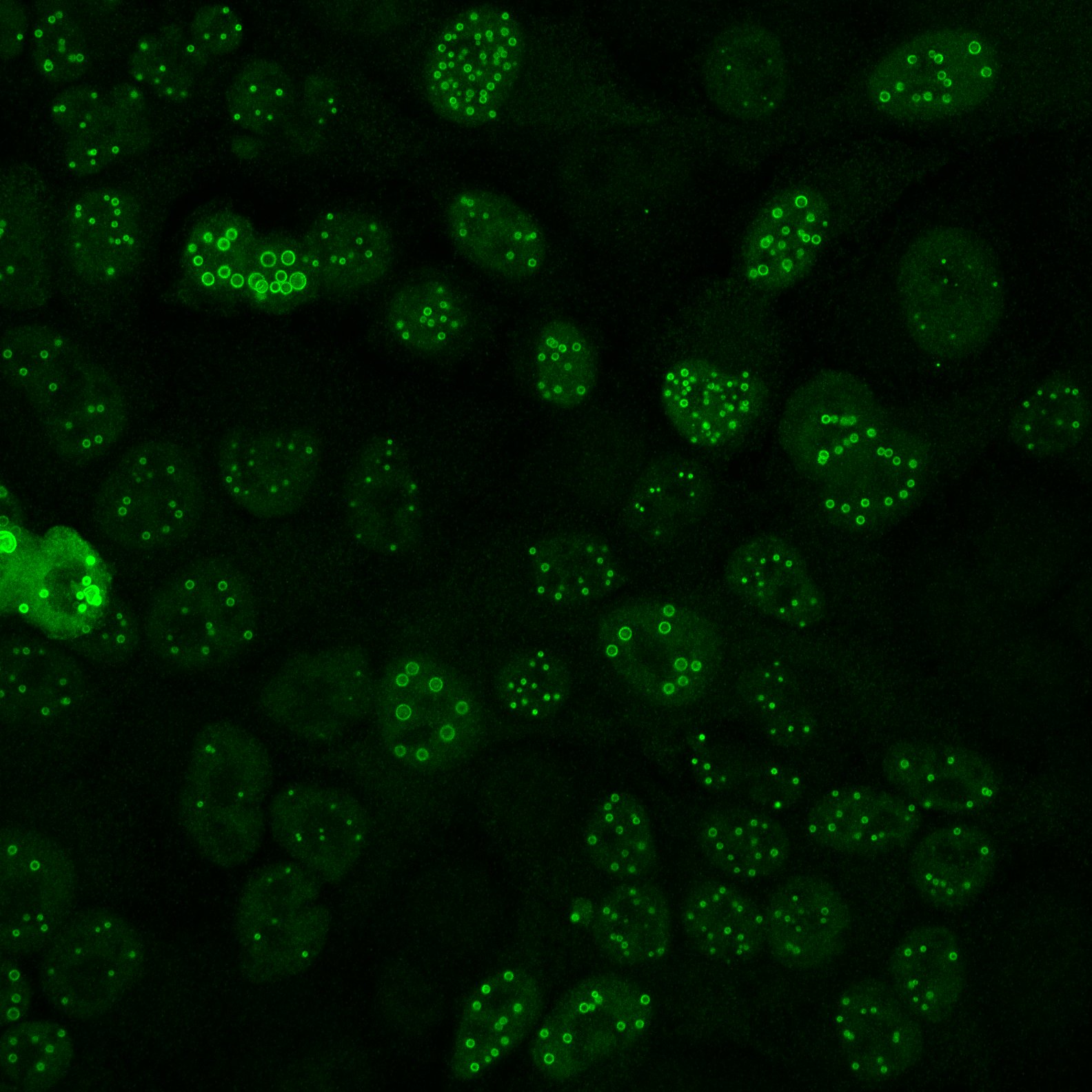

Supplement: Supplementary file 10 — Source Data Fig. 3 [file 44318_2023_21_MOESM10_ESM.zip › Figure 3/Figure 3B/ZMAX_A549 S_D_Dox 24hrs_52K Channel (488).tif]

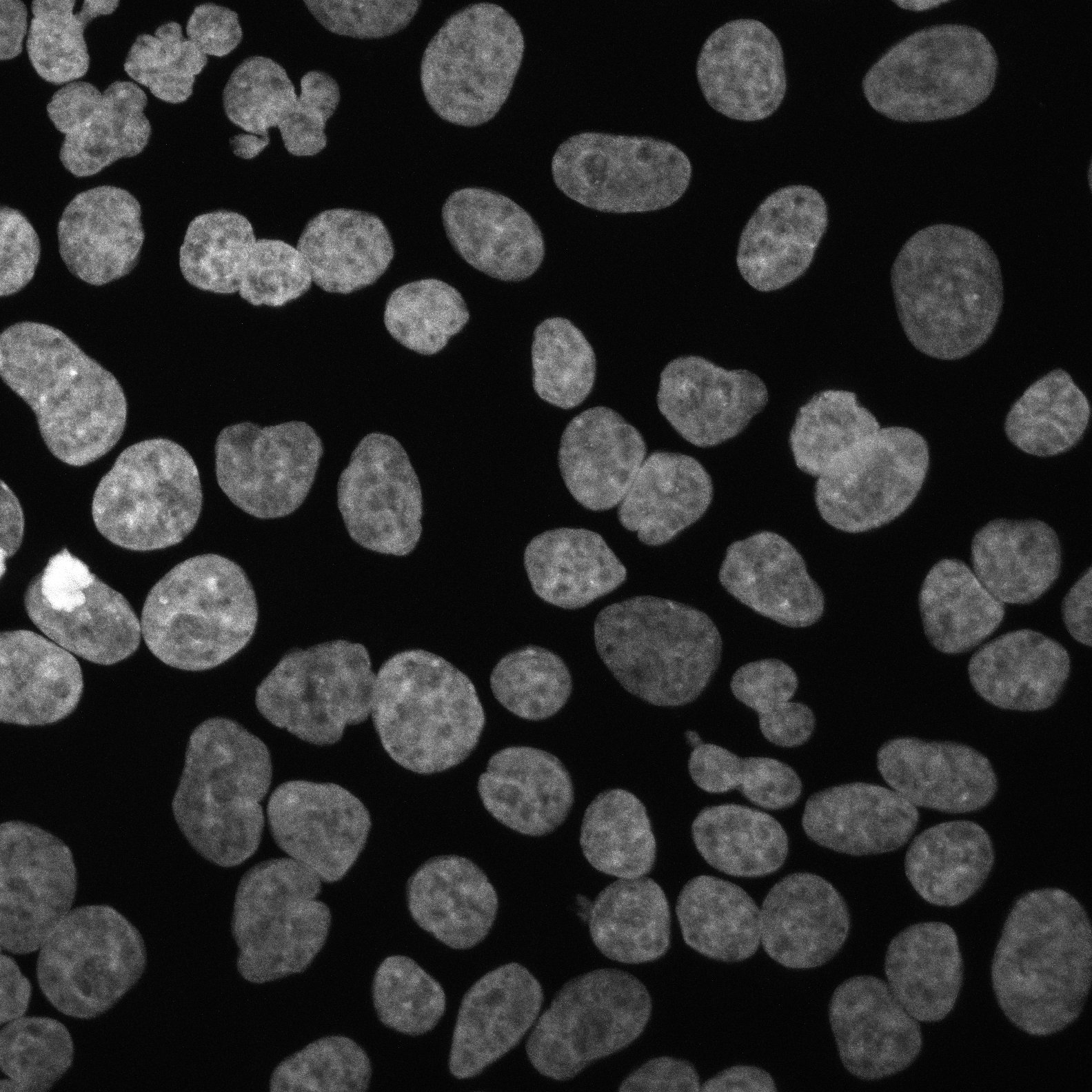

Supplement: Supplementary file 10 — Source Data Fig. 3 [file 44318_2023_21_MOESM10_ESM.zip › Figure 3/Figure 3B/ZMAX_A549 S_D_Dox 24hrs_DAPI Channel (405).tif]

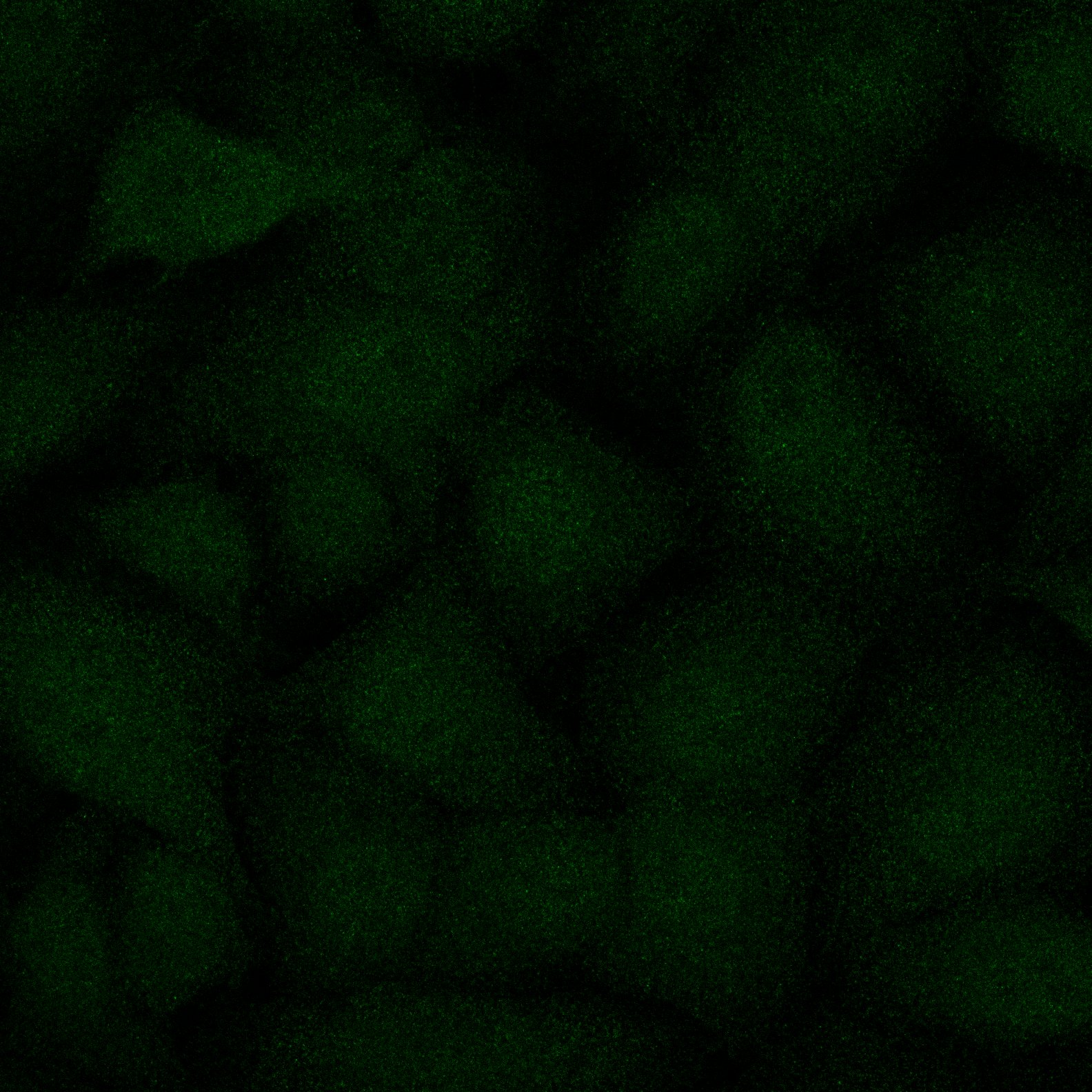

Supplement: Supplementary file 10 — Source Data Fig. 3 [file 44318_2023_21_MOESM10_ESM.zip › Figure 3/Figure 3B/ZMAX-A549 Parent_Dox 24 hrs_52K channel (488).tif]

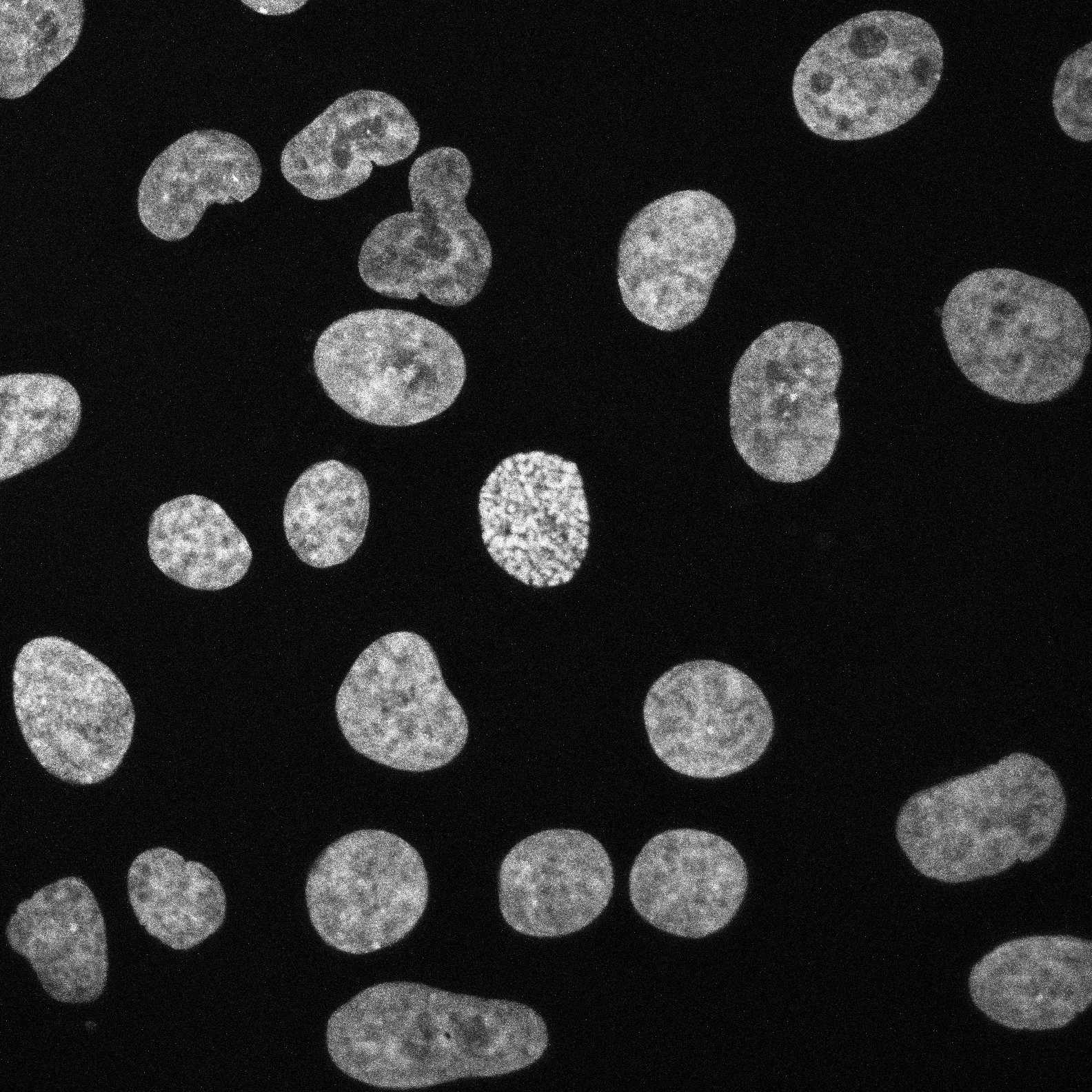

Supplement: Supplementary file 10 — Source Data Fig. 3 [file 44318_2023_21_MOESM10_ESM.zip › Figure 3/Figure 3B/ZMAX-A549 Parent_Dox 24 hrs_DAPI channel (405).tif]

# Figure 3: Panel F

Visible Composite

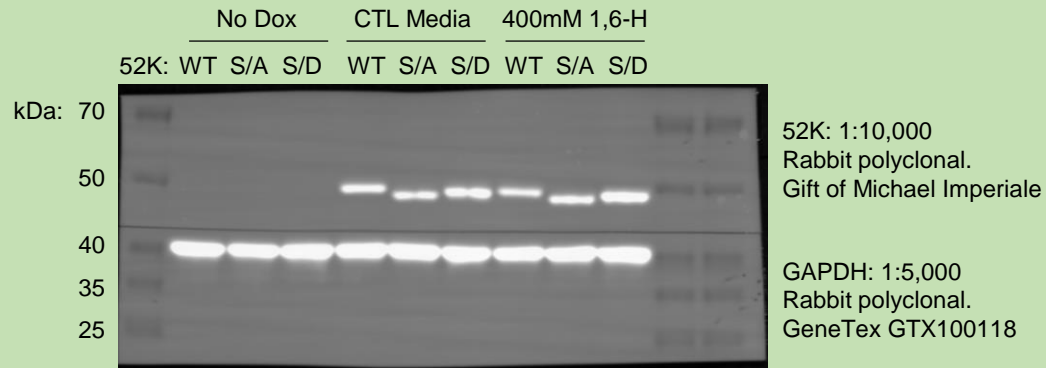

1 minute exposure

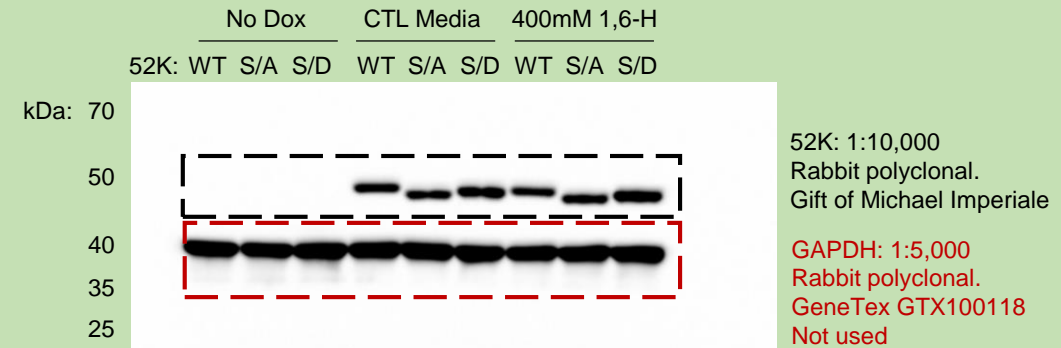

30 second exposure

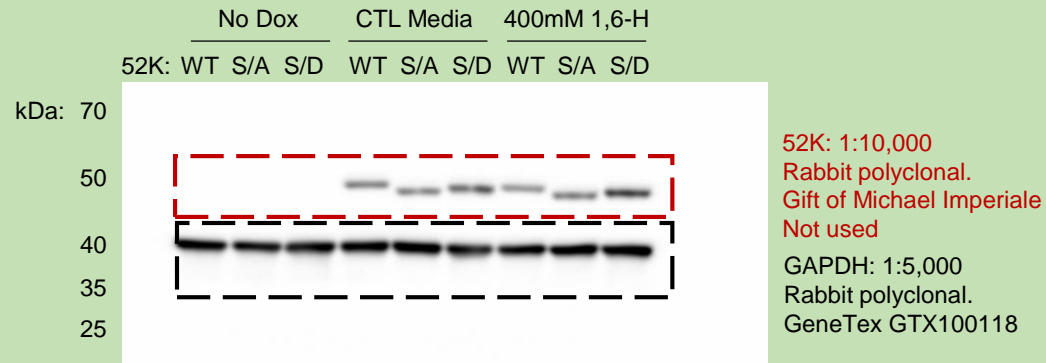

Supplement: Supplementary file 10 — Source Data Fig. 3 [file 44318_2023_21_MOESM10_ESM.zip › Figure 3/Figure 3F/Panel F_52K Expression in A549 52K Transgenic cells with 400mM Hex Treatment_Immunoblot.pdf]

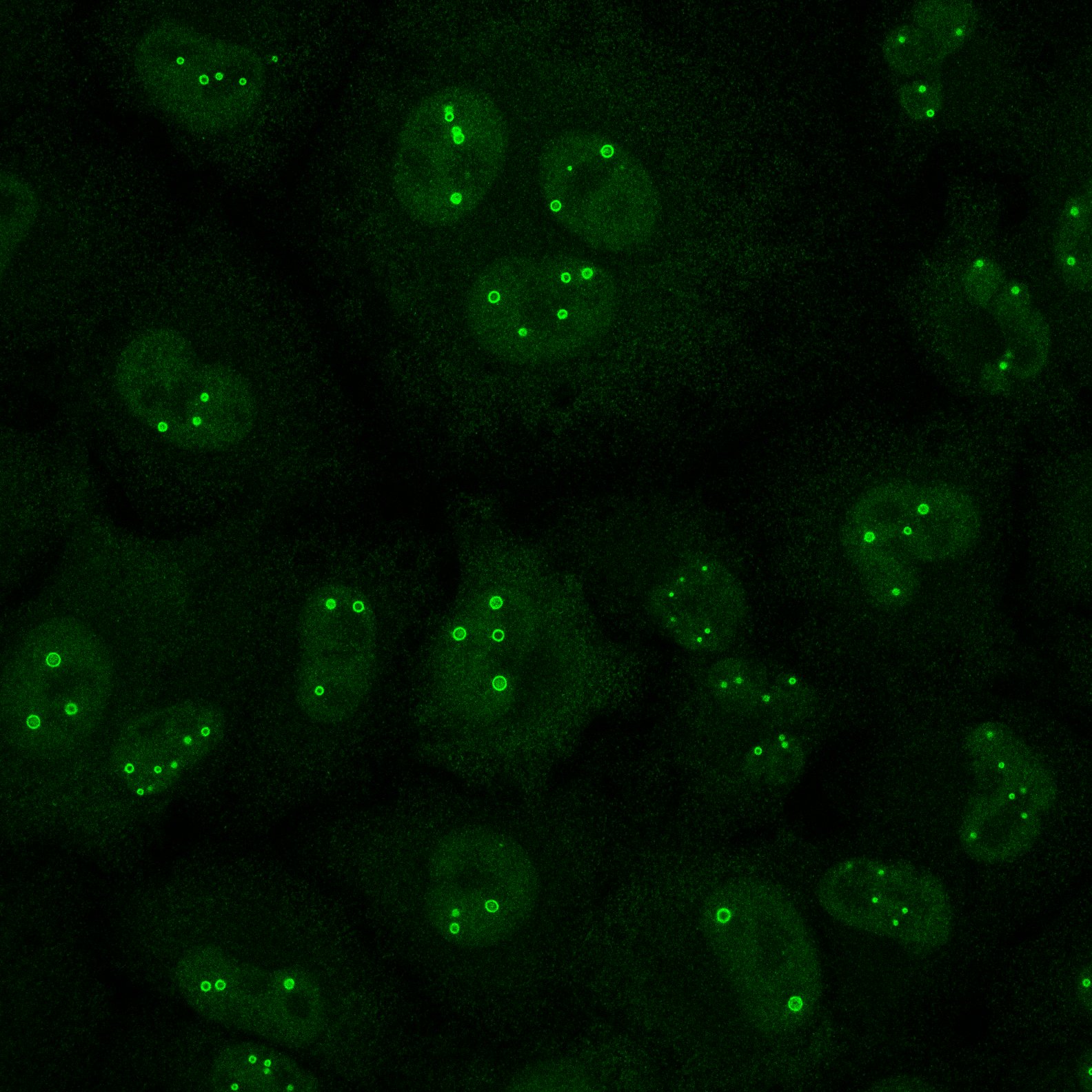

Supplement: Supplementary file 10 — Source Data Fig. 3 [file 44318_2023_21_MOESM10_ESM.zip › Figure 3/Figure 3G/ZMAX_MBP-52K S_A_10 hr dox control_52K Channel (488).tif]

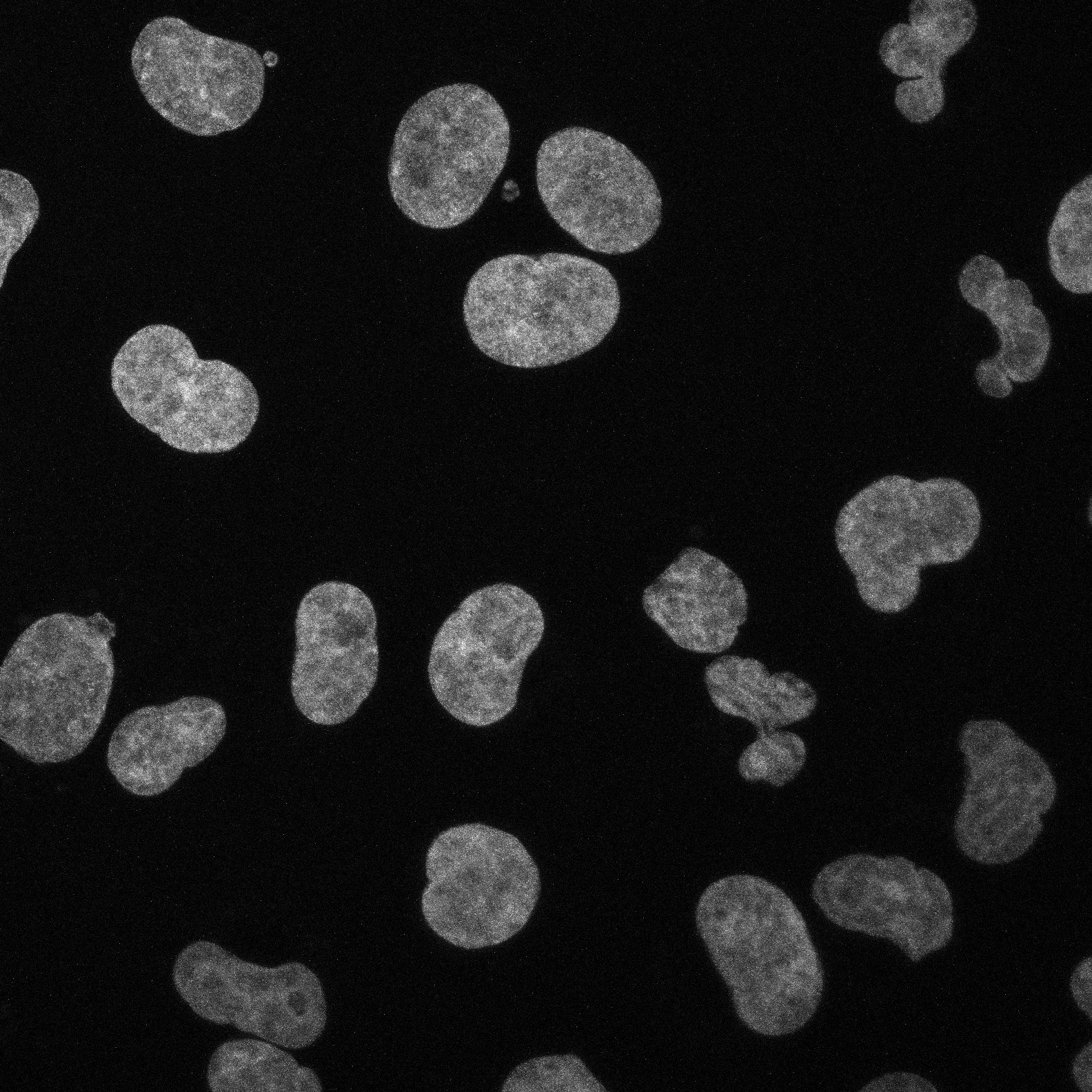

Supplement: Supplementary file 10 — Source Data Fig. 3 [file 44318_2023_21_MOESM10_ESM.zip › Figure 3/Figure 3G/ZMAX_MBP-52K S_A_10 hr dox control_DAPI Channel (405).tif]

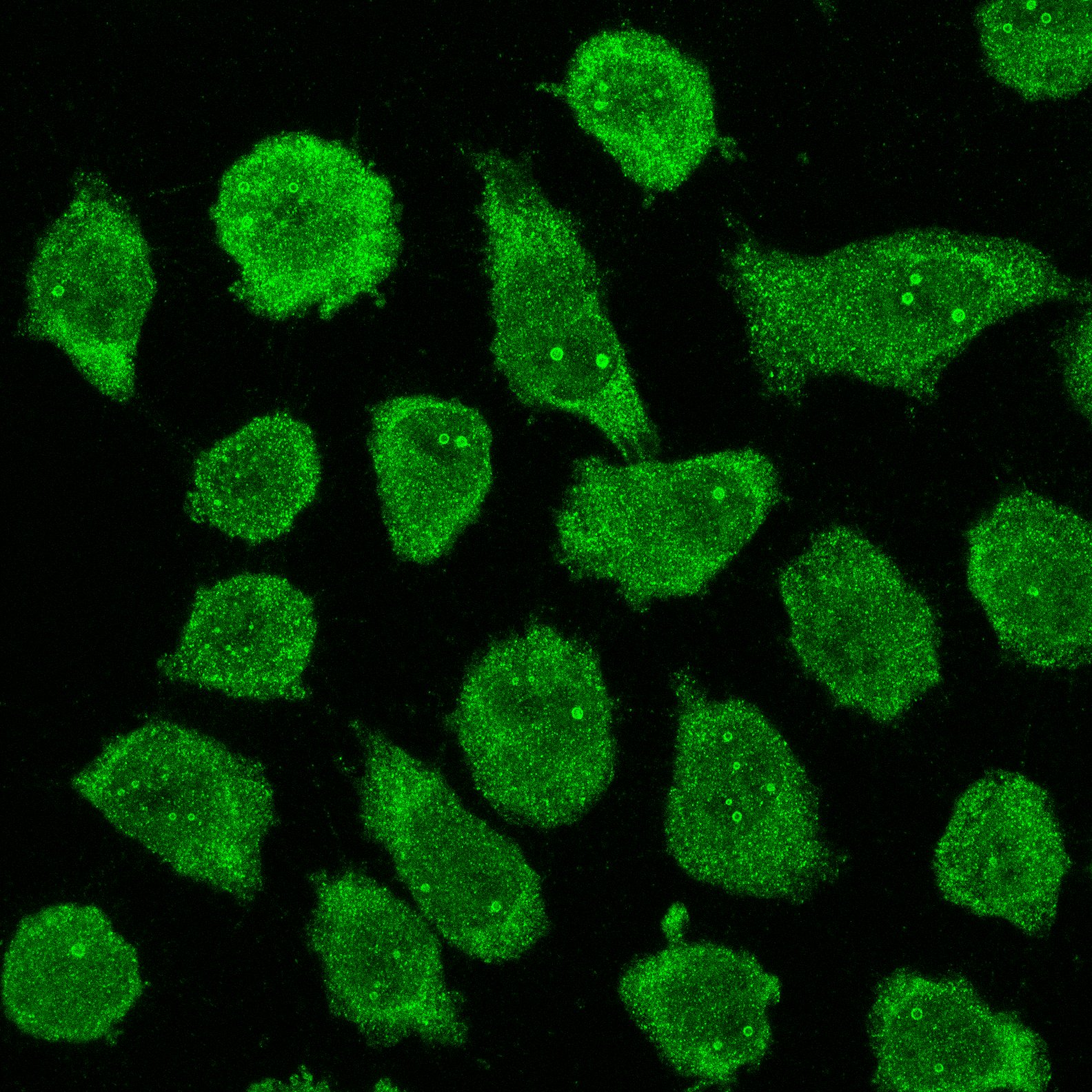

Supplement: Supplementary file 10 — Source Data Fig. 3 [file 44318_2023_21_MOESM10_ESM.zip › Figure 3/Figure 3G/ZMAX_MBP-52K S_A_400 mM Hex_52K Channel (488).tif]

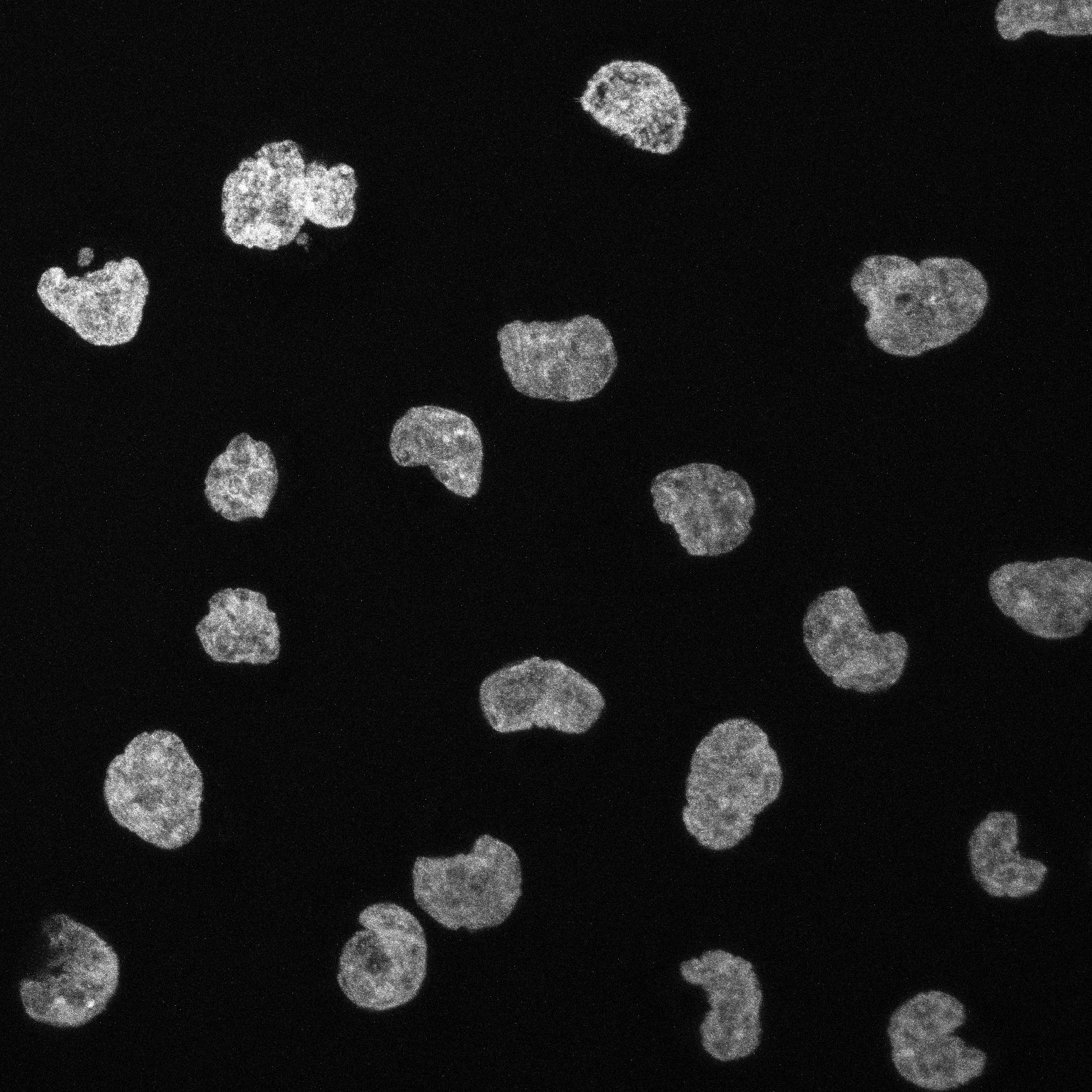

Supplement: Supplementary file 10 — Source Data Fig. 3 [file 44318_2023_21_MOESM10_ESM.zip › Figure 3/Figure 3G/ZMAX_MBP-52K S_A_400 mM Hex_DAPI Channel (405).tif]

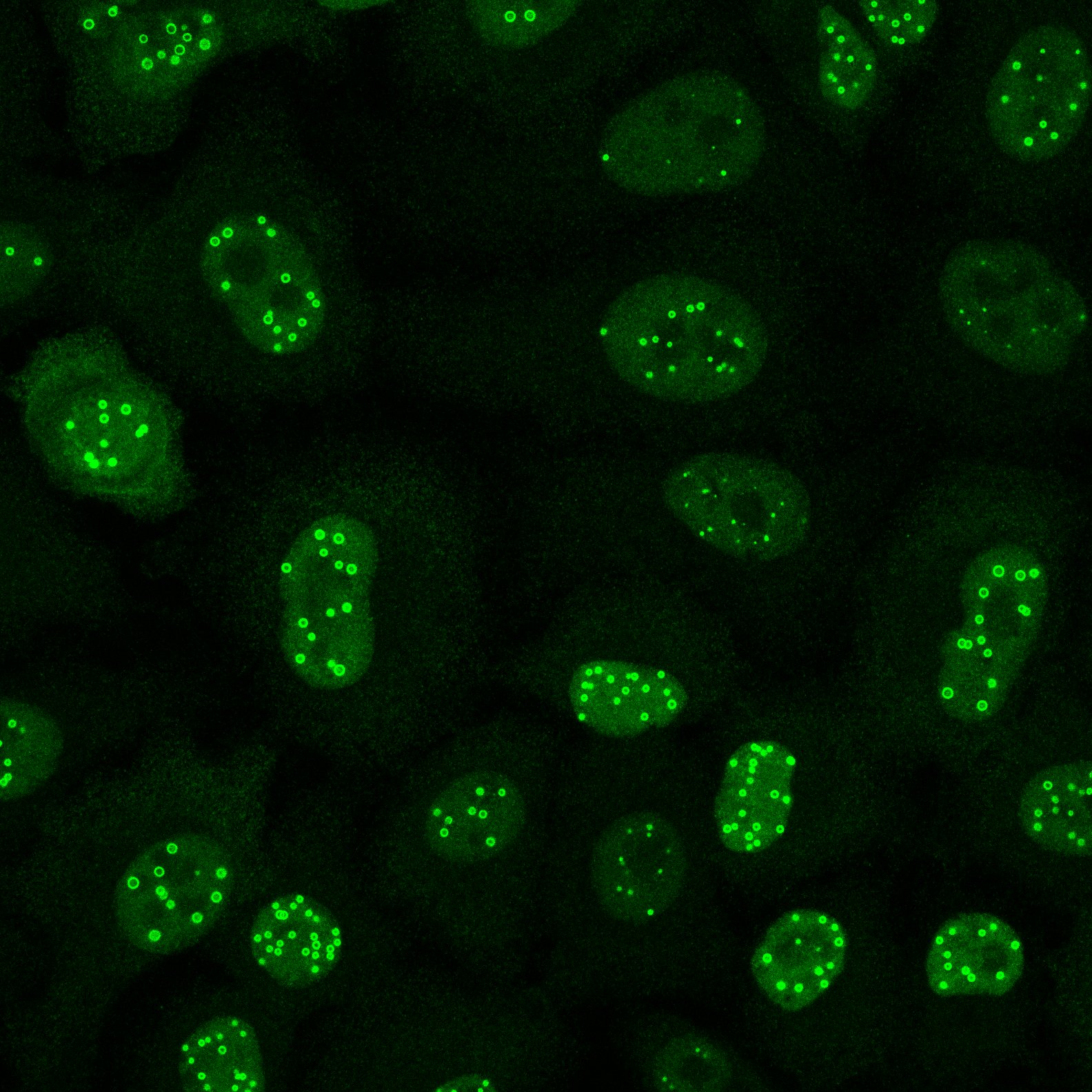

Supplement: Supplementary file 10 — Source Data Fig. 3 [file 44318_2023_21_MOESM10_ESM.zip › Figure 3/Figure 3G/ZMAX_MBP-52K S_D_10 hr dox control_52K Channel (488).tif]

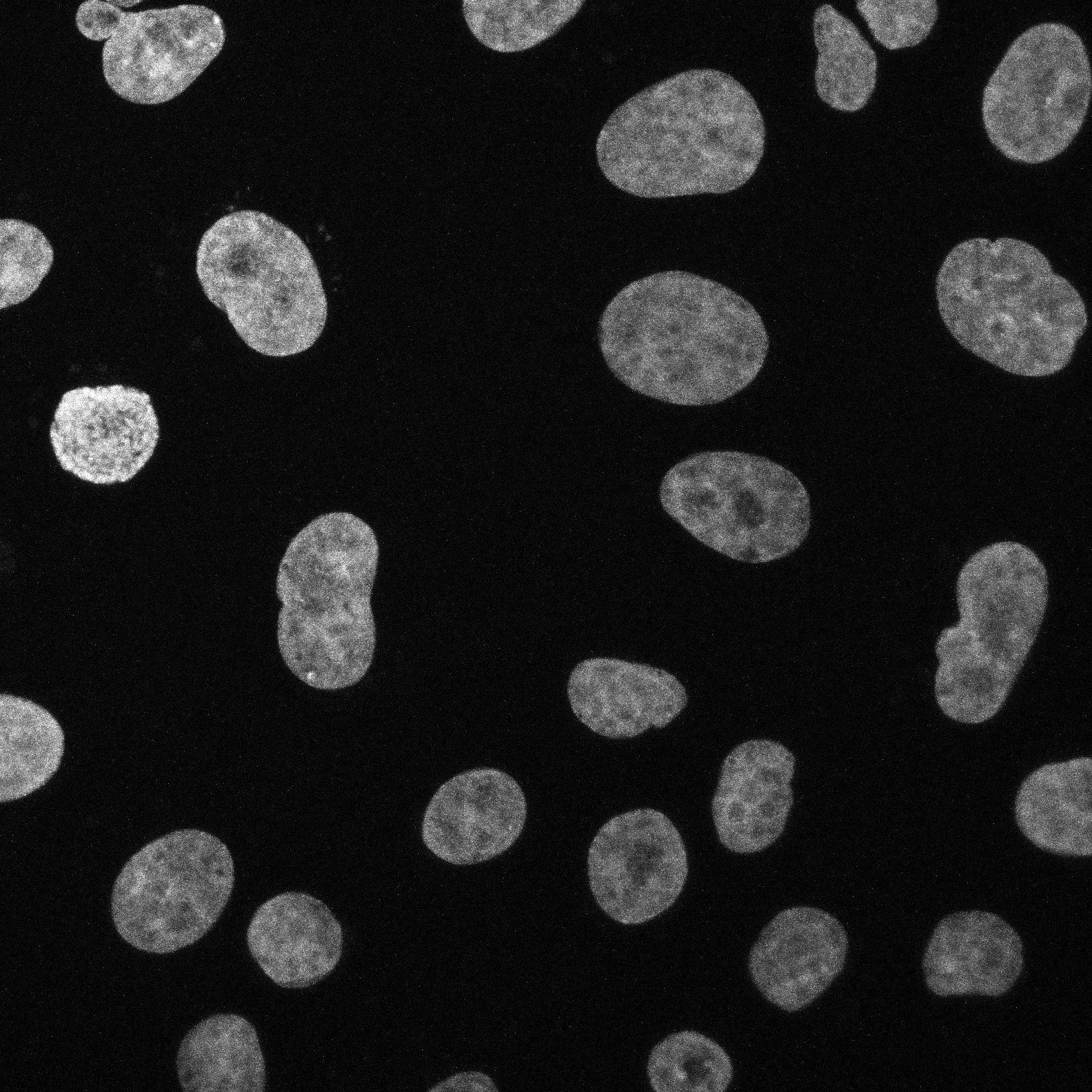

Supplement: Supplementary file 10 — Source Data Fig. 3 [file 44318_2023_21_MOESM10_ESM.zip › Figure 3/Figure 3G/ZMAX_MBP-52K S_D_10 hr dox control_DAPI Channel (405).tif]

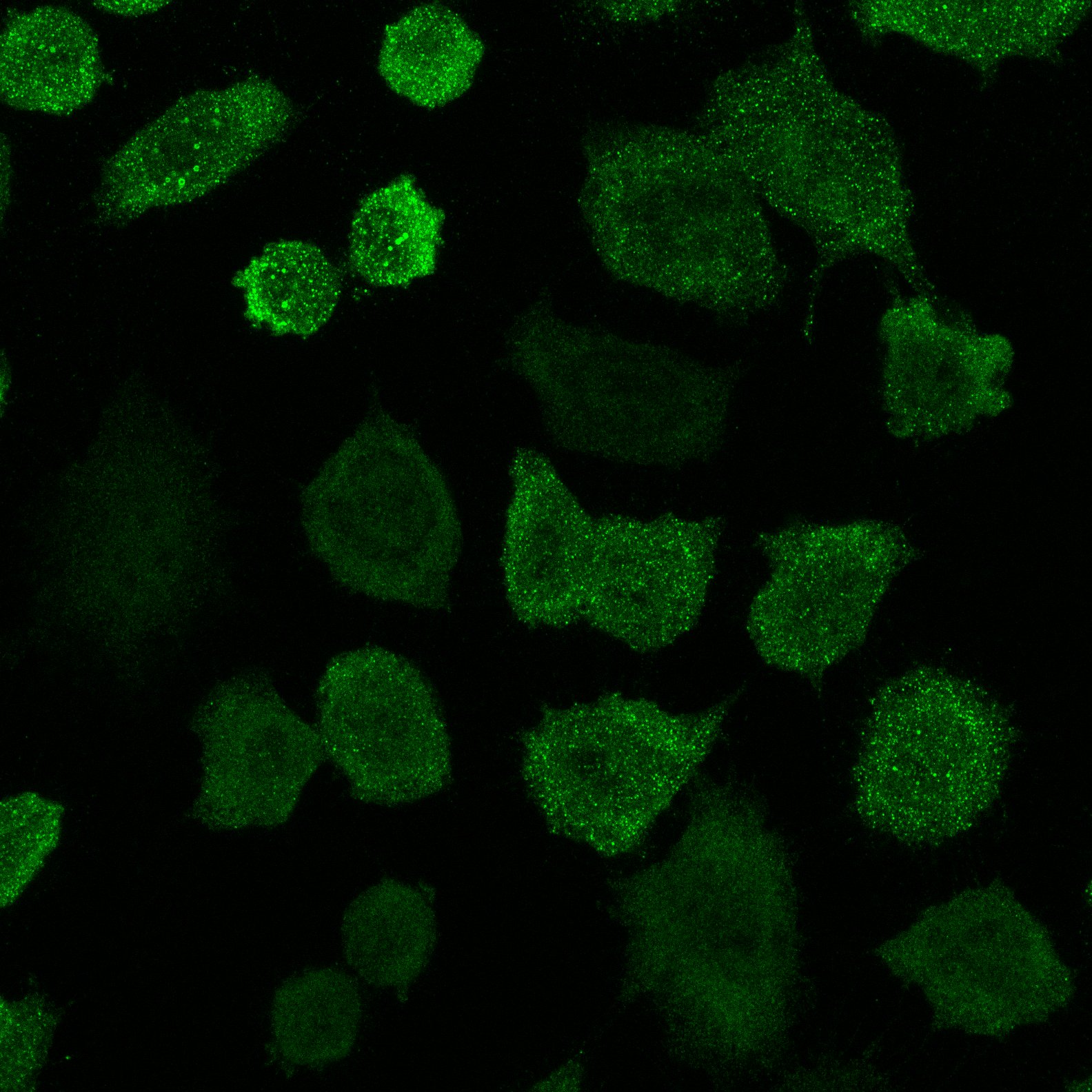

Supplement: Supplementary file 10 — Source Data Fig. 3 [file 44318_2023_21_MOESM10_ESM.zip › Figure 3/Figure 3G/ZMAX_MBP-52K S_D_400 mM Hex_52K Channel (488).tif]

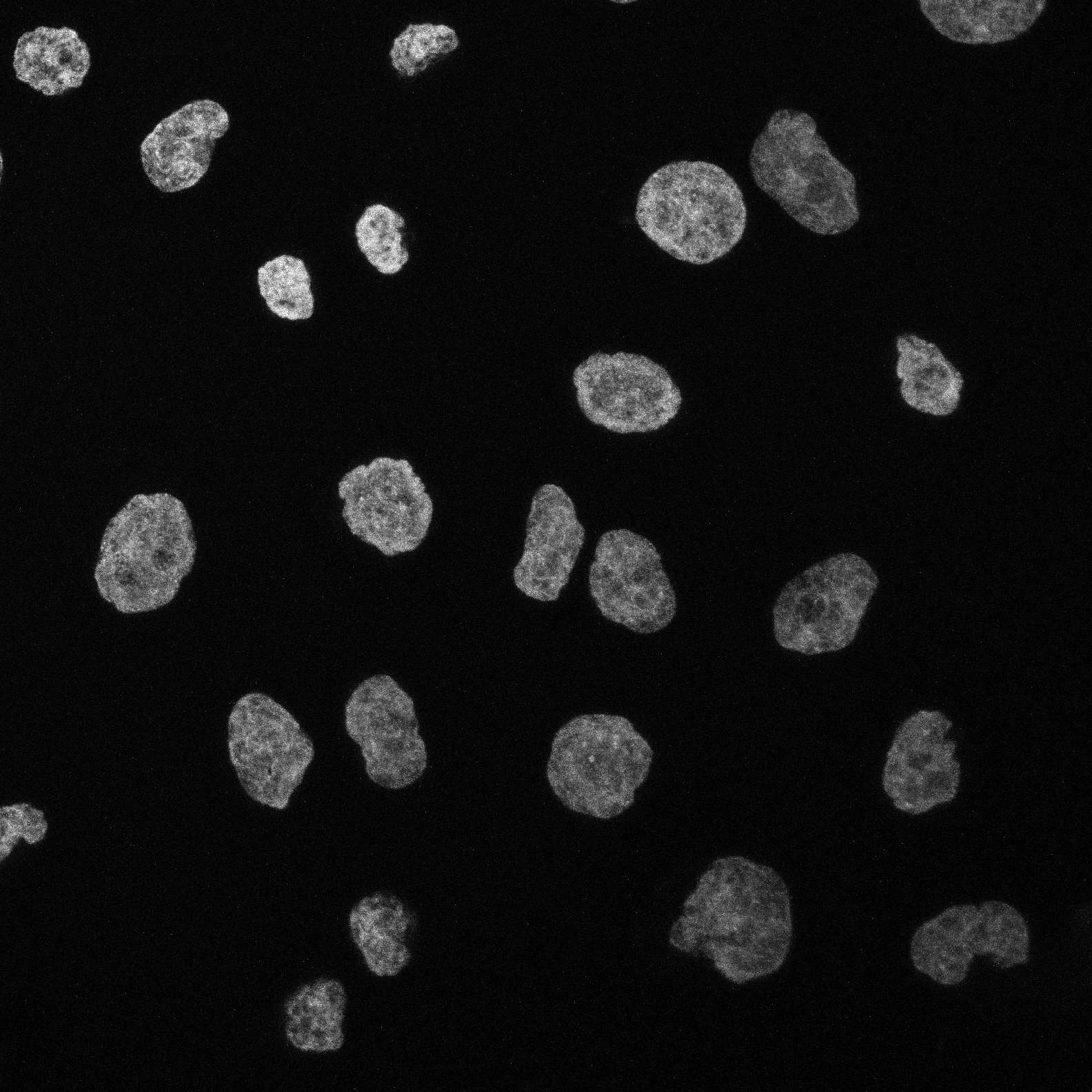

Supplement: Supplementary file 10 — Source Data Fig. 3 [file 44318_2023_21_MOESM10_ESM.zip › Figure 3/Figure 3G/ZMAX_MBP-52K S_D_400 mM Hex_DAPI Channel (405).tif]

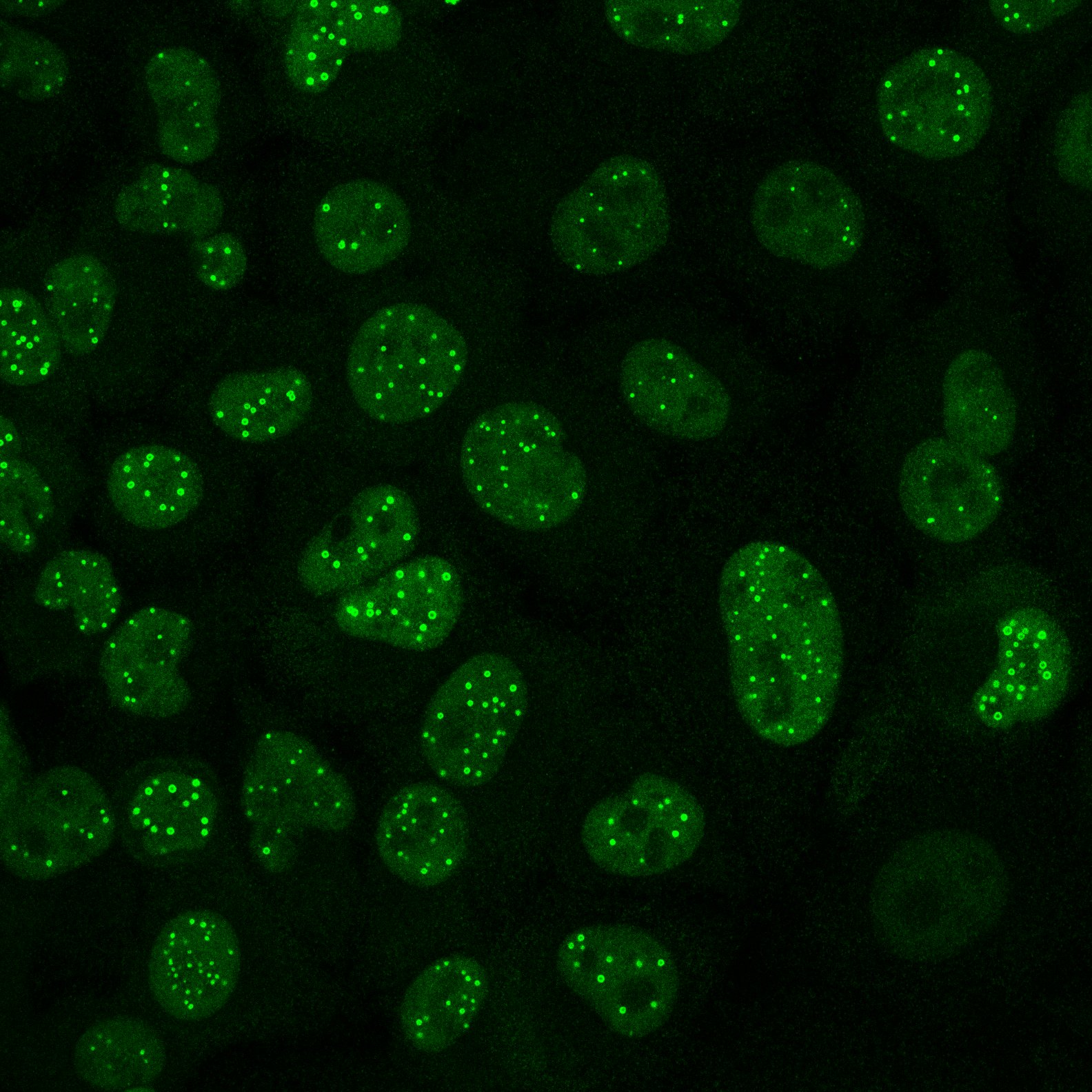

Supplement: Supplementary file 10 — Source Data Fig. 3 [file 44318_2023_21_MOESM10_ESM.zip › Figure 3/Figure 3G/ZMAX_MBP-52K WT_10 hr dox control_52K Channel (488).tif]

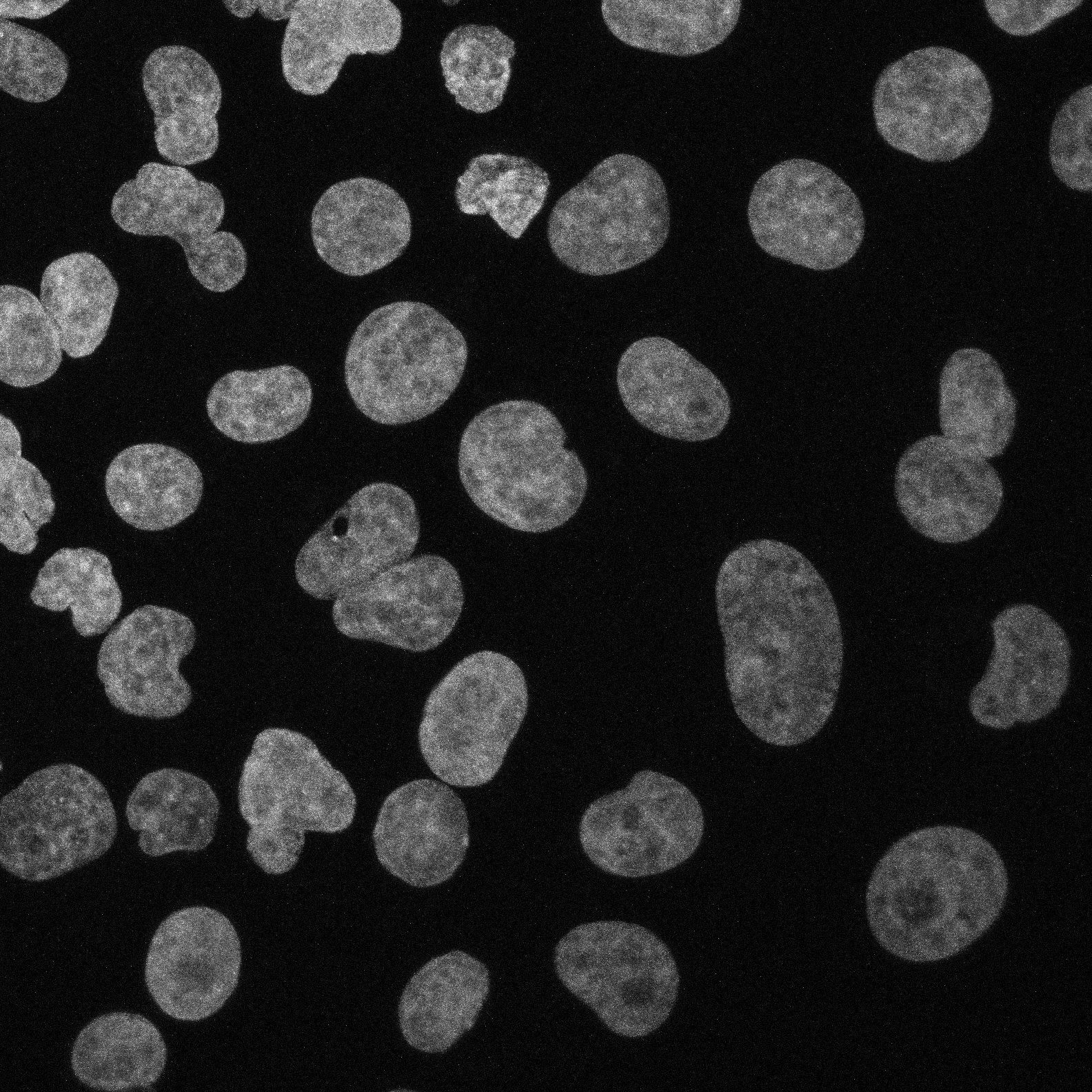

Supplement: Supplementary file 10 — Source Data Fig. 3 [file 44318_2023_21_MOESM10_ESM.zip › Figure 3/Figure 3G/ZMAX_MBP-52K WT_10 hr dox control_DAPI Channel (405).tif]

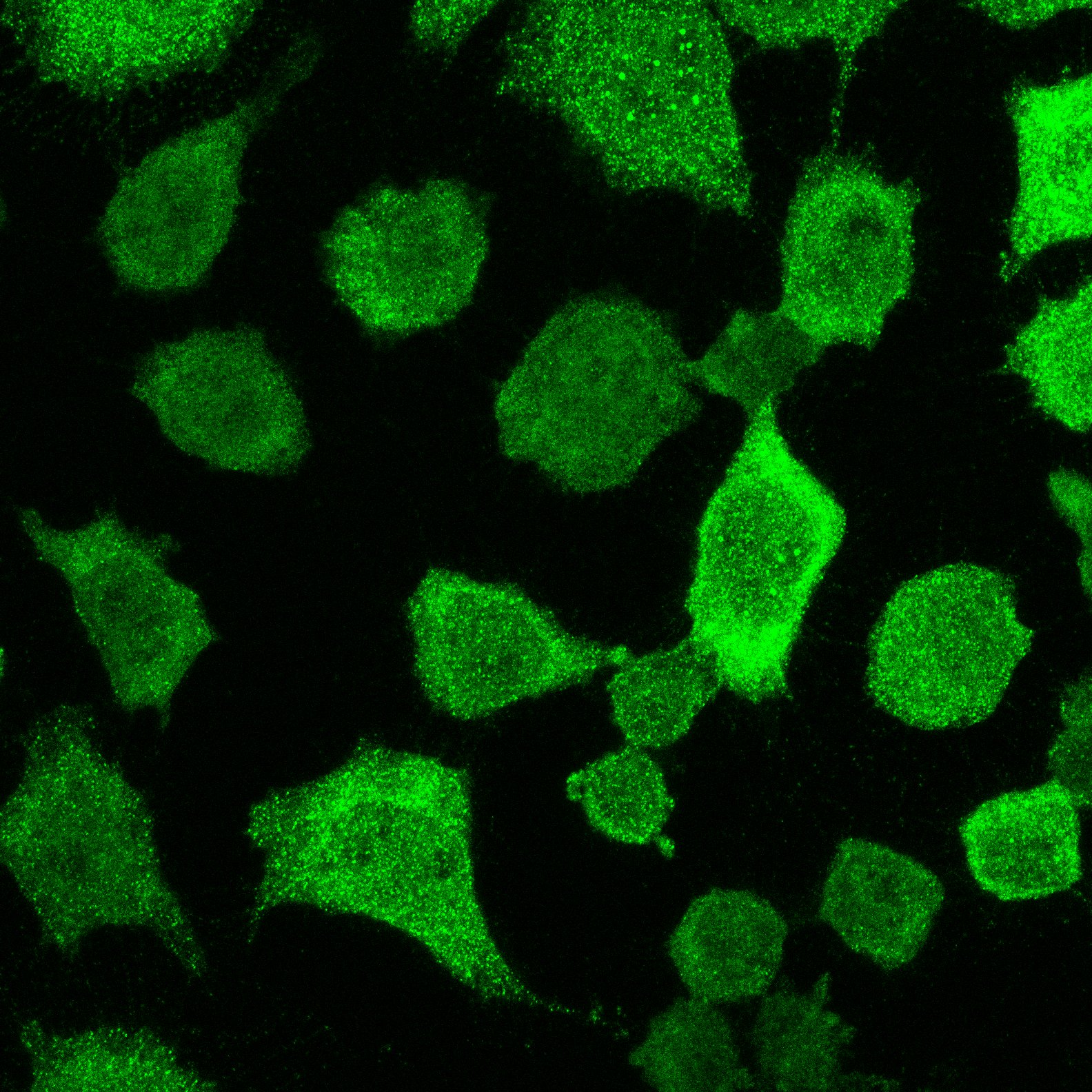

Supplement: Supplementary file 10 — Source Data Fig. 3 [file 44318_2023_21_MOESM10_ESM.zip › Figure 3/Figure 3G/ZMAX_MBP-52K WT_400 mM Hex_52K Channel (488).tif]

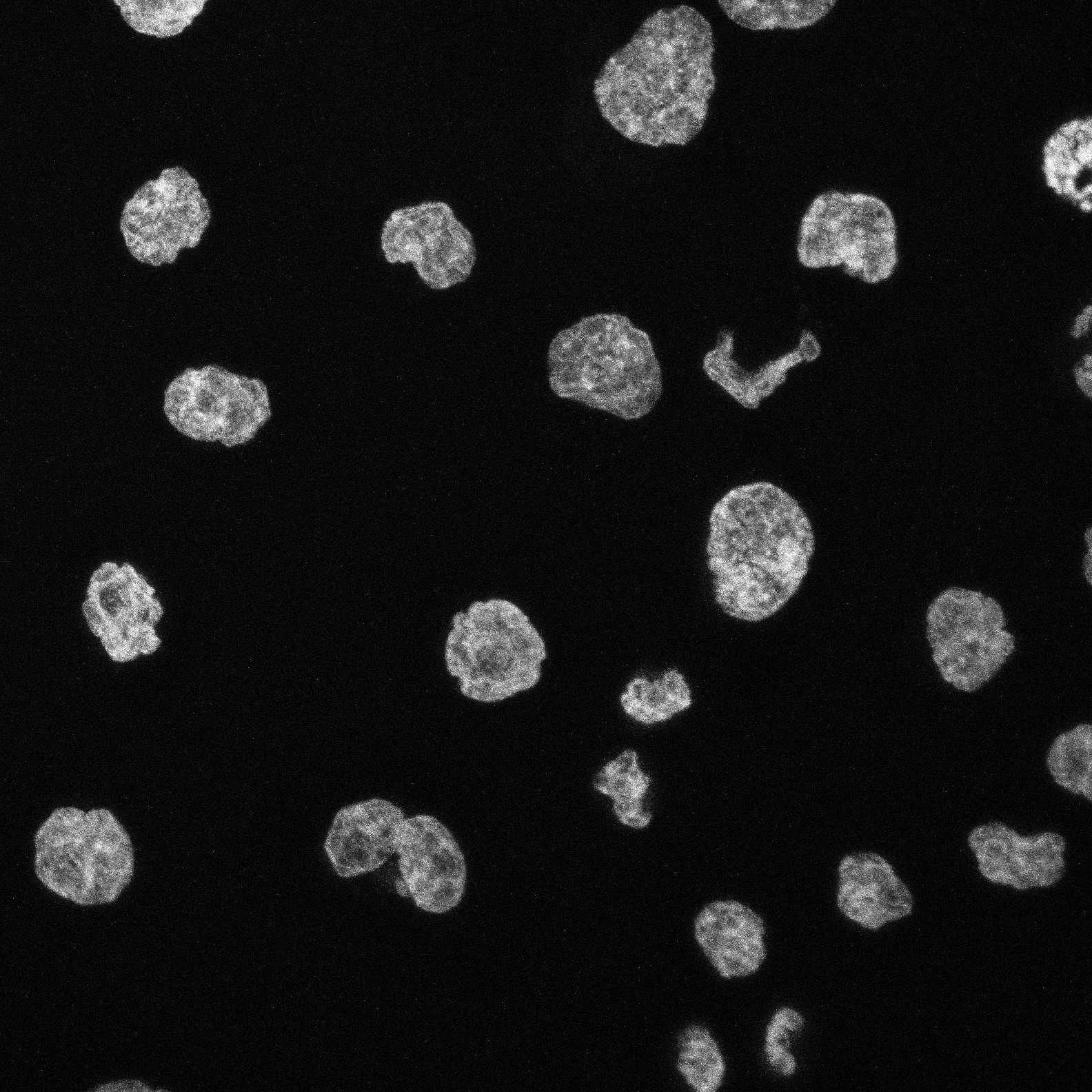

Supplement: Supplementary file 10 — Source Data Fig. 3 [file 44318_2023_21_MOESM10_ESM.zip › Figure 3/Figure 3G/ZMAX_MBP-52K WT_400 mM Hex_DAPI Channel (405).tif]

Figure 3: Panel I

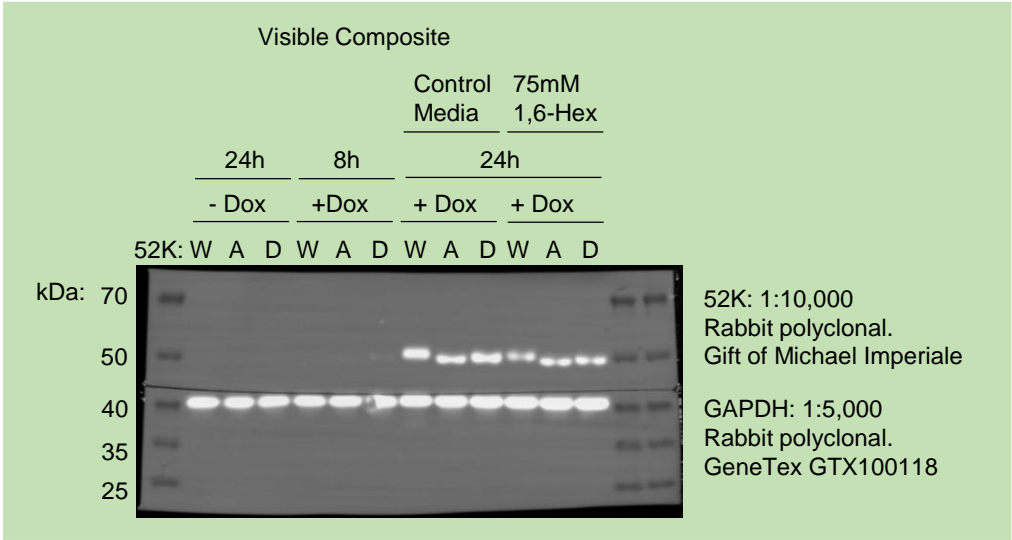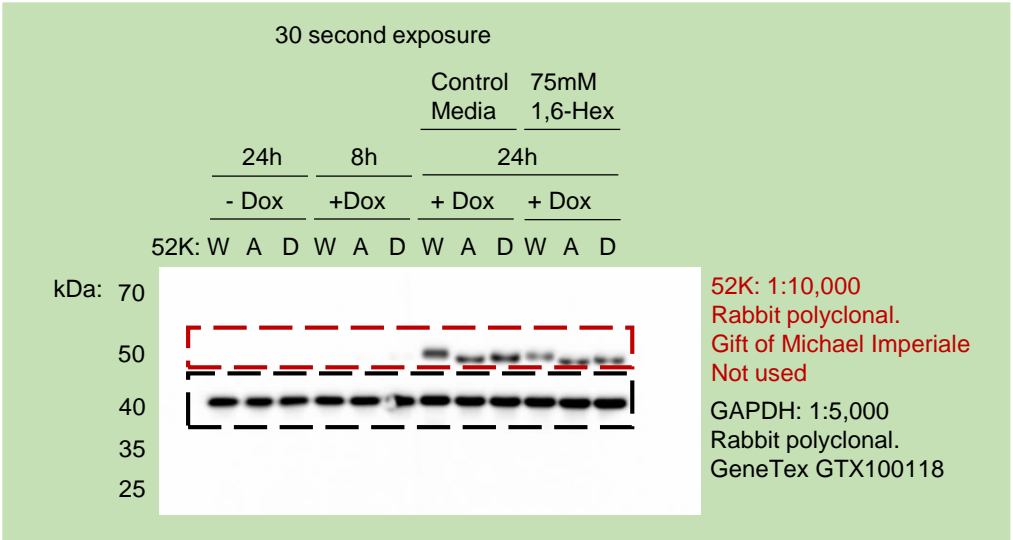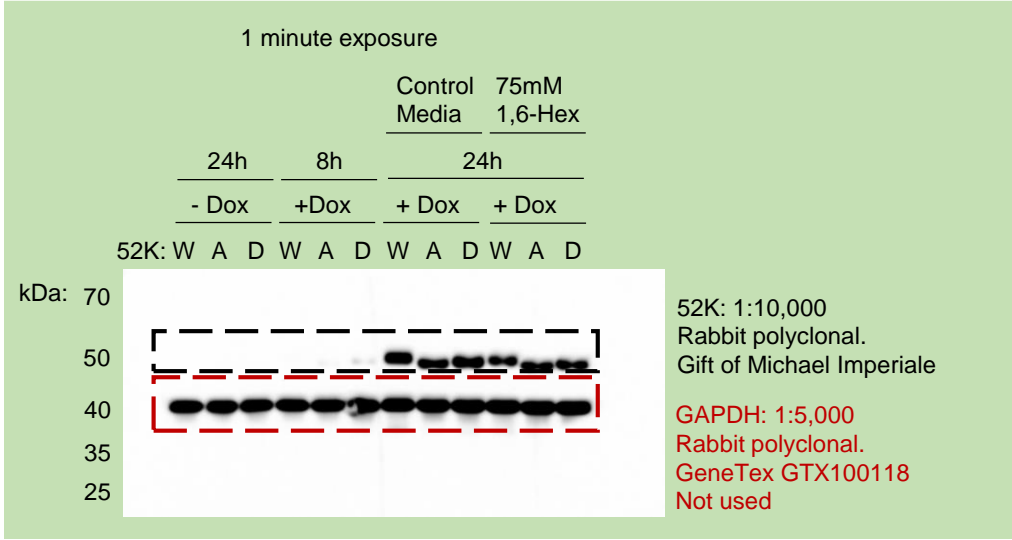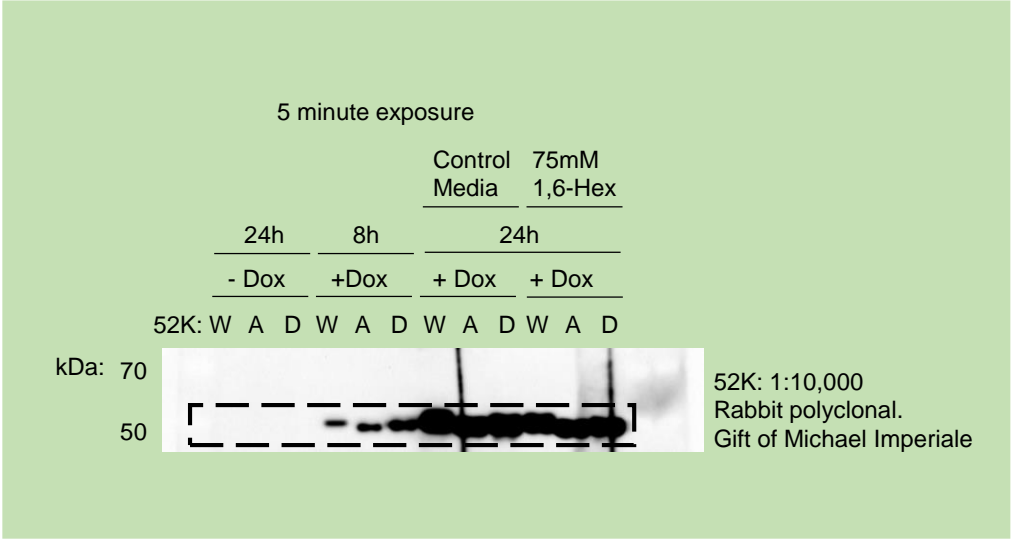

Supplement: Supplementary file 10 — Source Data Fig. 3 [file 44318_2023_21_MOESM10_ESM.zip › Figure 3/Figure 3I/Panel I_A549 52K Transgenic Cells under 75mM Hex Treatment_Immunoblot.pdf]

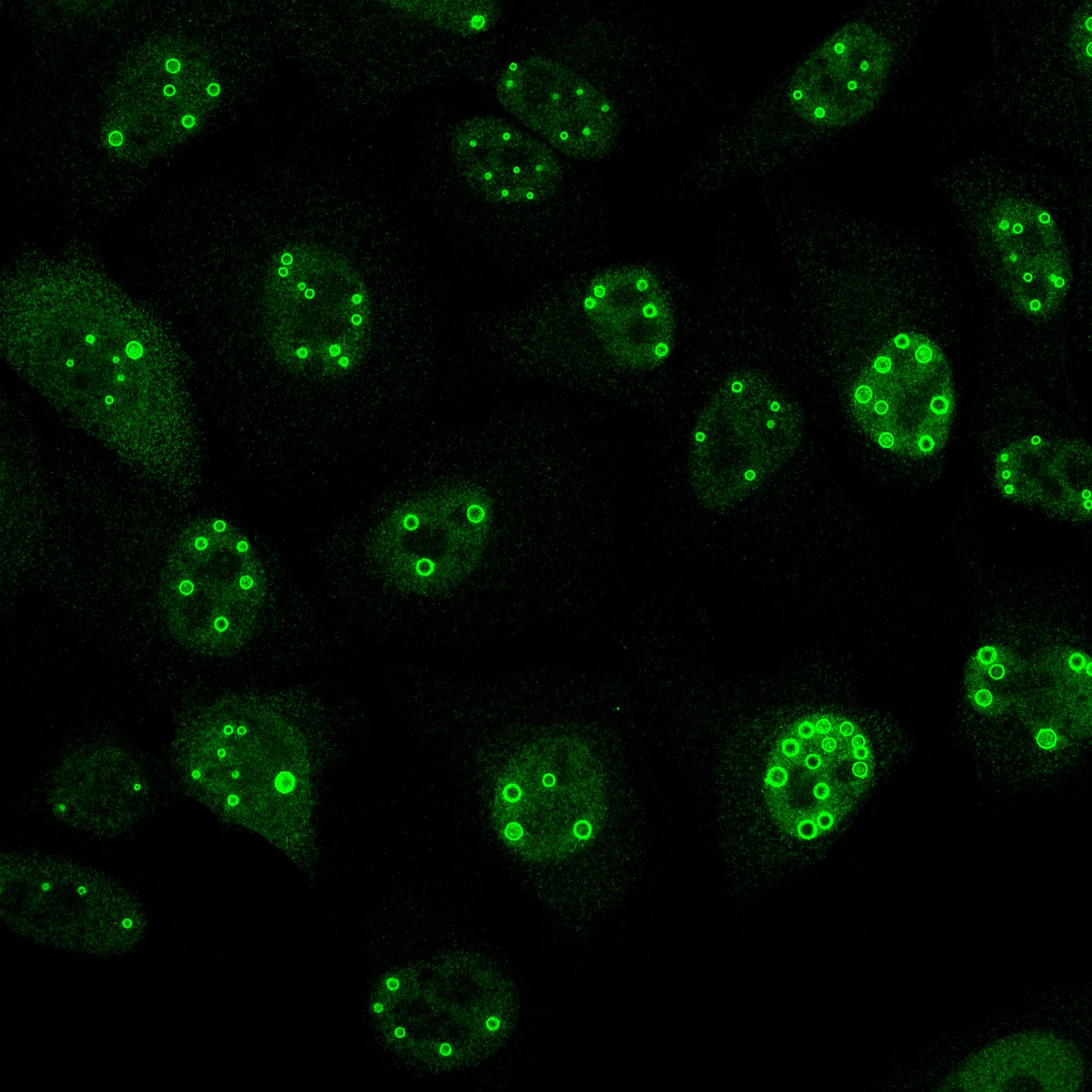

Supplement: Supplementary file 10 — Source Data Fig. 3 [file 44318_2023_21_MOESM10_ESM.zip › Figure 3/Figure 3J/ZMAX_A549 52K S_A_24 hr dox CTL _52K Channel (488).tif]

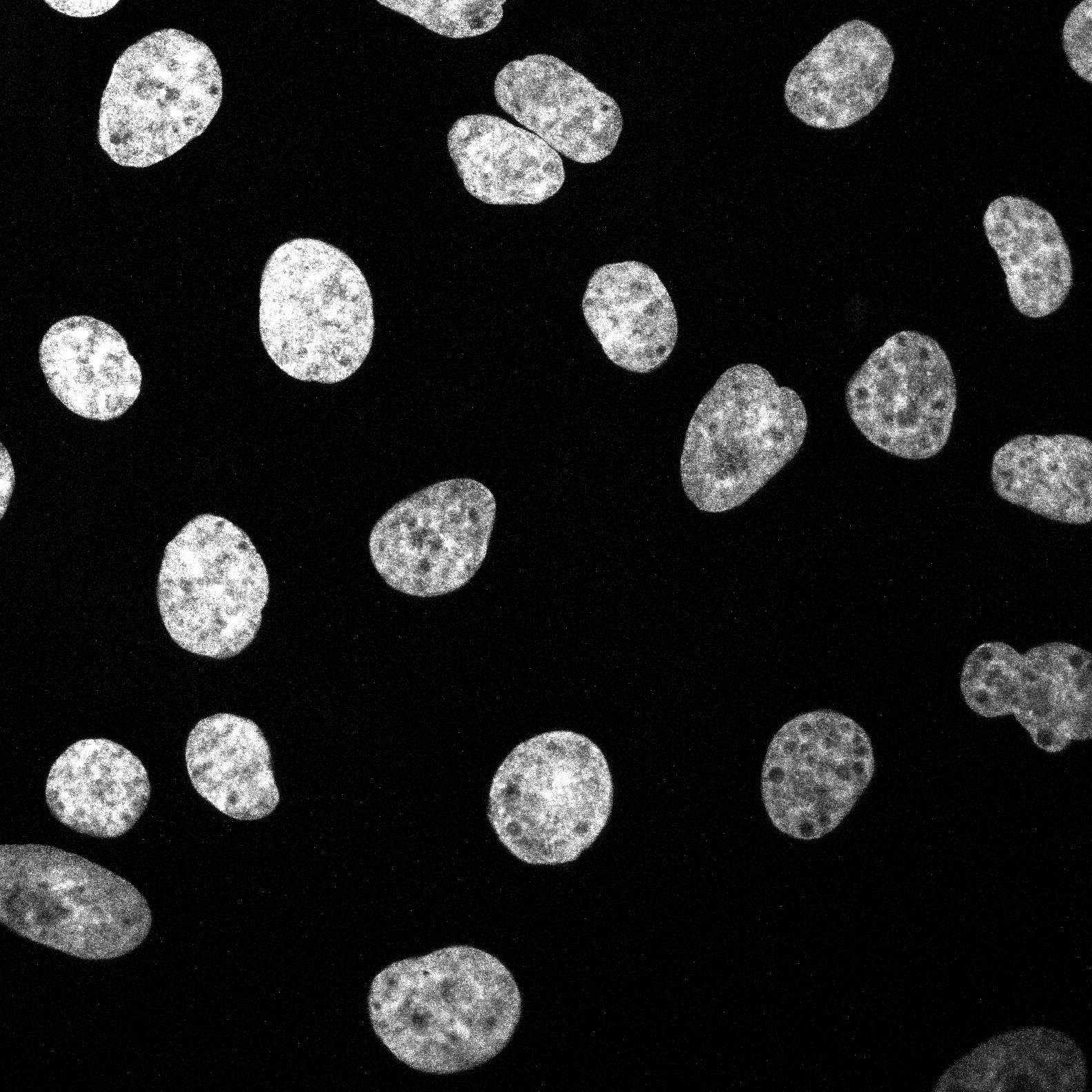

Supplement: Supplementary file 10 — Source Data Fig. 3 [file 44318_2023_21_MOESM10_ESM.zip › Figure 3/Figure 3J/ZMAX_A549 52K S_A_24 hr dox CTL _DAPI Channel (405).tif]

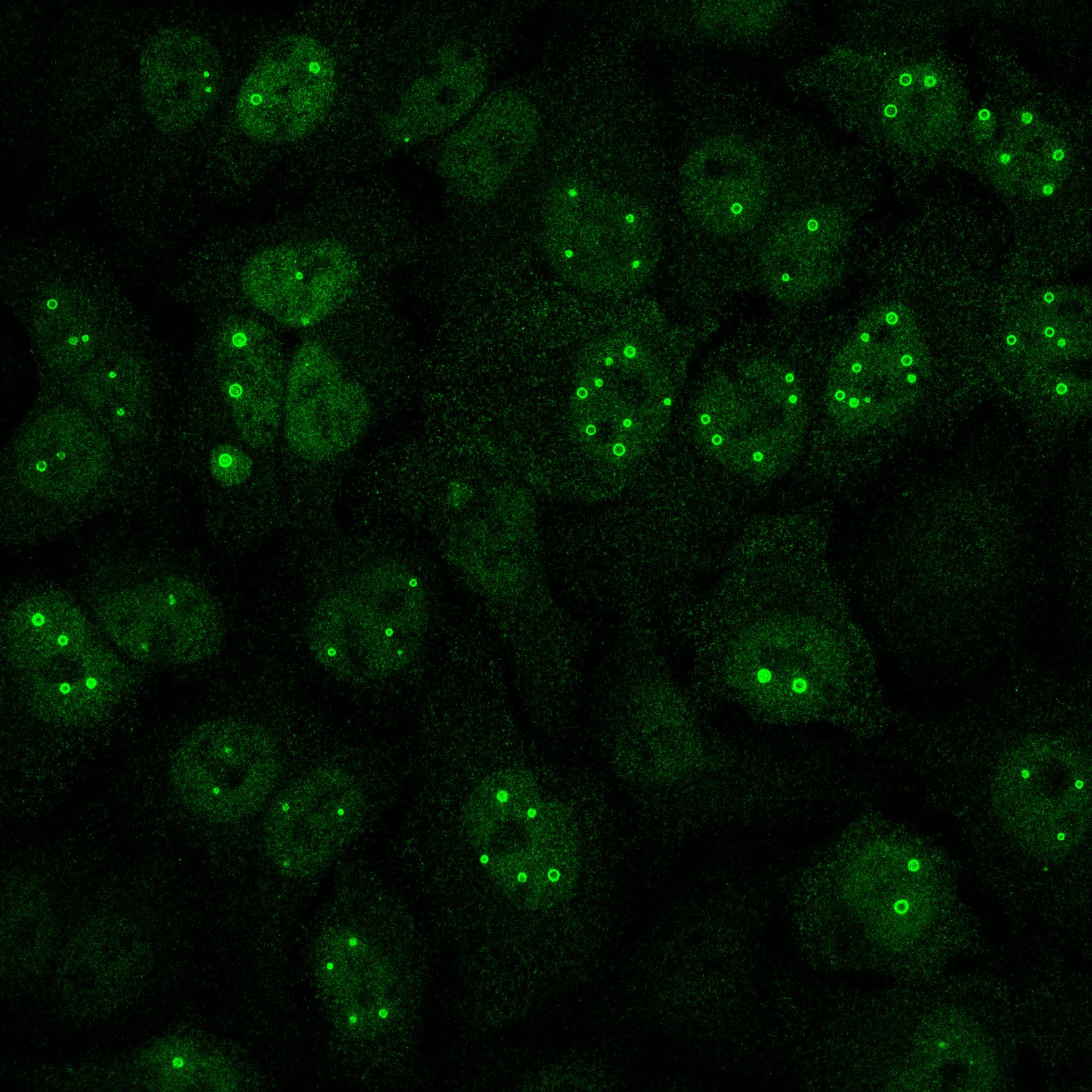

Supplement: Supplementary file 10 — Source Data Fig. 3 [file 44318_2023_21_MOESM10_ESM.zip › Figure 3/Figure 3J/ZMAX_A549 52K S_A_24hr 75mM hex_52K Channel (488).tif]

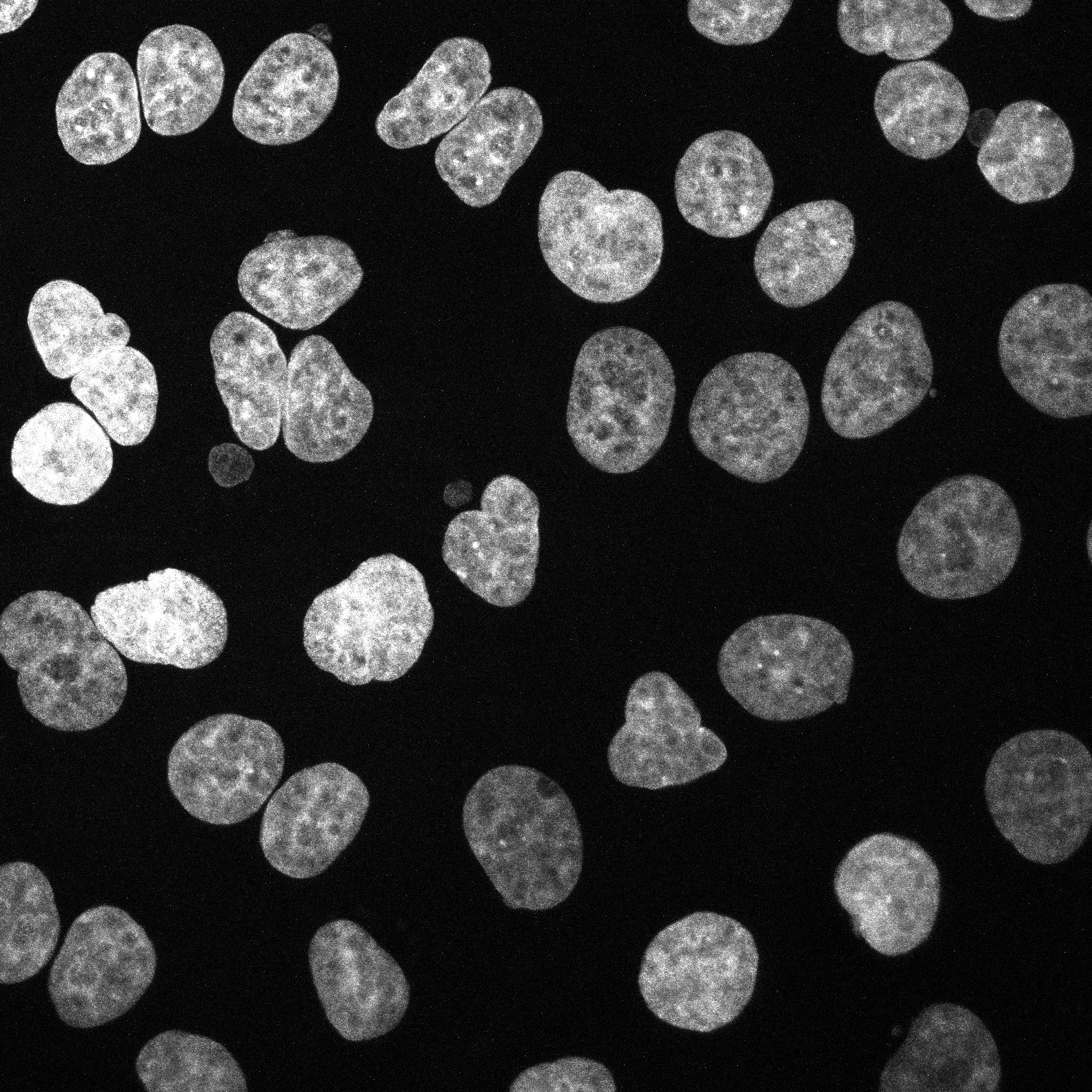

Supplement: Supplementary file 10 — Source Data Fig. 3 [file 44318_2023_21_MOESM10_ESM.zip › Figure 3/Figure 3J/ZMAX_A549 52K S_A_24hr 75mM hex_DAPI Channel (405).tif]

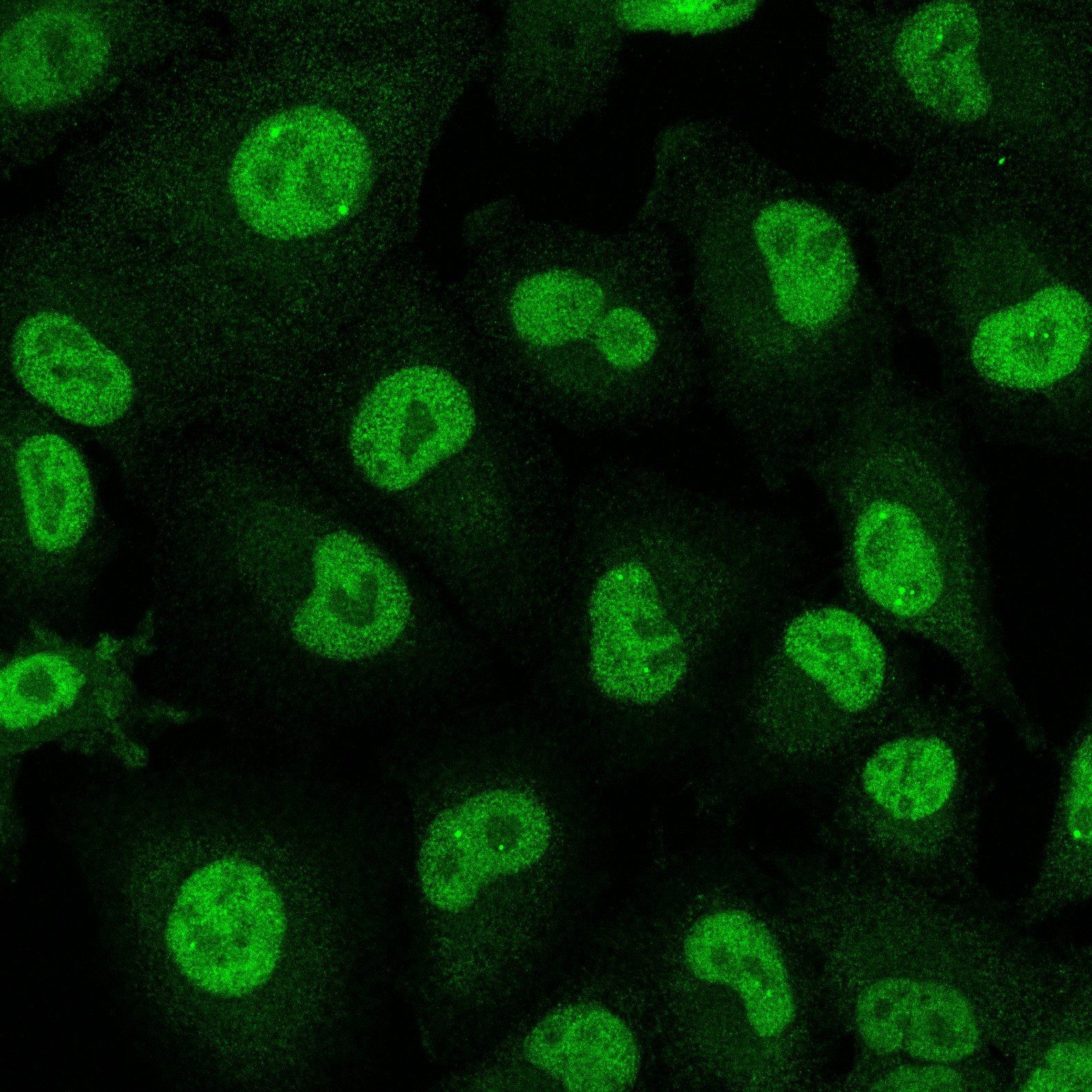

Supplement: Supplementary file 10 — Source Data Fig. 3 [file 44318_2023_21_MOESM10_ESM.zip › Figure 3/Figure 3J/ZMAX_A549 52K S_A_8 hr dox CTL_52K Channel (488).tif]

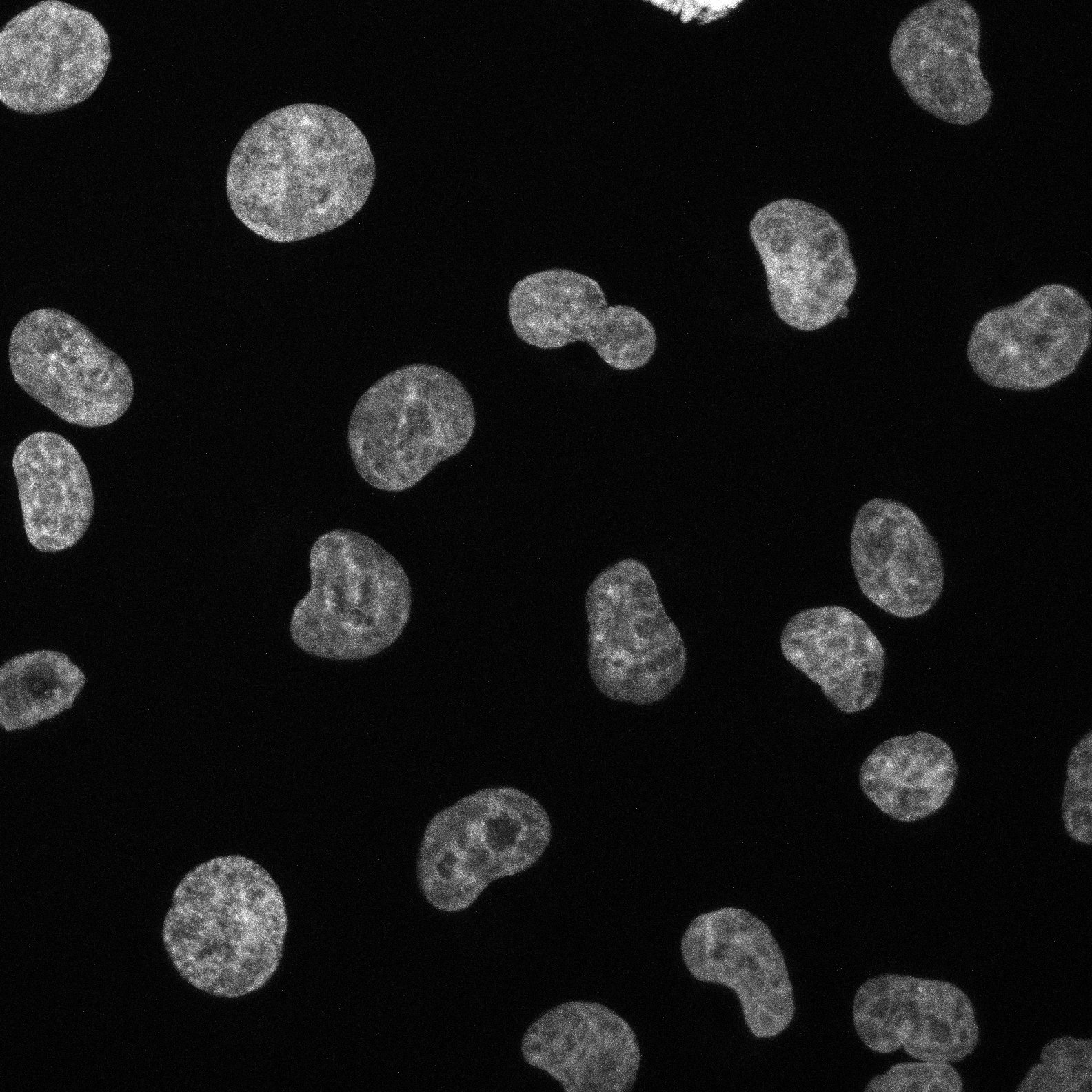

Supplement: Supplementary file 10 — Source Data Fig. 3 [file 44318_2023_21_MOESM10_ESM.zip › Figure 3/Figure 3J/ZMAX_A549 52K S_A_8 hr dox CTL_DAPI Channel (405).tif]

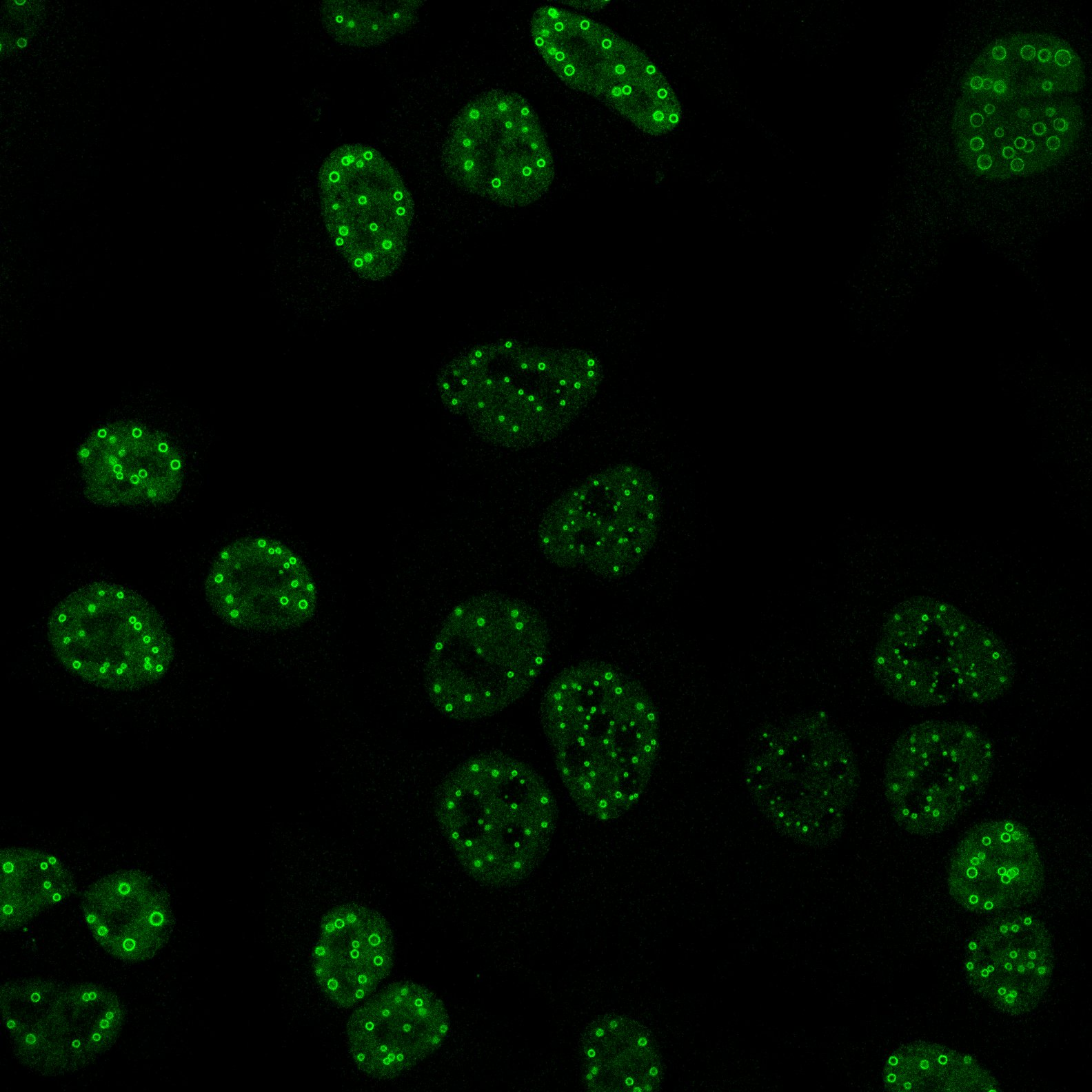

Supplement: Supplementary file 10 — Source Data Fig. 3 [file 44318_2023_21_MOESM10_ESM.zip › Figure 3/Figure 3J/ZMAX_A549 52K S_D_24 hr dox CTL_52K Channel (488).tif]

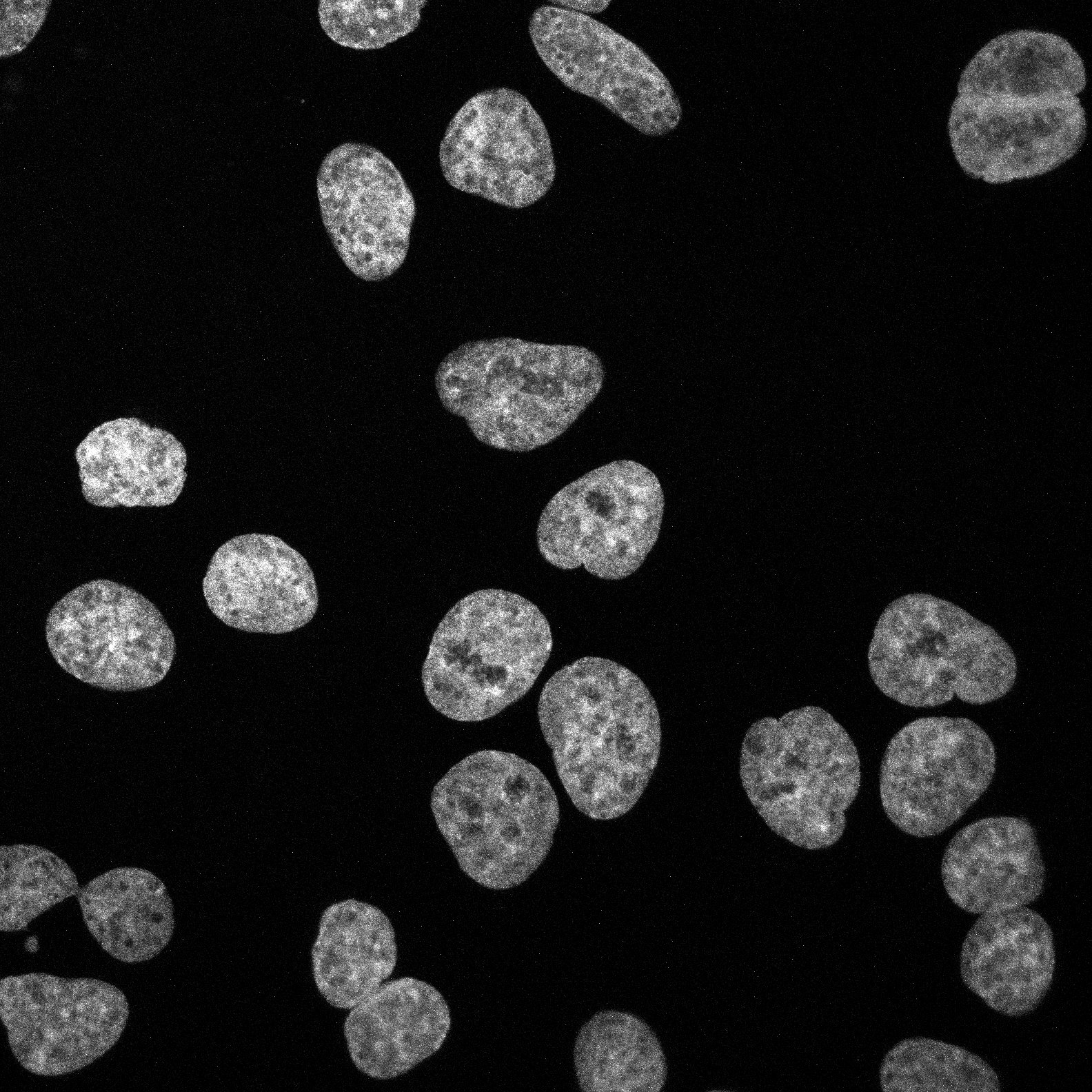

Supplement: Supplementary file 10 — Source Data Fig. 3 [file 44318_2023_21_MOESM10_ESM.zip › Figure 3/Figure 3J/ZMAX_A549 52K S_D_24 hr dox CTL_DAPI Channel (405).tif]

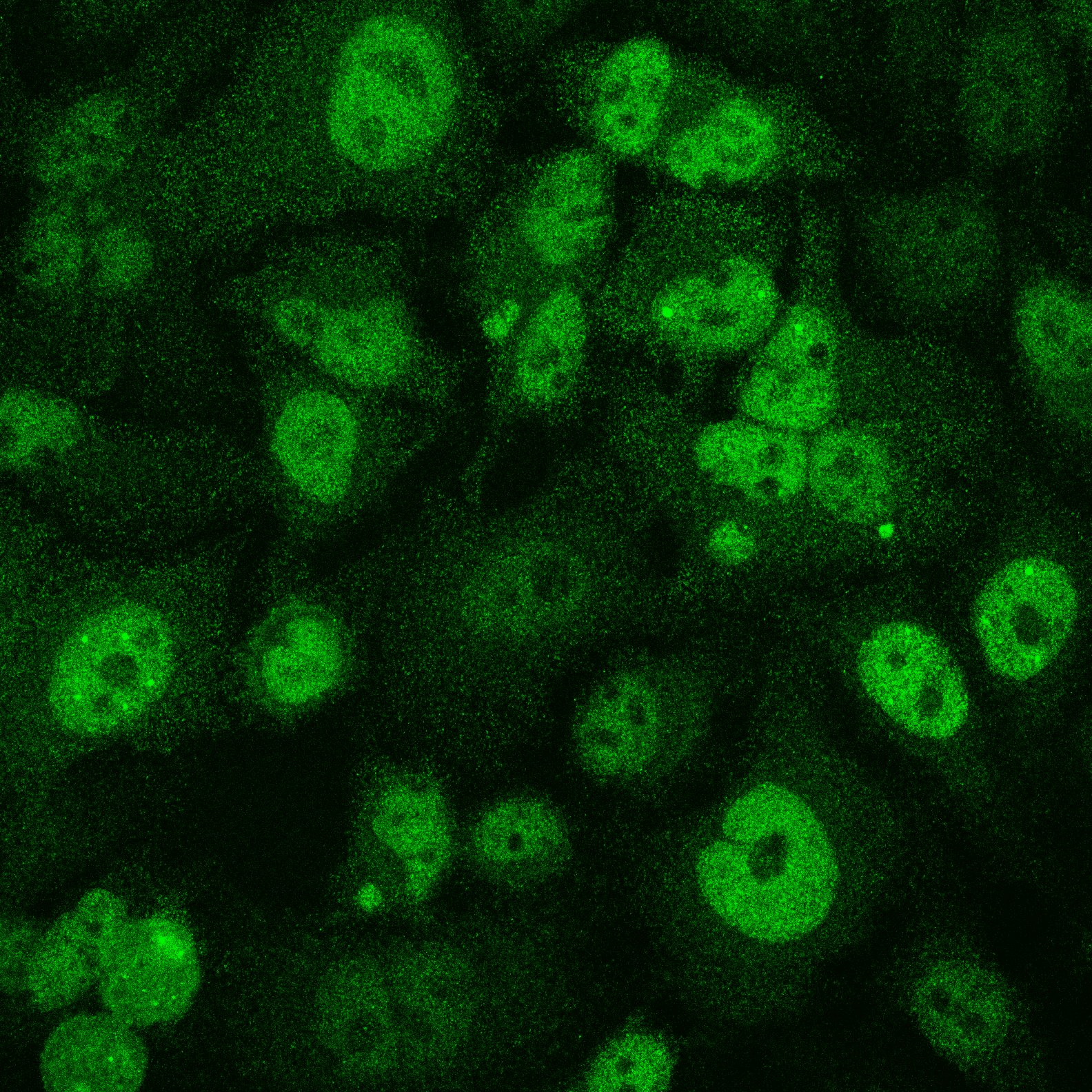

Supplement: Supplementary file 10 — Source Data Fig. 3 [file 44318_2023_21_MOESM10_ESM.zip › Figure 3/Figure 3J/ZMAX_A549 52K S_D_24hr 75mM hex_52K Channel (488).tif]

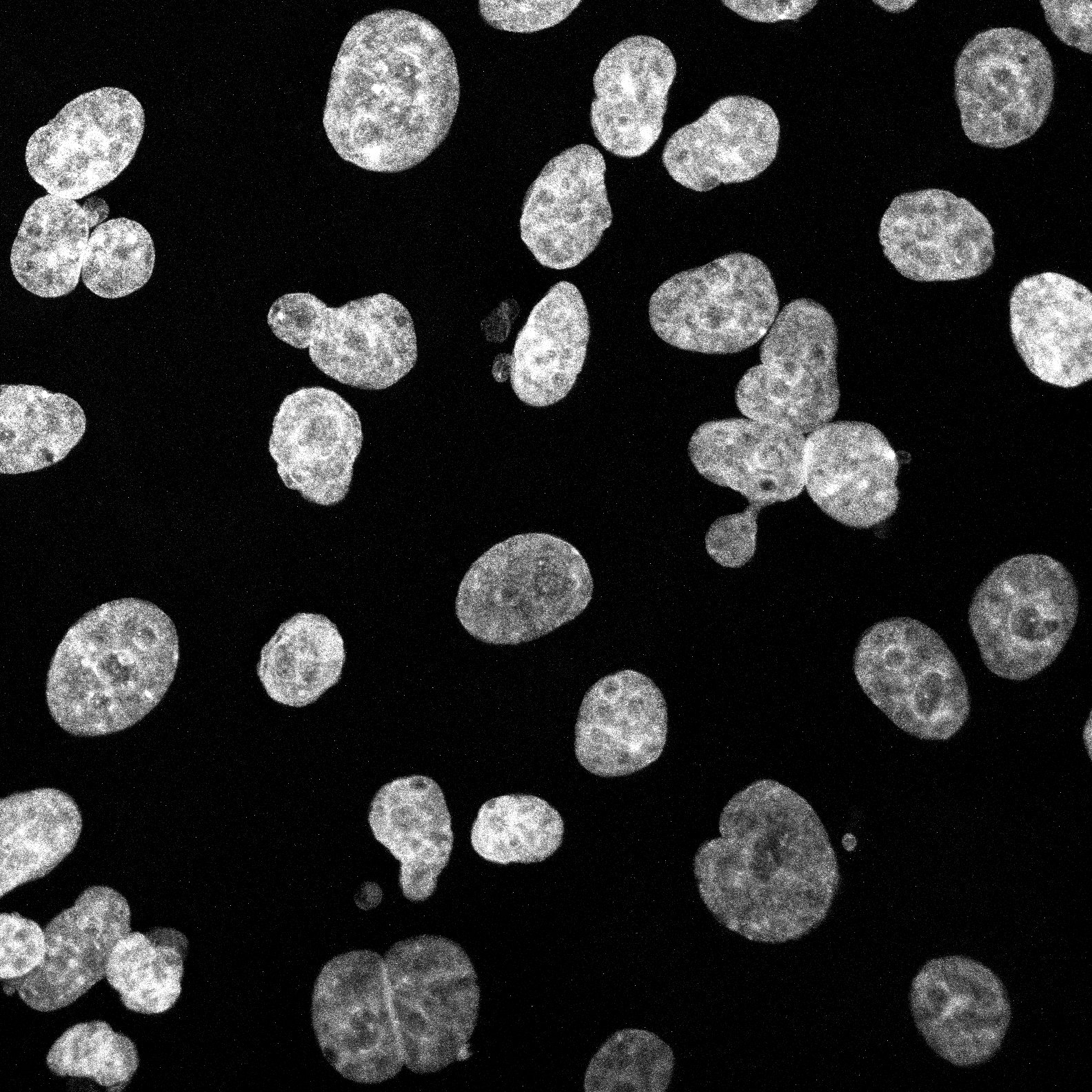

Supplement: Supplementary file 10 — Source Data Fig. 3 [file 44318_2023_21_MOESM10_ESM.zip › Figure 3/Figure 3J/ZMAX_A549 52K S_D_24hr 75mM hex_DAPI Channel (405).tif]

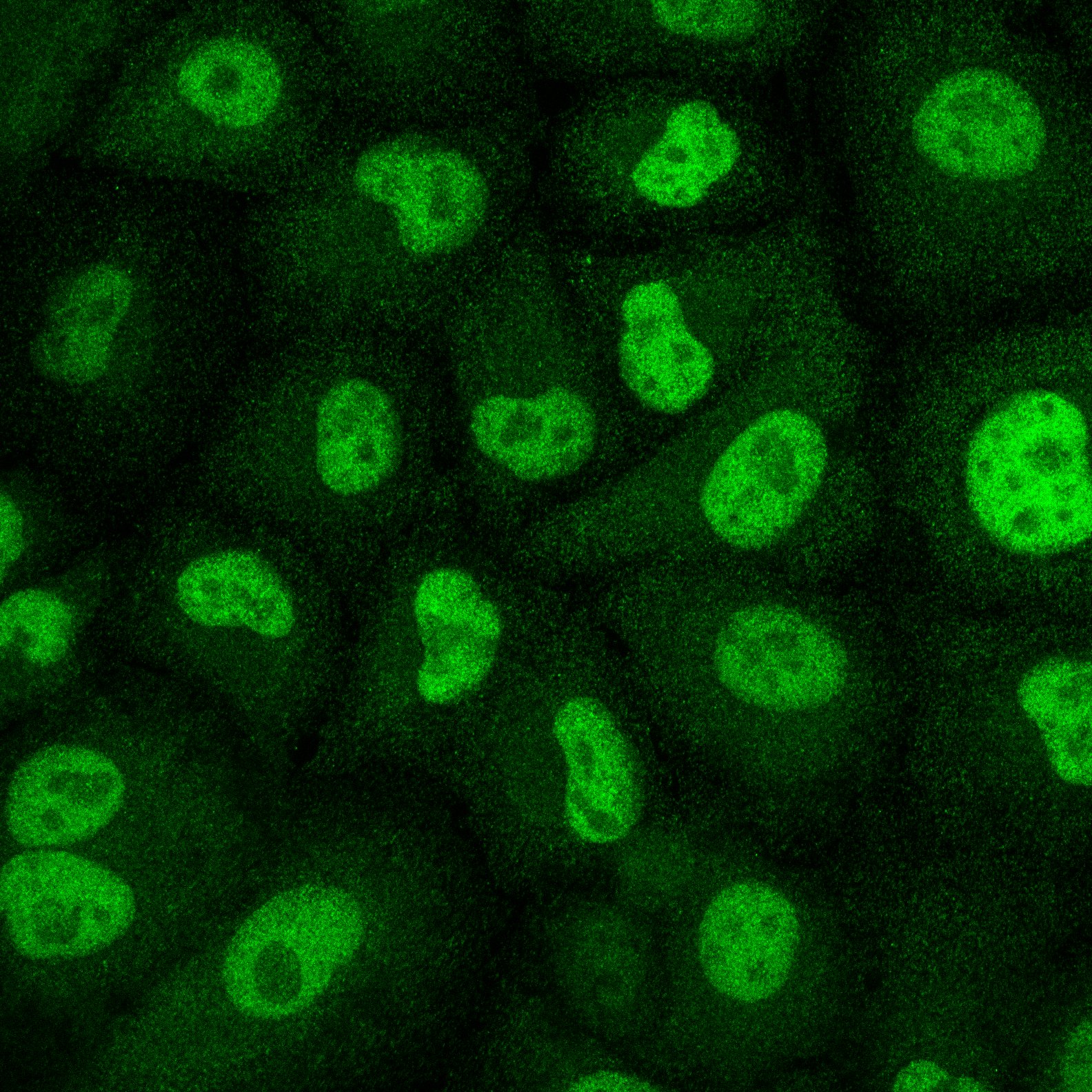

Supplement: Supplementary file 10 — Source Data Fig. 3 [file 44318_2023_21_MOESM10_ESM.zip › Figure 3/Figure 3J/ZMAX_A549 52K S_D_8 hr dox CTL_52K Channel (488).tif]

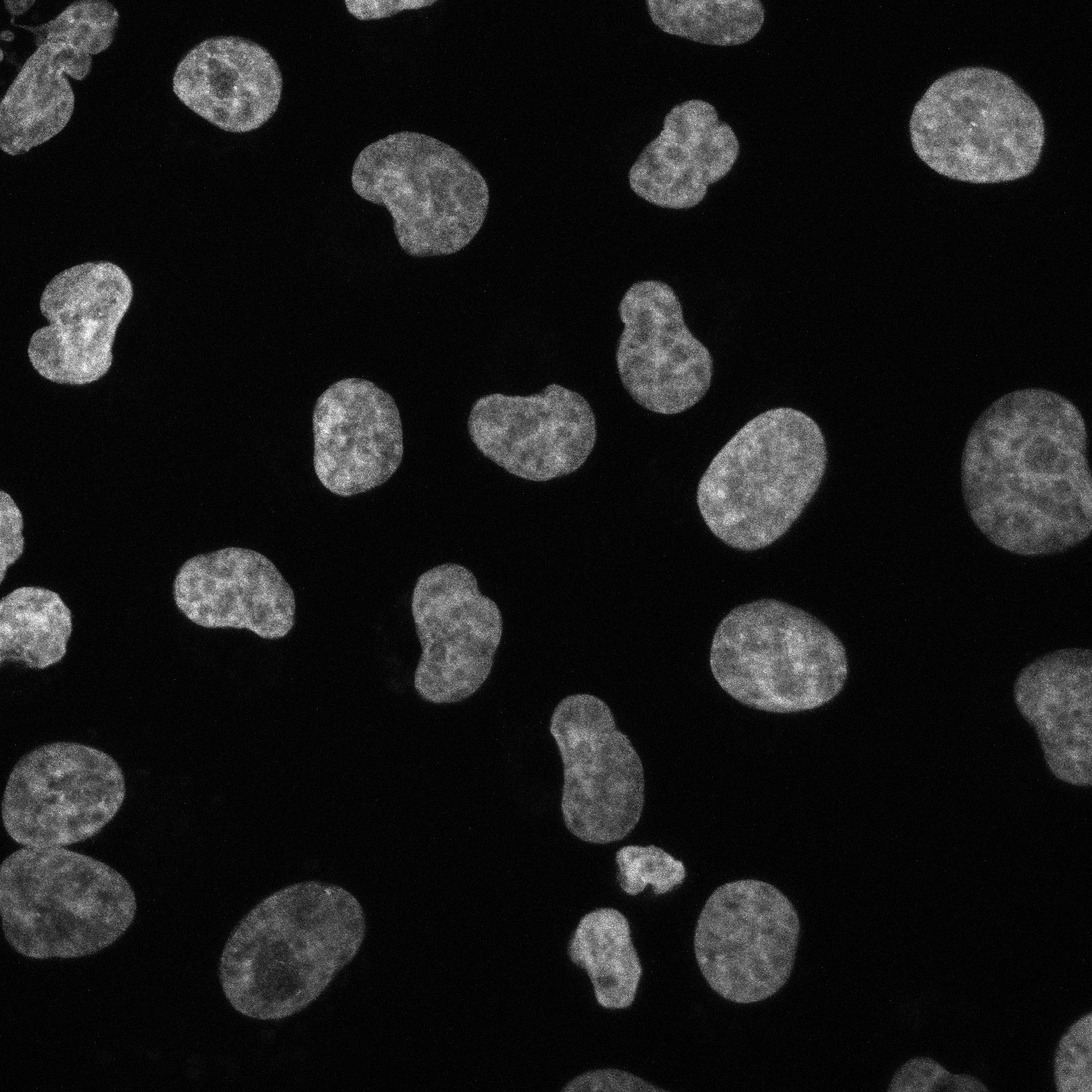

Supplement: Supplementary file 10 — Source Data Fig. 3 [file 44318_2023_21_MOESM10_ESM.zip › Figure 3/Figure 3J/ZMAX_A549 52K S_D_8 hr dox CTL_DAPI Channel (405).tif]

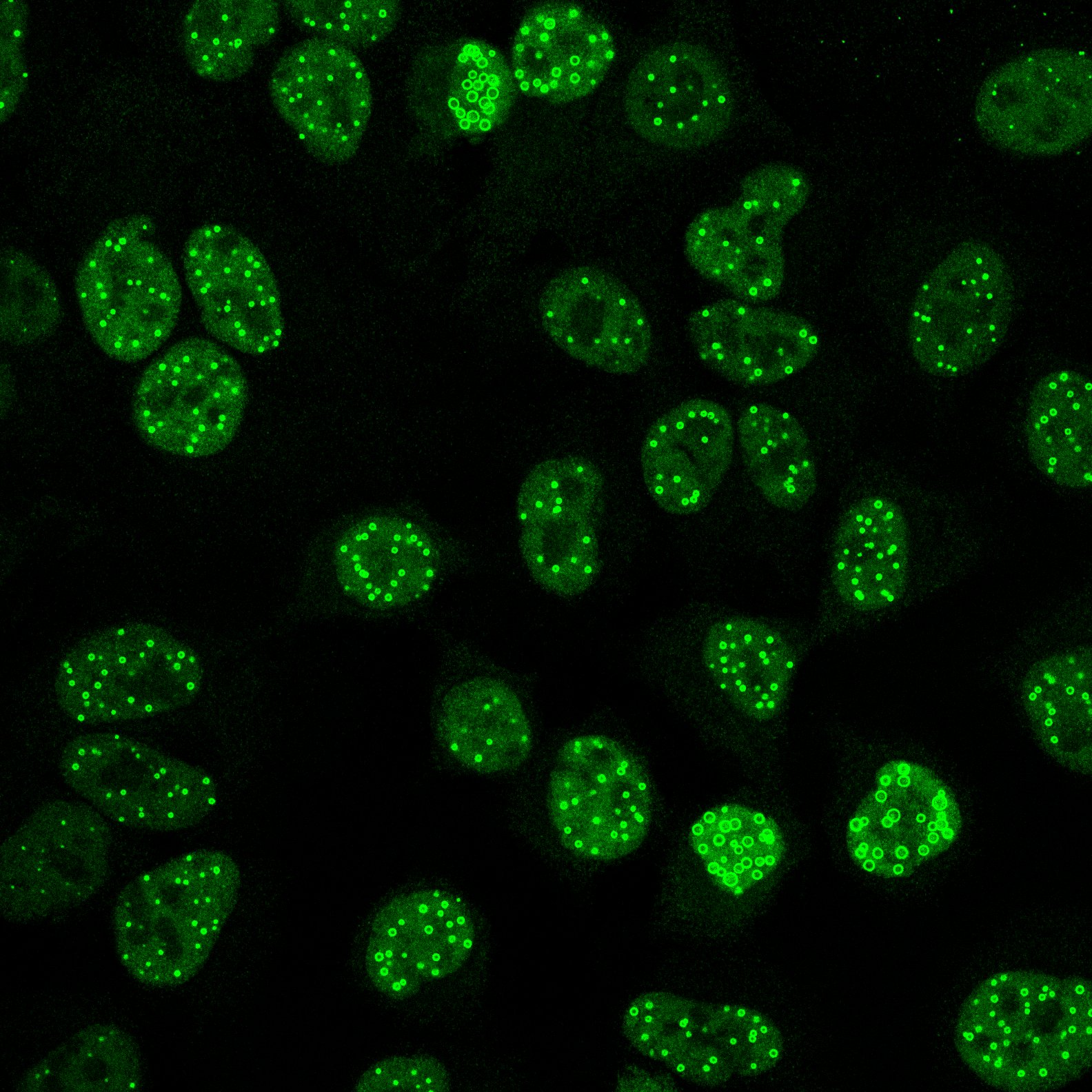

Supplement: Supplementary file 10 — Source Data Fig. 3 [file 44318_2023_21_MOESM10_ESM.zip › Figure 3/Figure 3J/ZMAX_A549 52K WT_24 hr dox CTL _52K Channel (488).tif]

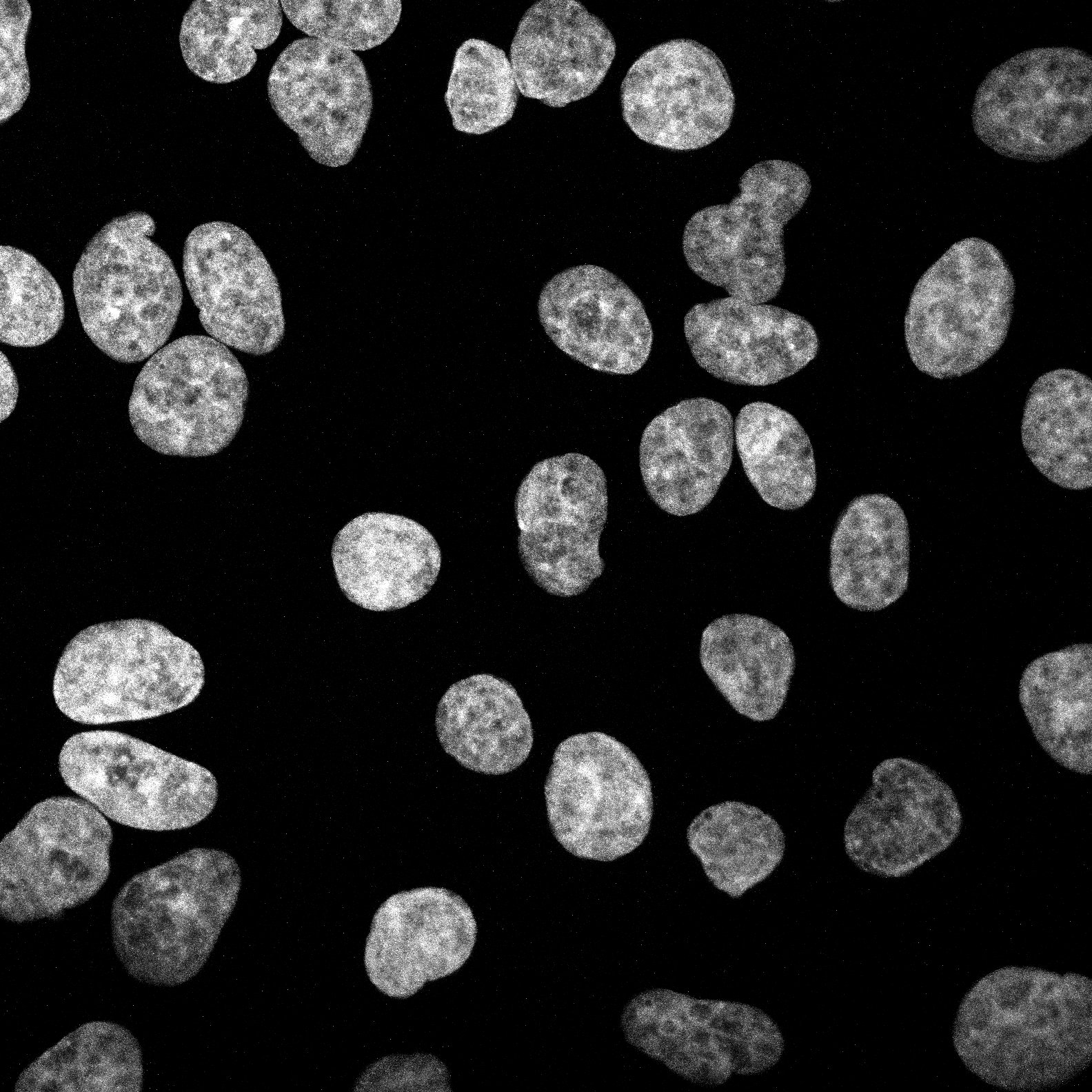

Supplement: Supplementary file 10 — Source Data Fig. 3 [file 44318_2023_21_MOESM10_ESM.zip › Figure 3/Figure 3J/ZMAX_A549 52K WT_24 hr dox CTL _DAPI Channel (405).tif]

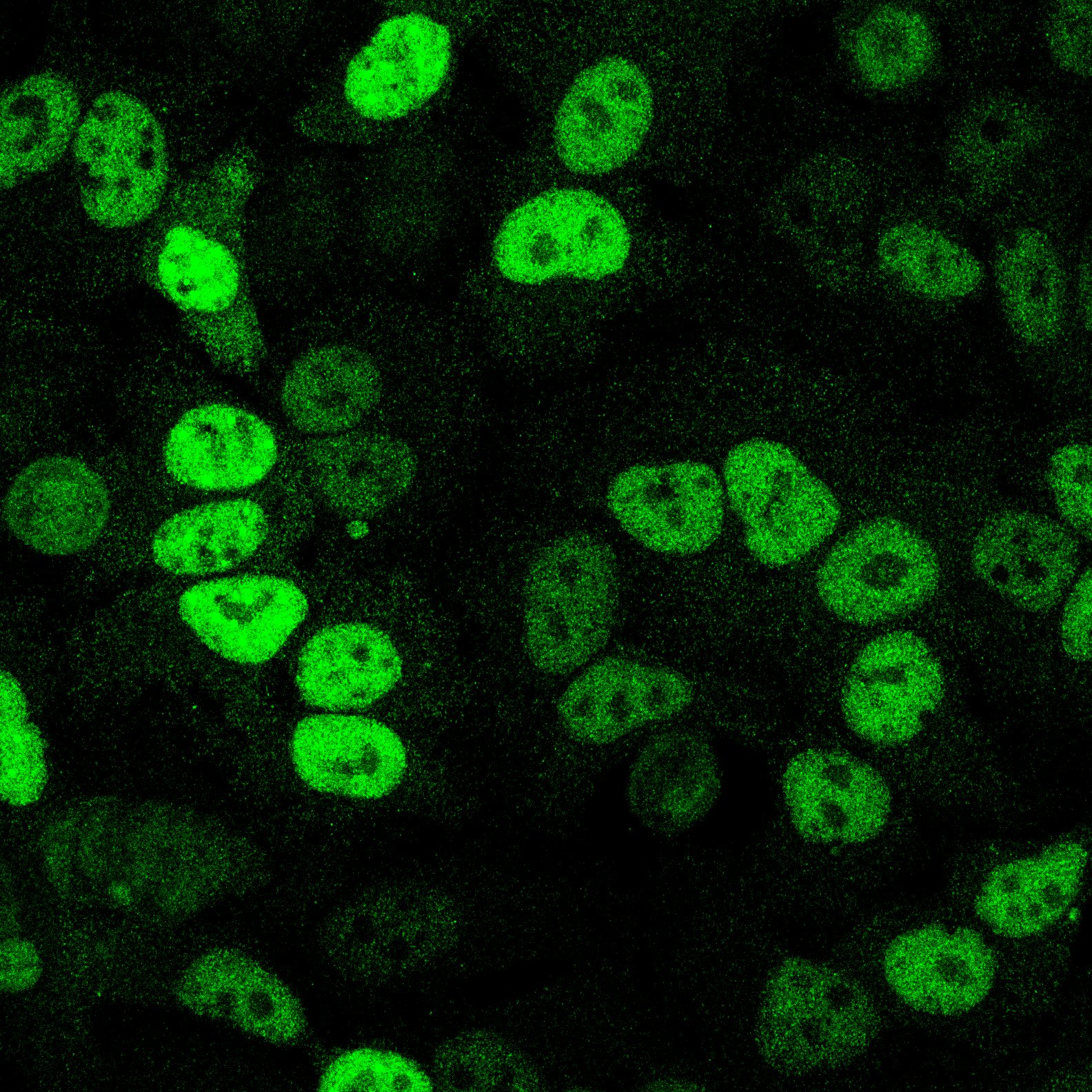

Supplement: Supplementary file 10 — Source Data Fig. 3 [file 44318_2023_21_MOESM10_ESM.zip › Figure 3/Figure 3J/ZMAX_A549 52K WT_24hr 75mM hex_52K Channel (488).tif]

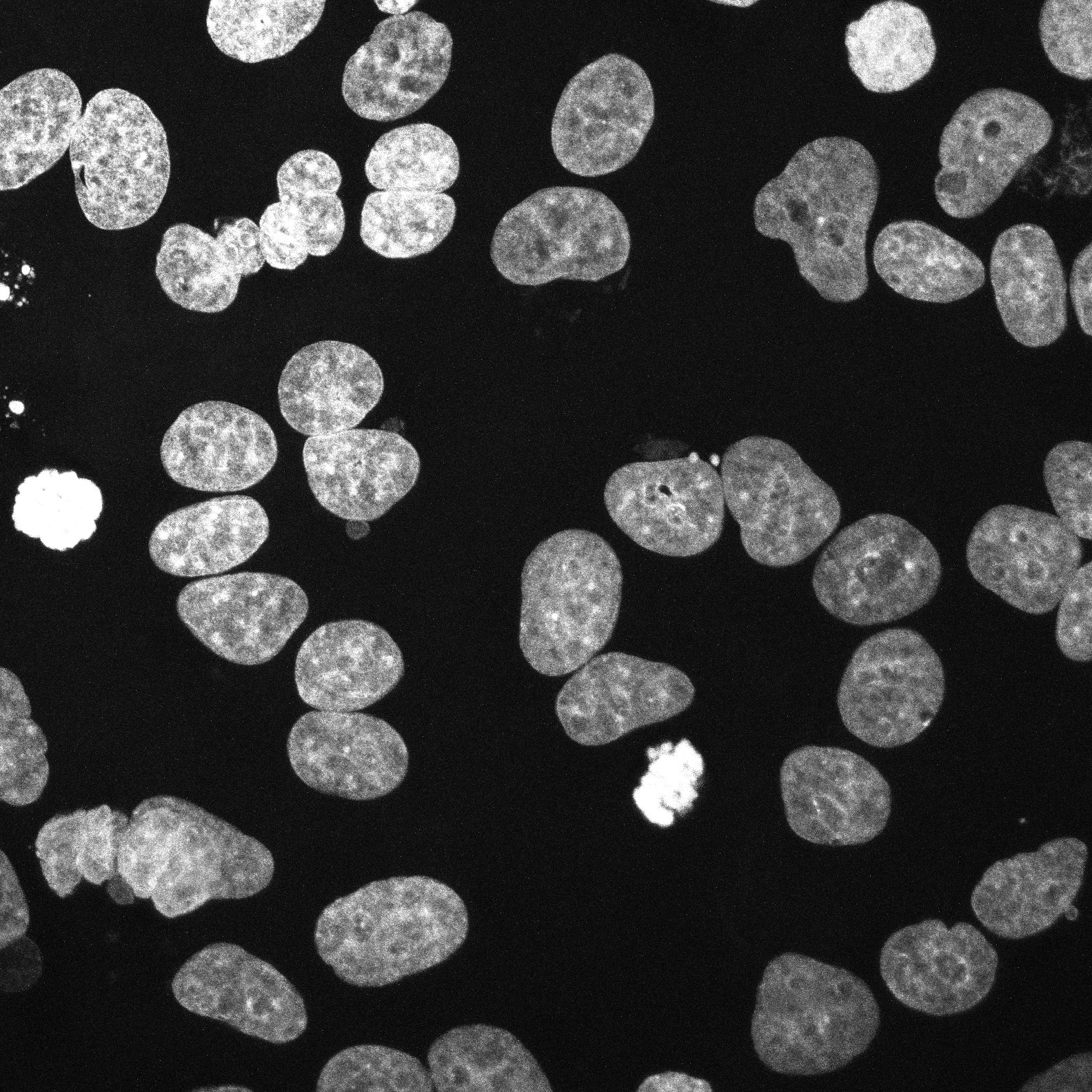

Supplement: Supplementary file 10 — Source Data Fig. 3 [file 44318_2023_21_MOESM10_ESM.zip › Figure 3/Figure 3J/ZMAX_A549 52K WT_24hr 75mM hex_DAPI Channel (405).tif]

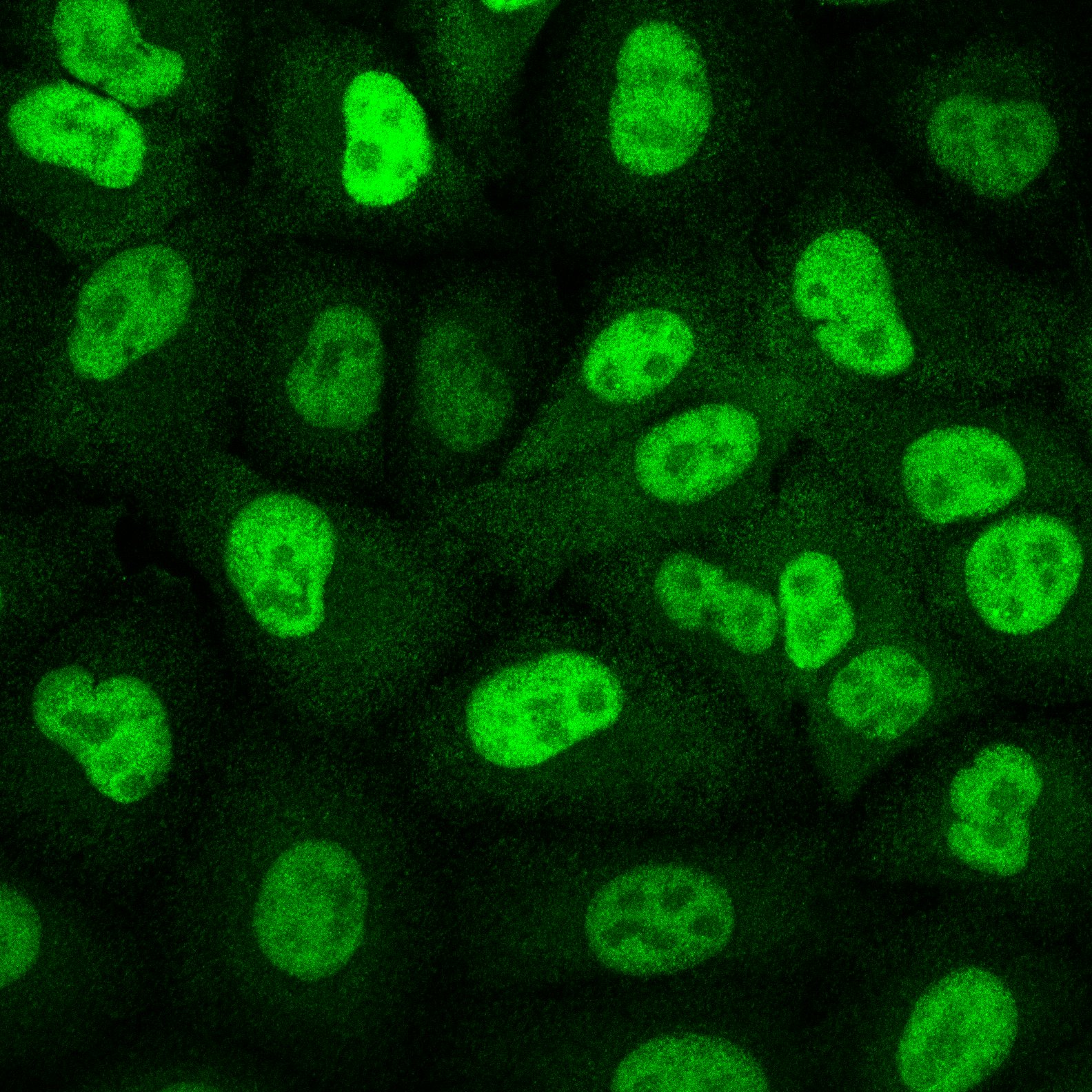

Supplement: Supplementary file 10 — Source Data Fig. 3 [file 44318_2023_21_MOESM10_ESM.zip › Figure 3/Figure 3J/ZMAX_A549 52K WT_8 hr dox CTL_ 52K Channel (488).tif]

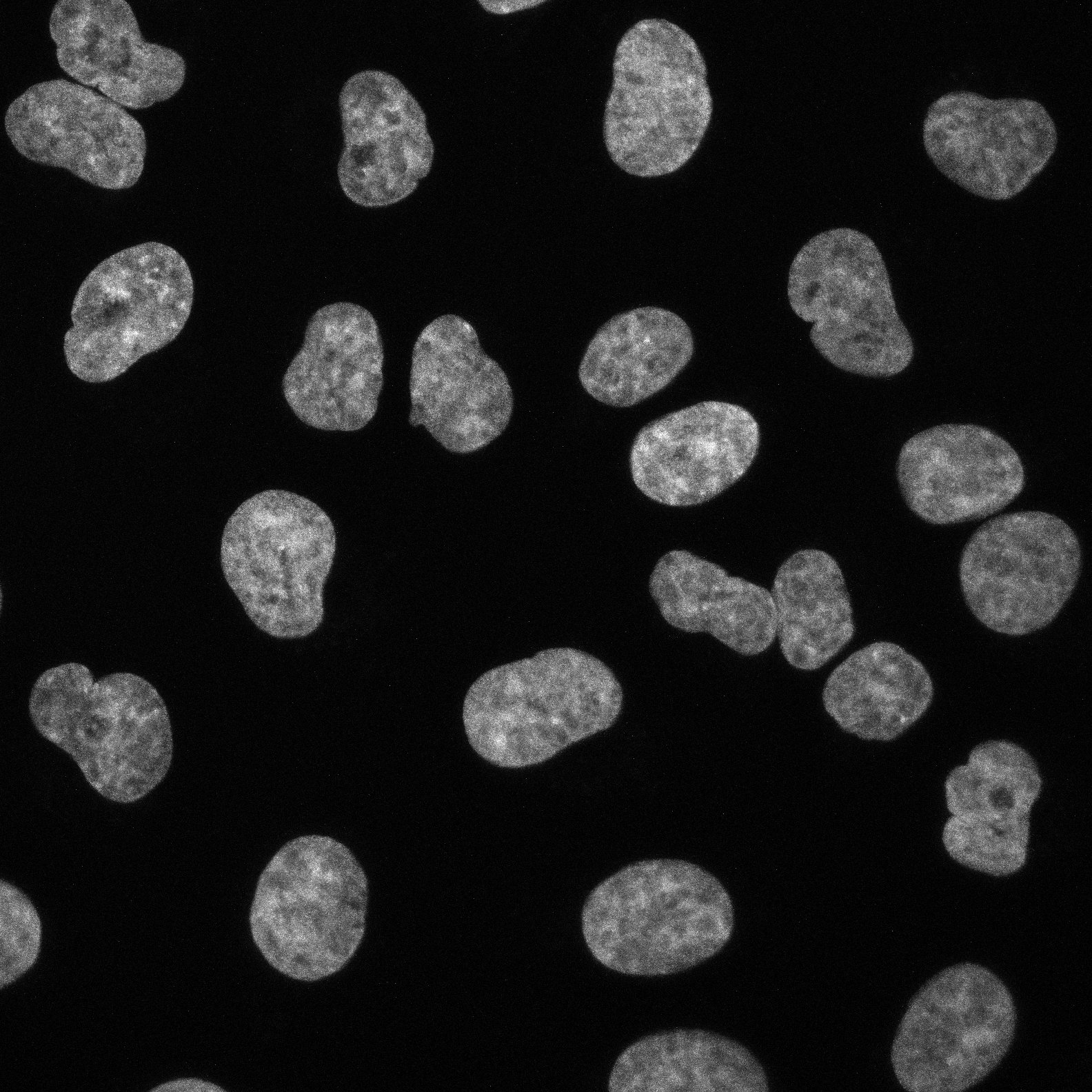

Supplement: Supplementary file 10 — Source Data Fig. 3 [file 44318_2023_21_MOESM10_ESM.zip › Figure 3/Figure 3J/ZMAX_A549 52K WT_8 hr dox CTL_ DAPI Channel (405).tif]

Figure 4: Panel A

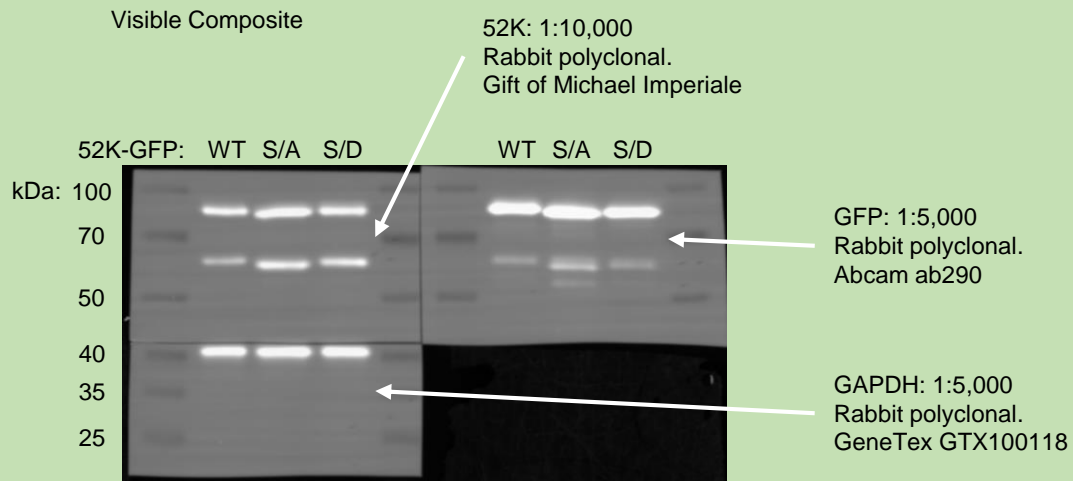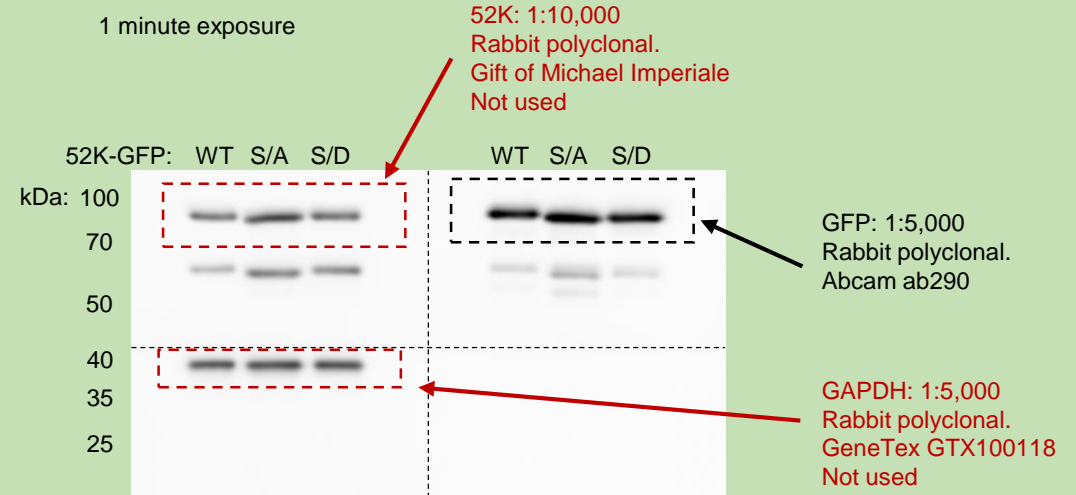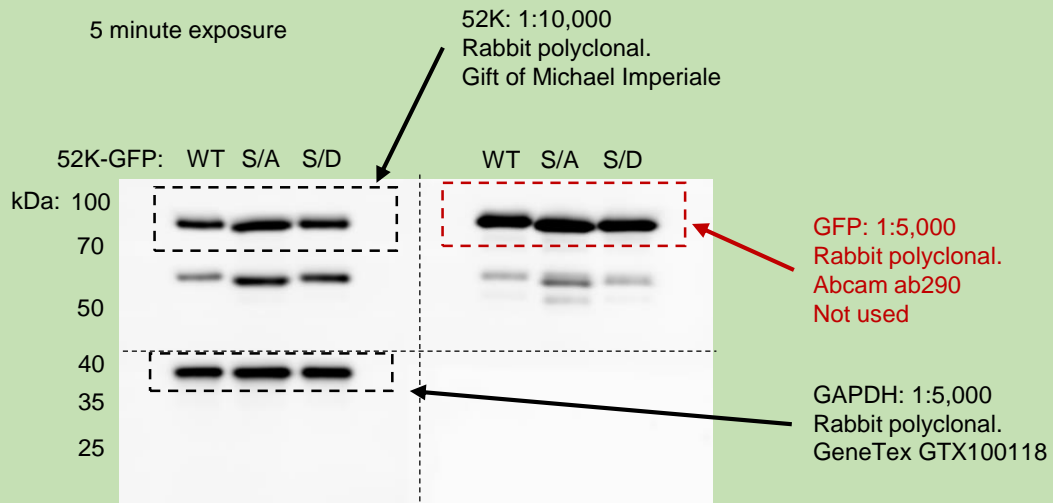

Supplement: Supplementary file 11 — Source Data Fig. 4 [file 44318_2023_21_MOESM11_ESM.zip › Figure 4/Figure 4A/Panel A_52K-GFP expression in HEK293 for FRAP experiments.pdf]

# Figure 5: Panel A

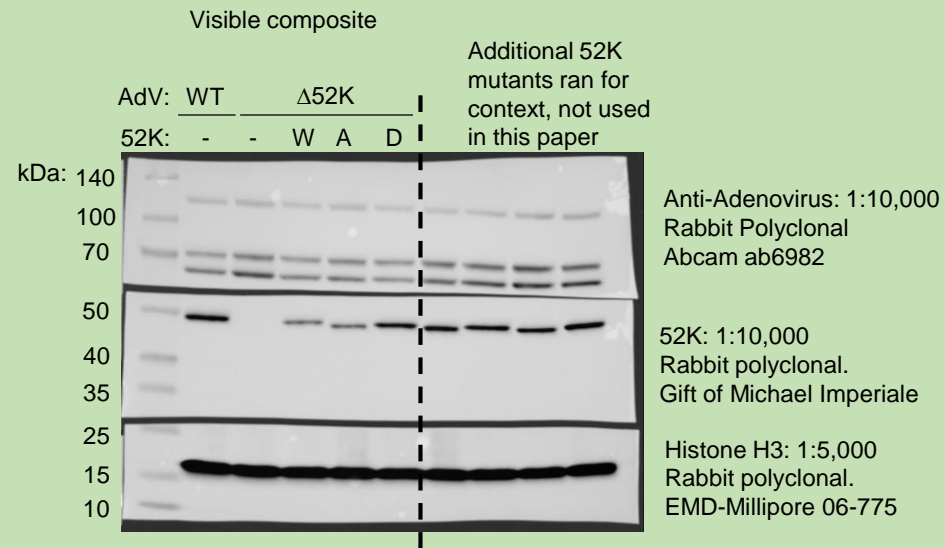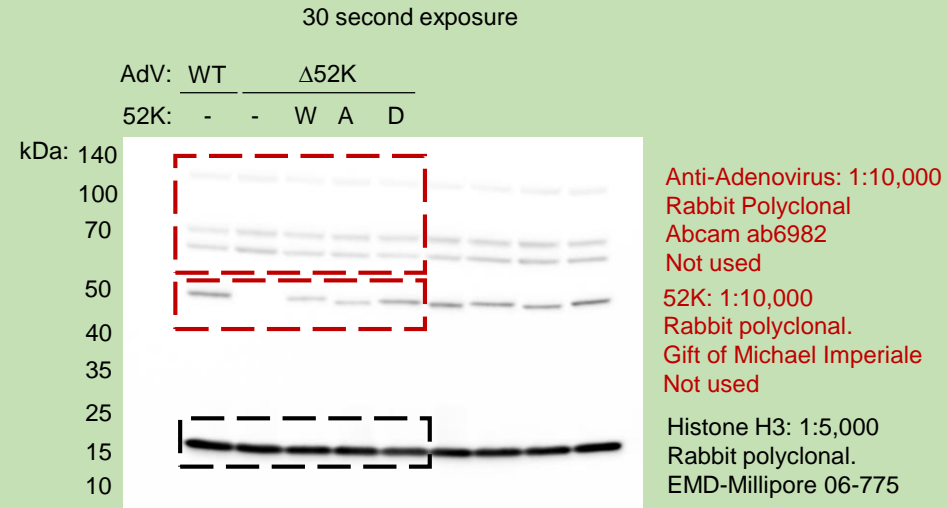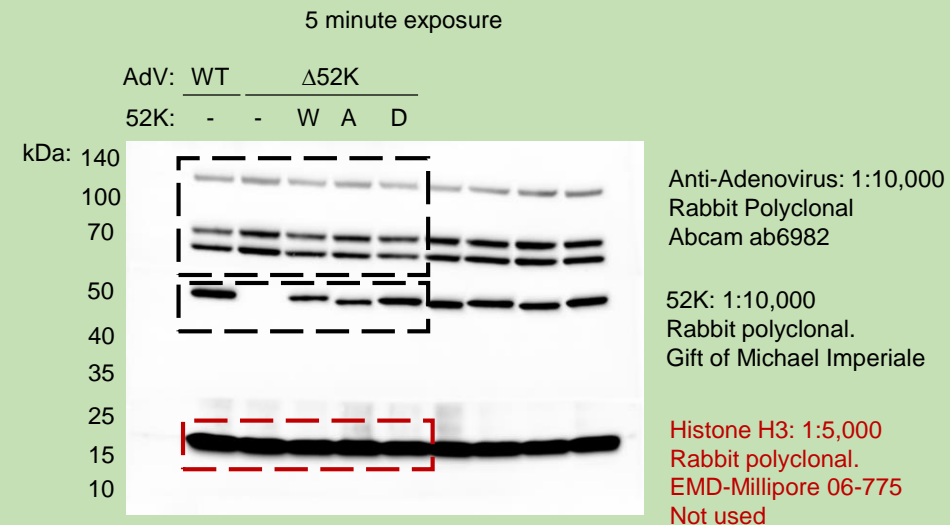

Supplement: Supplementary file 12 — Source Data Fig. 5 [file 44318_2023_21_MOESM12_ESM.zip › Figure 5/Figure 5B/Panel B_Rescue of delta52K AdV in 52K transfected 293 cells_Immunoblot.pdf]

Figure 5: Panel C

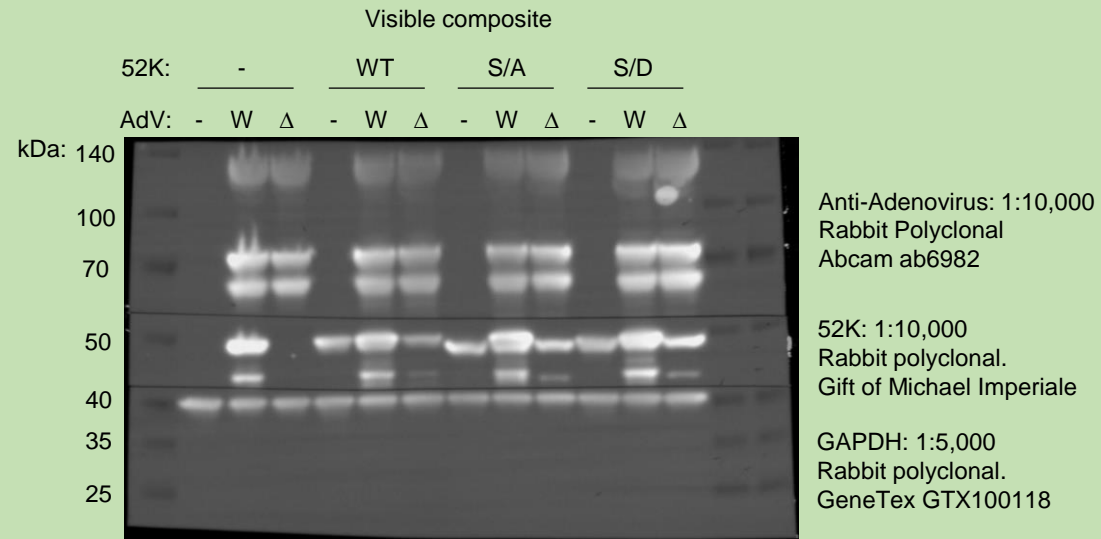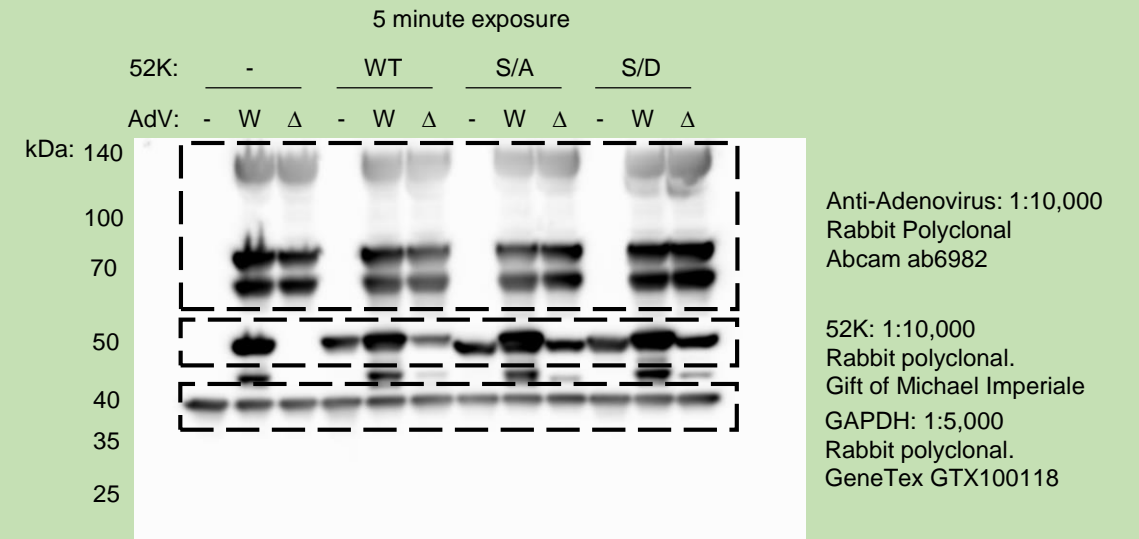

Supplement: Supplementary file 12 — Source Data Fig. 5 [file 44318_2023_21_MOESM12_ESM.zip › Figure 5/Figure 5D/Panel D_Delta 52K AdV Rescue in transgenic A549 52K cell lines_Immunoblot.pdf]

Figure 5: Panel H

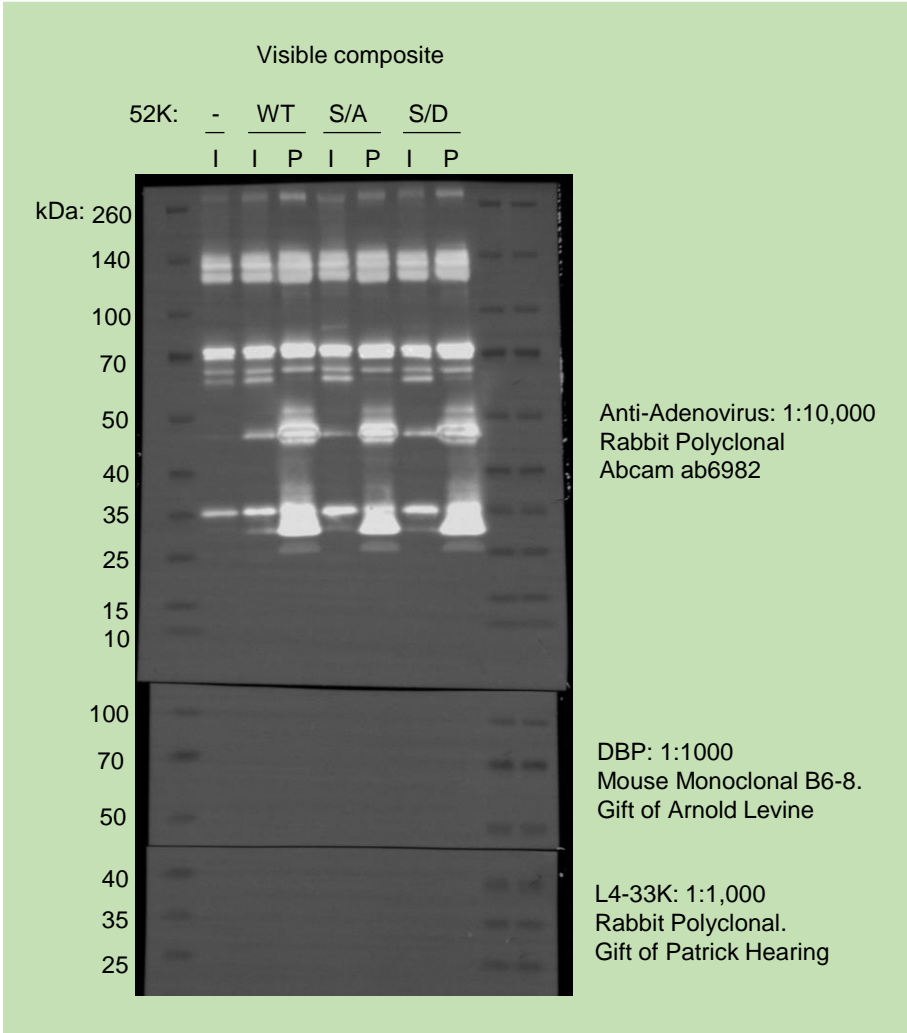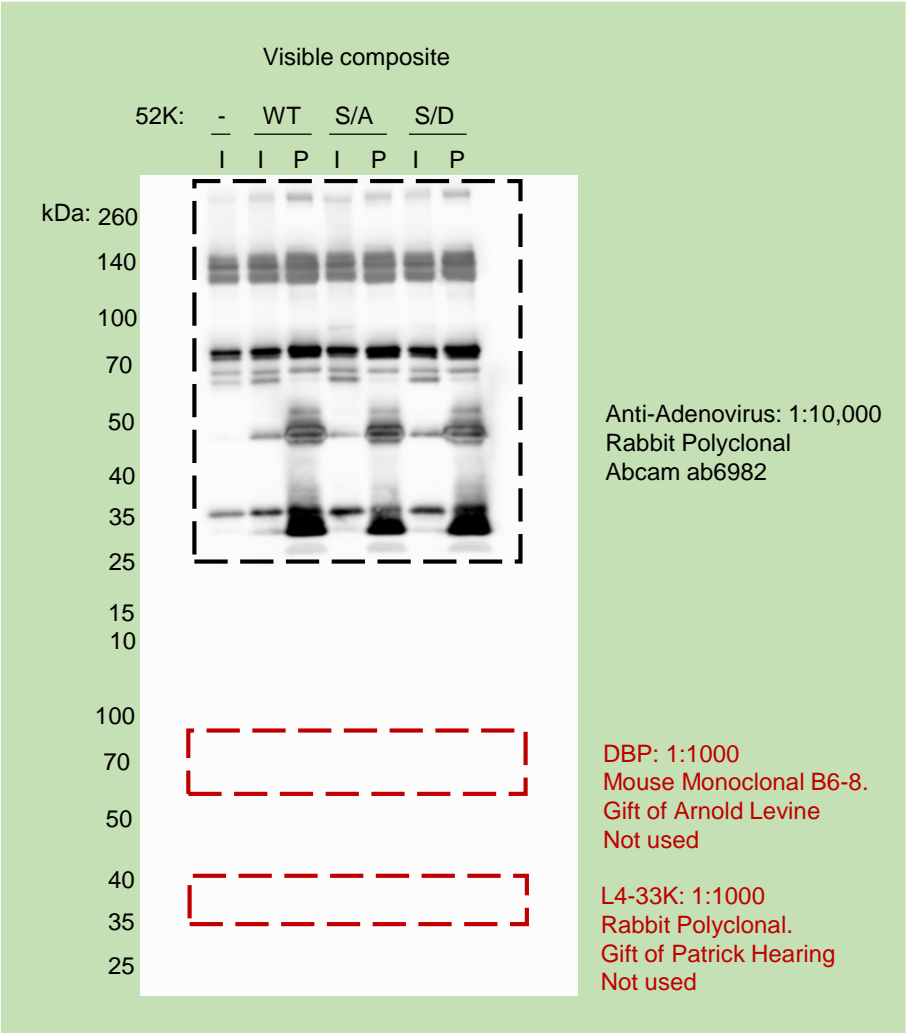

Figure 5: Panel H continued

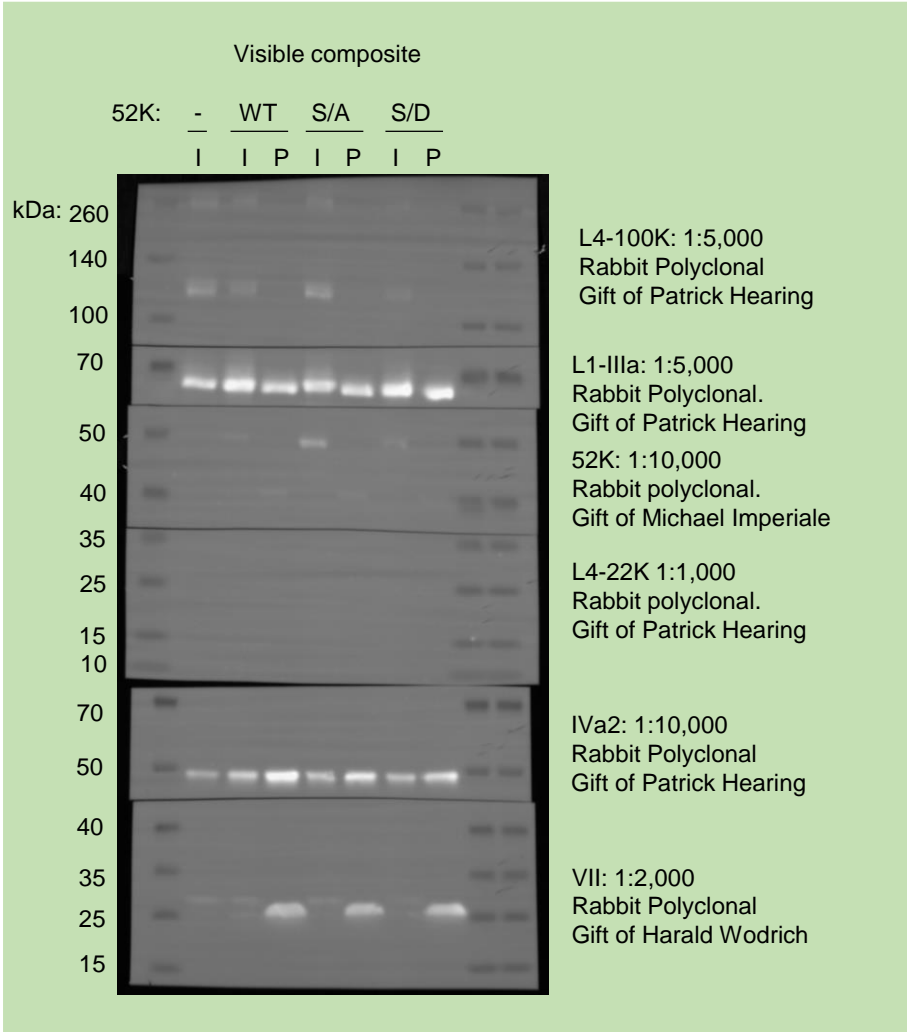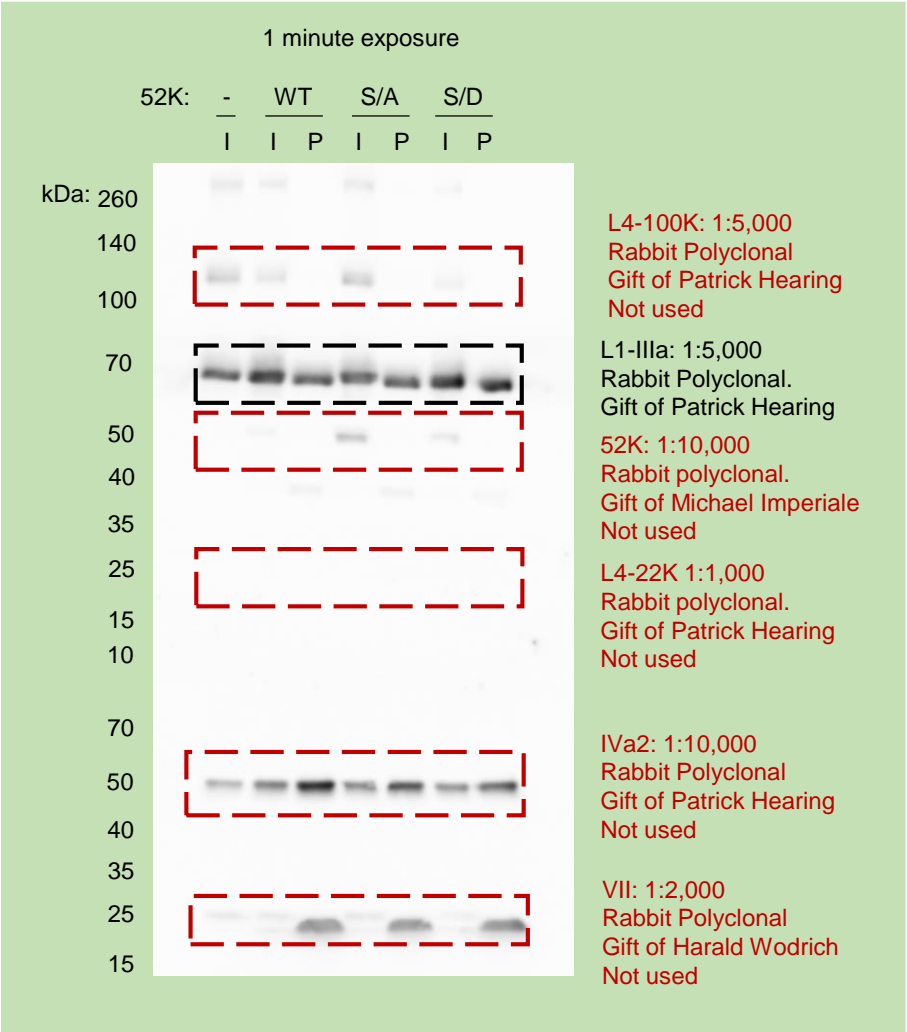

Figure 5: Panel H continued

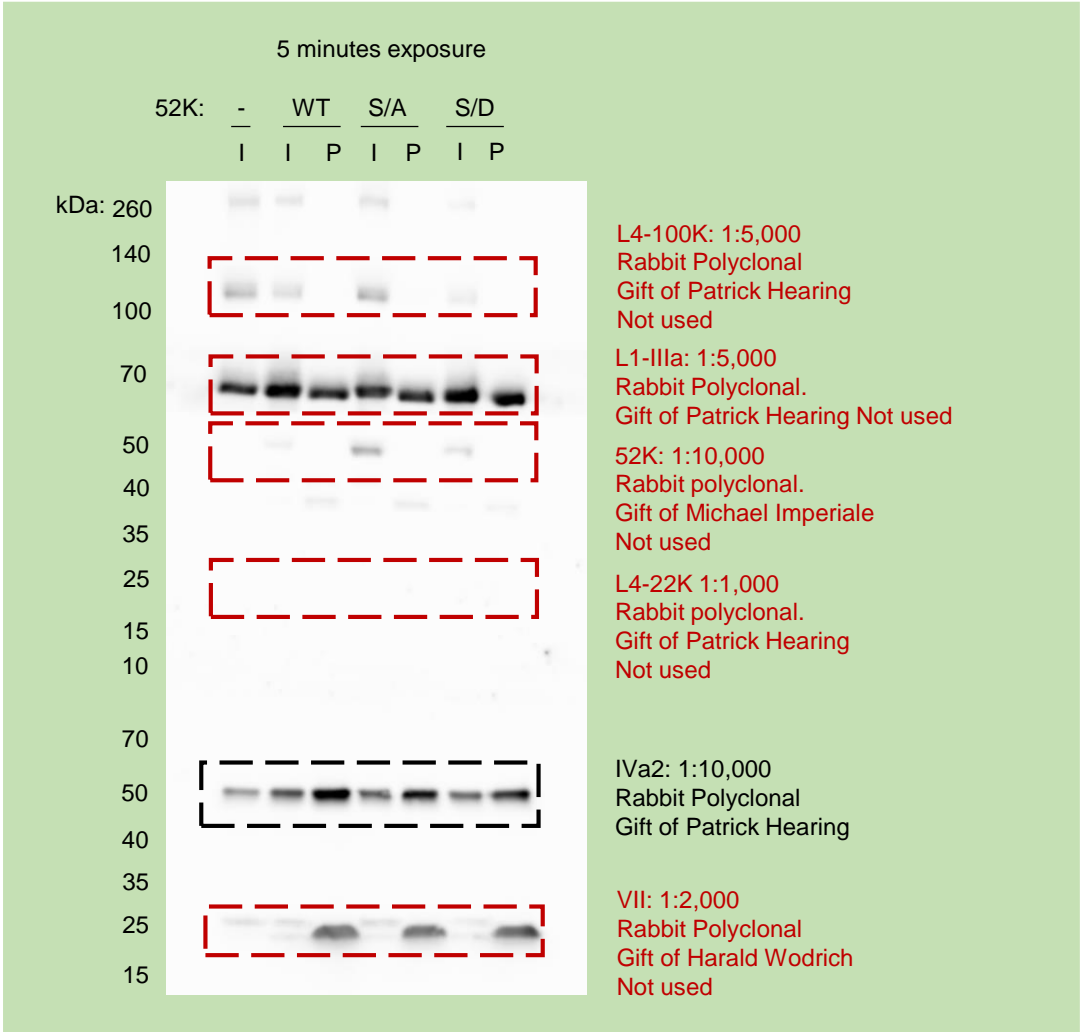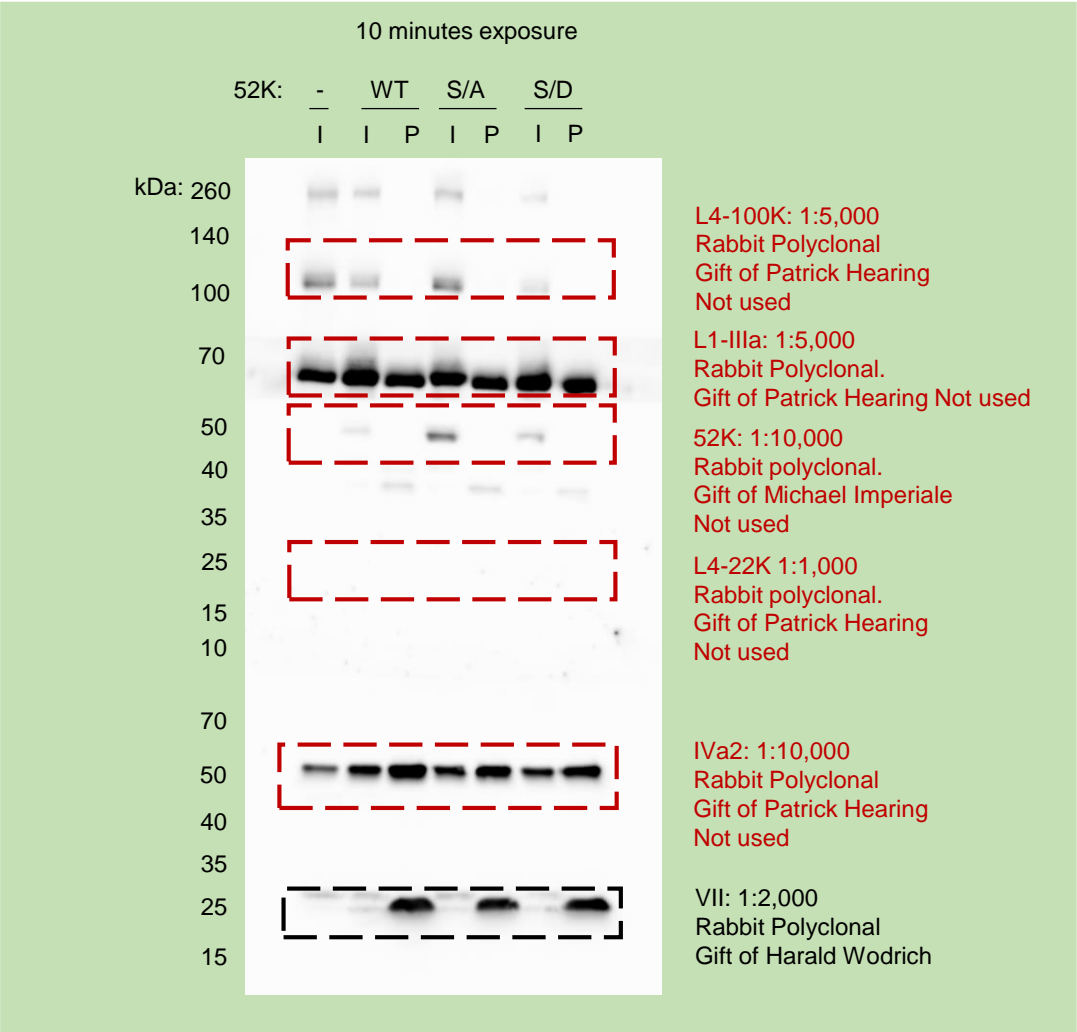

Figure 5: Panel H continued

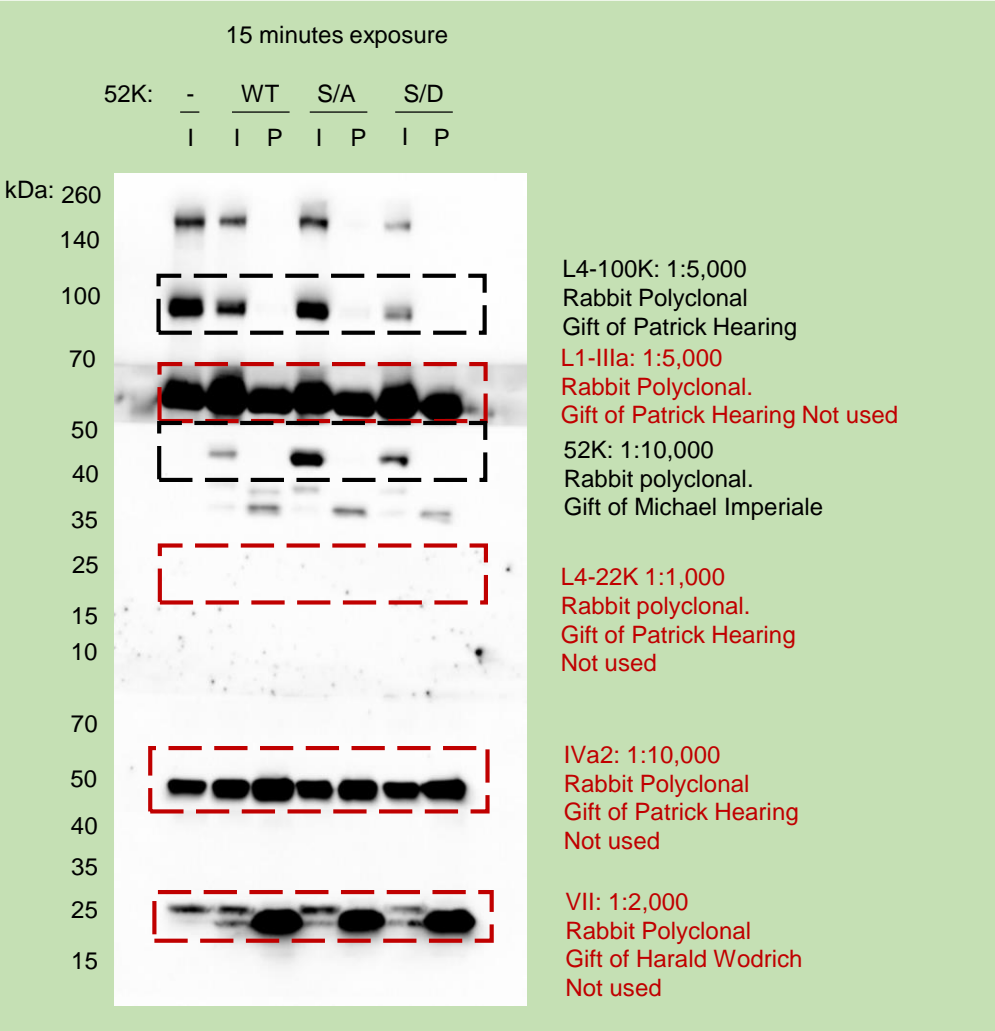

Supplement: Supplementary file 12 — Source Data Fig. 5 [file 44318_2023_21_MOESM12_ESM.zip › Figure 5/Figure 5J/Panel J_Analysis of Incomplete and Packaged Particles from Delta 52K AdV Rescue_Immunoblot.pdf]
