# Supplementary material for: Phosphorylation regulates viral biomolecular condensates to promote infectious progeny production
Source: EMBO J. 2024 Jan 2;43(2):6. doi: 10.1038/s44318-023-00021-0 (PMC10897327; doi:10.1038/s44318-023-00021-0)

Figure 3: Panel A

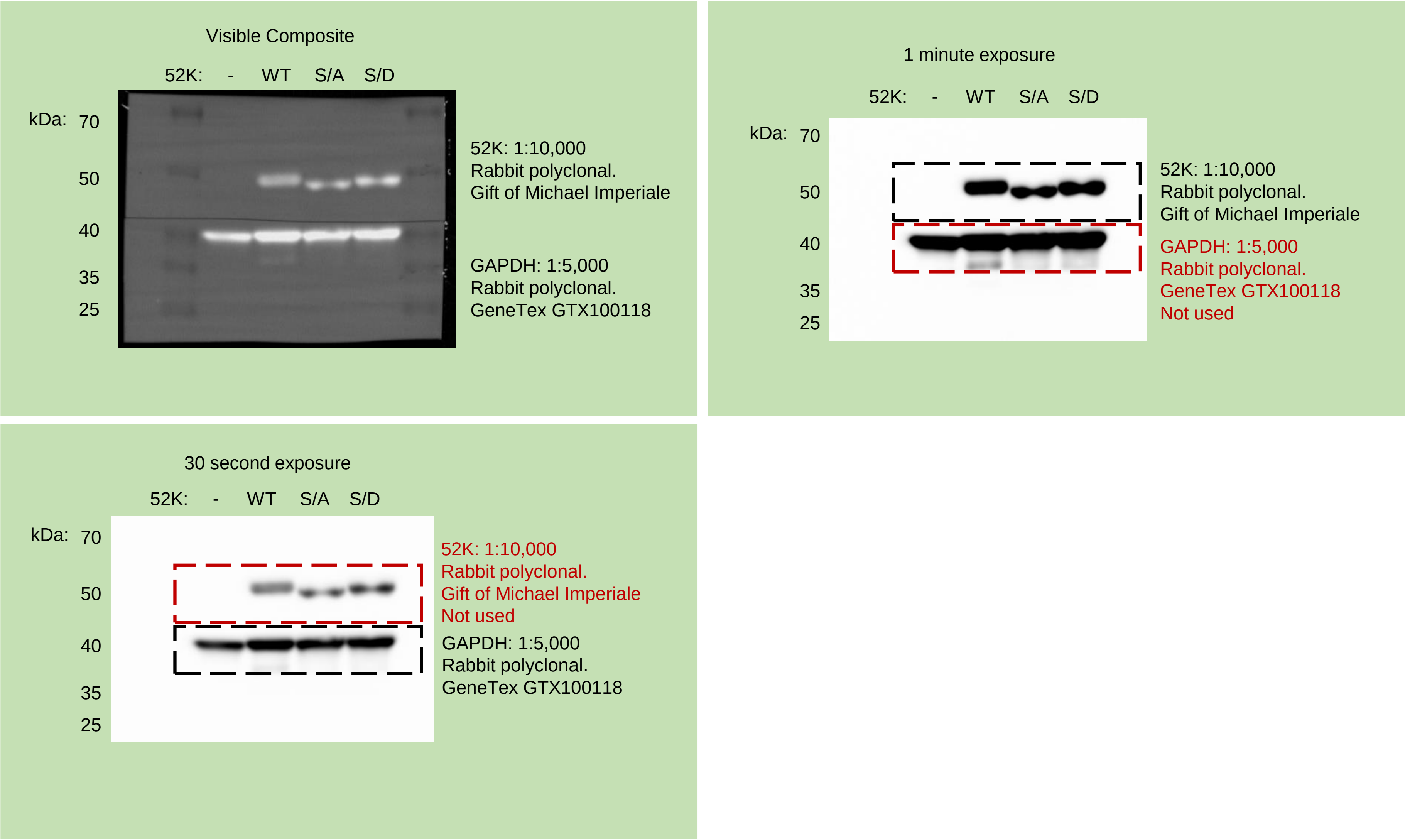

Supplement: Supplementary file 10 — Source Data Fig. 3 [file 44318_2023_21_MOESM10_ESM.zip › Figure 3/Figure 3A/Panel A_Expression of 52K in A549 52K Transgenic Cells Immunoblot.pdf]
