# Supplementary material for: Phosphorylation regulates viral biomolecular condensates to promote infectious progeny production
Source: EMBO J. 2024 Jan 2;43(2):6. doi: 10.1038/s44318-023-00021-0 (PMC10897327; doi:10.1038/s44318-023-00021-0)
Supplement: Supplementary file 13 — Expanded View Figures [file 44318_2023_21_MOESM13_ESM.pdf]

Expanded View Figures

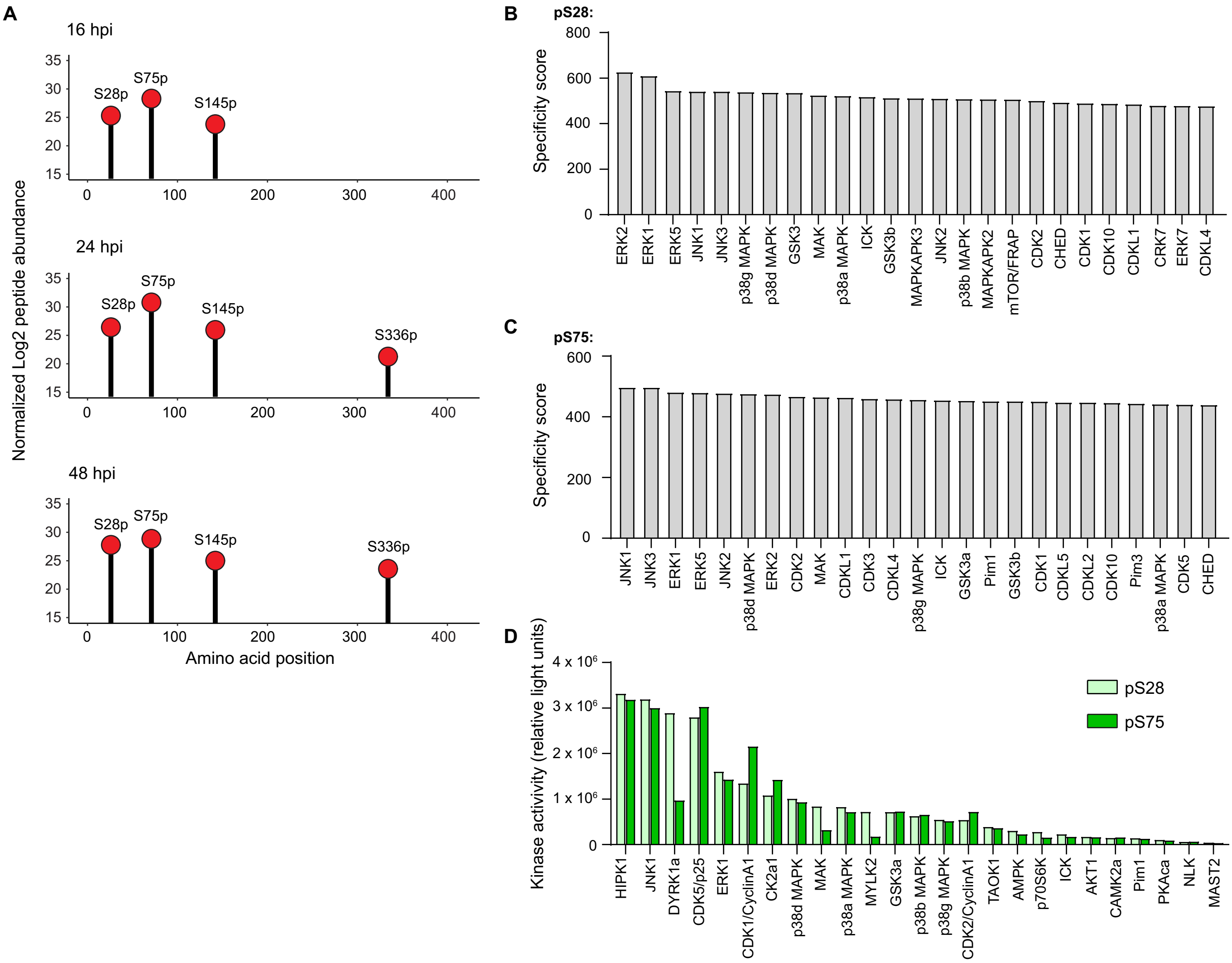

Figure EV1. Site specific phosphorylation of 52K.

(**A**) Analysis of posttranslational modification-mass spectrometry dataset showing site specificity of phosphorylation during adenovirus (AdV) infection of A549 cells at 16-, 24-, or 48-h post infection (hpi). Red lollipops denote phosphorylated serine residues S28, S75, S145, and S336 respectively. Modifications shown as log2 abundance of modified peptide relative to total peptide. (**B**) Bar graph showing the top 25 human kinases with the highest in silico catalytic site specificity scores matching the 52K protein S28 phospho-site. (**C**) Bar graph showing the top 25 human kinases with the highest in silico catalytic site specificity scores matching the 52K protein S75 phospho-site. (**D**) Bar graph showing the relative activity of 25 human kinases in vitro when provided with peptide substrates matching either the 52K S28 or S75 phospho-sites. Kinase activity was measured as relative light units.

 © The Author(s)

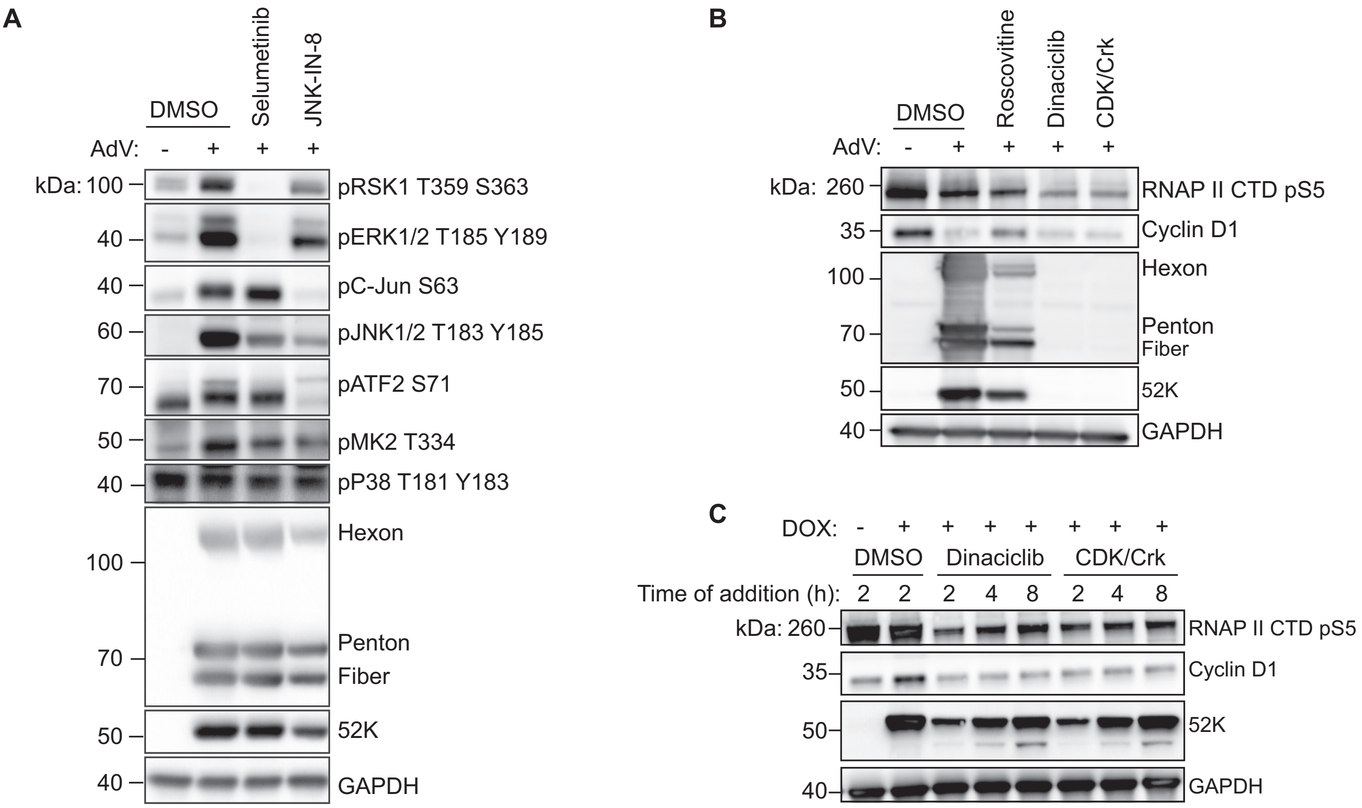

Figure EV2. Inhibition of MAPK and CDK family kinases.

(**A**) Immunoblot showing 52K and viral late proteins (Hexon, Penton, Fiber) from adenovirus (AdV) infected A549 cells treated with MAP Kinase inhibitors. DMSO control or 10 μM of kinase inhibitor (Selumetinib = MEK1/2, JNK-IN-8 = JNK1/2) was added at 2 h post infection (hpi). Infection was allowed to progress in the presence of inhibitor or DMSO for 24 h before being harvested for immunoblot. Downstream-target controls (pRSK1, pERK1/2 = Selumetinib. pC-Jun, pJNK1/2, pATF2 = JNK-IN-8, pMK2) confirm efficacy of the inhibitors. Phosphorylated P38 (pP38) serves as a kinase specificity control. GAPDH is included as a loading control. Representative of $n = 3$ independent replicates. (**B**) Immunoblot showing 52K and viral late proteins (Hexon, Penton, Fiber) from AdV-infected A549 cells treated with Cyclin-Dependent Kinase inhibitors. DMSO control or 10 μM of kinase inhibitor (Roscovitine = CDK2/5, Dinaciclib = CDK4/6, CDK/crk = CDK1/2/4/5/7/9) was added at 2 hpi. Infection was allowed to progress in the presence of inhibitor or DMSO for 24 h before being harvested for immunoblot. Downstream-target controls (RNAP II CTD pS5 and Cyclin D1) confirm efficacy of the inhibitors. GAPDH is included as a loading control. Representative of $n = 3$ independent replicates. (**C**) Immunoblot showing 52K from transgenic A549 cells treated with Cyclin-Dependent Kinase inhibitors. Expression of WT 52K was induced by addition of doxycycline and DMSO control or 10 μM of kinase inhibitor (Dinaciclib = CDK4/6, CDK/crk = CDK1/2/4/5/7/9) was added at the indicated time post induction. Protein accumulation was allowed to progress in the presence of inhibitor or DMSO for 24 h before being harvested for immunoblot. Downstream-target controls (pRNAP II CTD pS5 and Cyclin D1) confirm efficacy of the inhibitors. GAPDH is included as a loading control. Representative of $n = 3$ independent replicates.

© The Author(s)

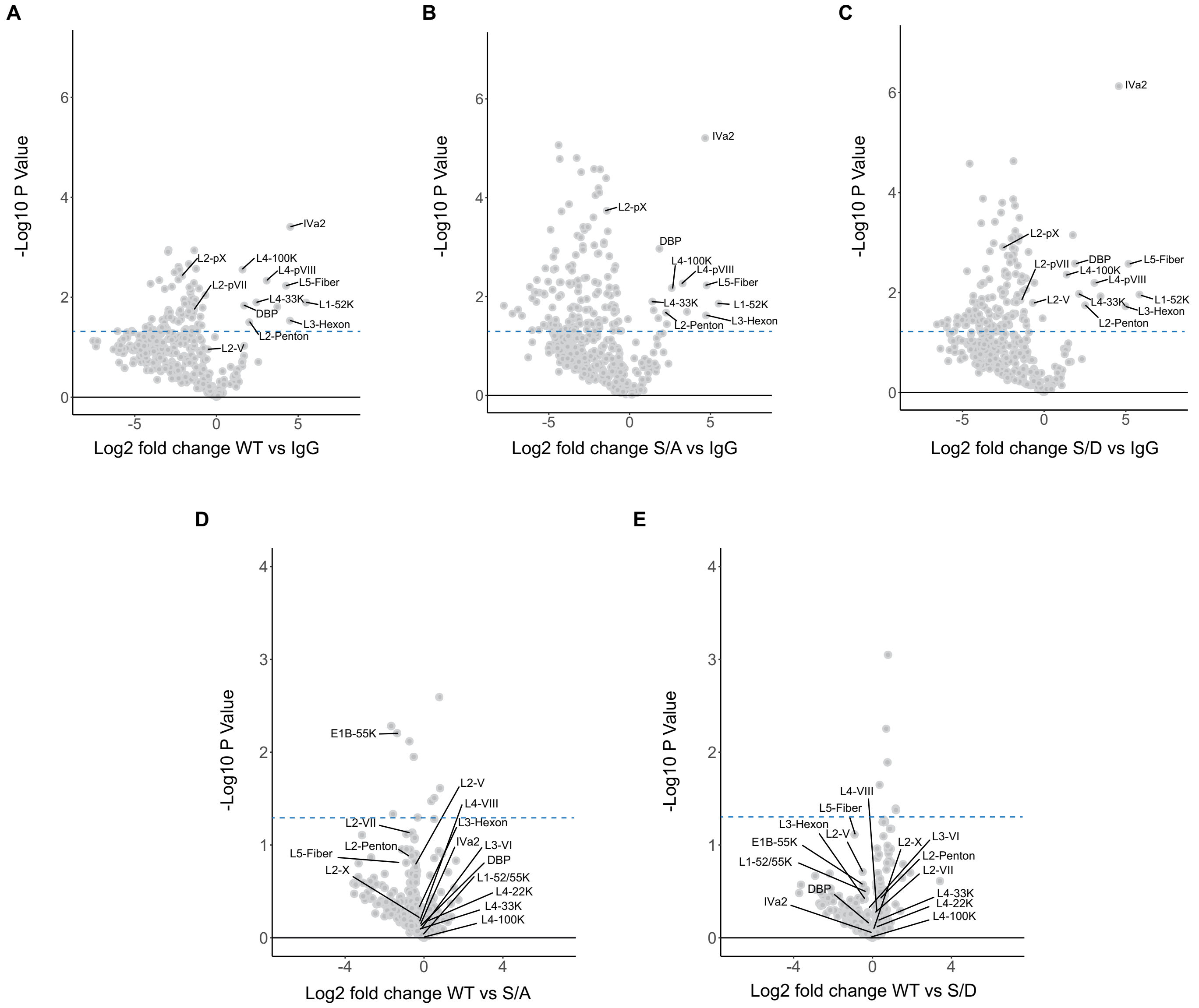

Figure EV3. Phosphorylation of 52K does not influence interactions with viral proteins.

(**A**) Volcano plot showing mean relative abundance of proteins co-precipitated from Δ52K mutant adenovirus (AdV) infected HEK-293 cell lysates with wild-type 52K (WT) versus IgG control. Viral proteins detected are labeled. $n = 4$ independent replicates. (**B**) Volcano plot showing mean relative abundance of proteins co-precipitated from Δ52K mutant AdV-infected HEK-293 cell lysates with S28/75 A (S/A) mutant versus IgG control. Viral proteins detected are labeled. $n = 4$ independent replicates. (**C**) Volcano plot showing mean relative abundance of proteins co-precipitated from Δ52K mutant AdV-infected HEK-293 cell lysates with S28/75D (S/D) mutant versus IgG control. Viral proteins detected are labeled. $n = 4$ independent replicates. (**D**) Volcano plot showing mean relative abundance of proteins co-precipitated from Δ52K mutant AdV-infected HEK-293 cell lysates with wild-type 52K (WT) compared to the phosphorylation-deficient S/A mutant. Viral proteins detected are labeled. $n = 4$ independent replicates. (**E**) Volcano plot showing mean relative abundance of proteins co-precipitated from Δ52K mutant AdV-infected HEK-293 cell lysates with wild-type 52K (WT) compared to the phosphorylation-mimetic S/D mutant. Viral proteins detected are labeled. $n = 4$ independent replicates. Data information: Data are presented as the average of four independent replicates. For all sample comparisons, two-sided Student's T tests were used to determine significant changes with a P value < 0.05. The dotted blue line shows the statistical cutoff of $P < 0.05$ (**A–E**).

 © The Author(s)

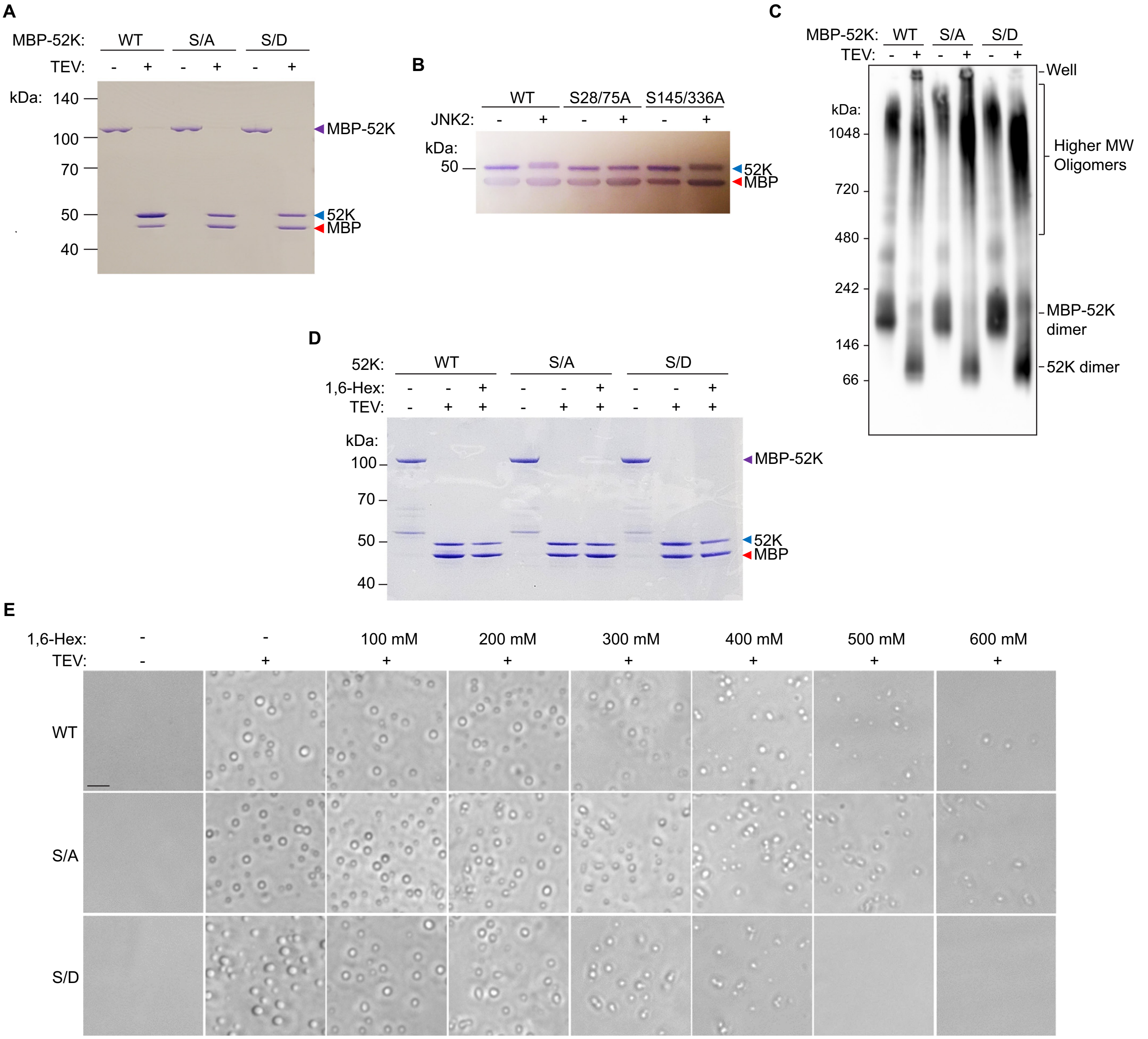

Figure EV4. Impact of phosphorylation on the 52K protein in vitro.

(**A**) Coomassie Brilliant Blue stained SDS-PAGE showing maltose binding protein (MBP) tagged wild-type (WT), S28/75 A (S/A), or S28/75D (S/D) 52K fusion proteins with (+) or without (−) Tobacco Etch Virus (TEV) protease cleavage for 1 h at room temperature. Representative of $n = 3$ independent replicates. (**B**) Coomassie Brilliant Blue stained SDS-PAGE showing electrophoretic mobility of wild-type 52K (WT), S28/75 A or S145/336 A following treatment with (+) or without (−) JNK2 kinase. The MBP tag was removed by TEV protease cleavage. Representative of $n = 3$ independent replicates. (**C**) Immunoblot for 52K following native-PAGE of MBP-tagged wild type (WT), S28/75 A, or S28/75D 52K fusion proteins with (+) or without (−) TEV protease cleavage for 1 h at room temperature. Representative of $n = 3$ independent replicates. (**D**) Coomassie Brilliant Blue stained SDS-PAGE showing maltose binding protein (MBP) tagged wild type (WT), S28/75 A (S/A), or S28/75D (S/D) 52K fusion proteins with (+) or without (−) Tobacco Etch Virus (TEV) protease cleavage for 1 h at room temperature in the absence (−) or presence of 600 mM 1,6-hexanediol (+). Representative of $n = 3$ independent replicates. (**E**) Wide-field microscope images showing condensates formed by phase separation of 5 μM wild-type 52K (WT), S28/75 A (S/A), or S28/75D (S/D) in vitro in the absence (−) or presence of 1,6-hexanediol (+) at the indicated concentrations (100 mM–600 mM). Phase separation was enabled by removal of the MBP tag by addition of TEV protease (+). A no TEV control (−) is included. Scale bars = 10 μm. Representative of $n = 3$ independent replicates.

© The Author(s)

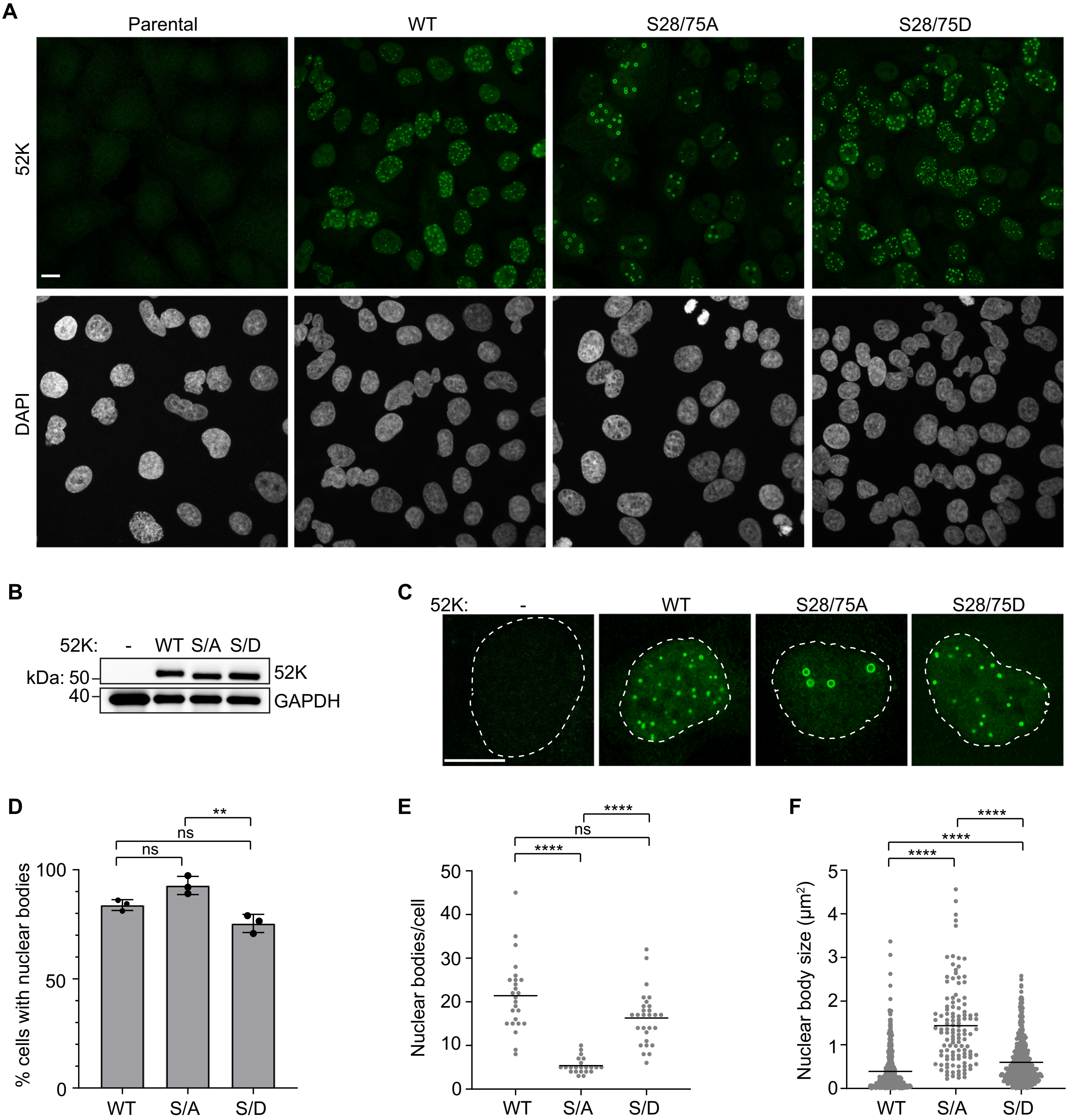

Figure EV5. Phosphorylation of the 52K protein IDR alters nuclear body morphology in HEK-293 cells.

(**A**) Immunofluorescence–confocal microscope images showing cell to cell variability in size and number of nuclear bodies in parental or transgenic A549 cell lines expressing wild-type 52K (WT), or phosphorylation mutants S28/75 A, or S28/75D at 24 h after induction of expression by addition of doxycycline. Scale bar = 5 µm. Representative of $n = 3$ independent replicates. (**B–F**) HEK-293 cells expressing wild-type 52K (WT), phosphorylation mutant S28/75A (S/A) or S28/75D (S/D), or mock-transfected control (−) as indicated. (**B**) Immunoblot showing the levels of ectopically expressed 52K. GAPDH is shown as a loading control. Representative of $n = 3$ independent replicates. (**C**) Immunofluorescence–confocal microscope images showing the morphology of 52K nuclear bodies. Nuclei outlined (dashed white line). Scale bar = 5 µm. Representative of $n = 3$ independent replicates. (**D**) Percentage of cells with nuclear bodies. $n = 3$ independent replicates each consisting of 3 fields of view analyzed. (**E**) Number of nuclear bodies per cell. $n = 3$ independent replicates pooled. A total of 25 (WT), 22 (S/A), or 28 (S/D) cells were analyzed. (**F**) Nuclear body size. $n = 3$ independent replicates pooled. Data information: Data are presented as mean ± standard deviation (**D–F**). One-way ANOVA with Tukey's pairwise comparison tests (**D**) or Kruskal–Wallis ANOVA with Dunn's pairwise comparison tests (**E**, **F**). NS $P > 0.05$, $^{**}P < 0.01$, $^{****}P < 0.0001$.

 © The Author(s)

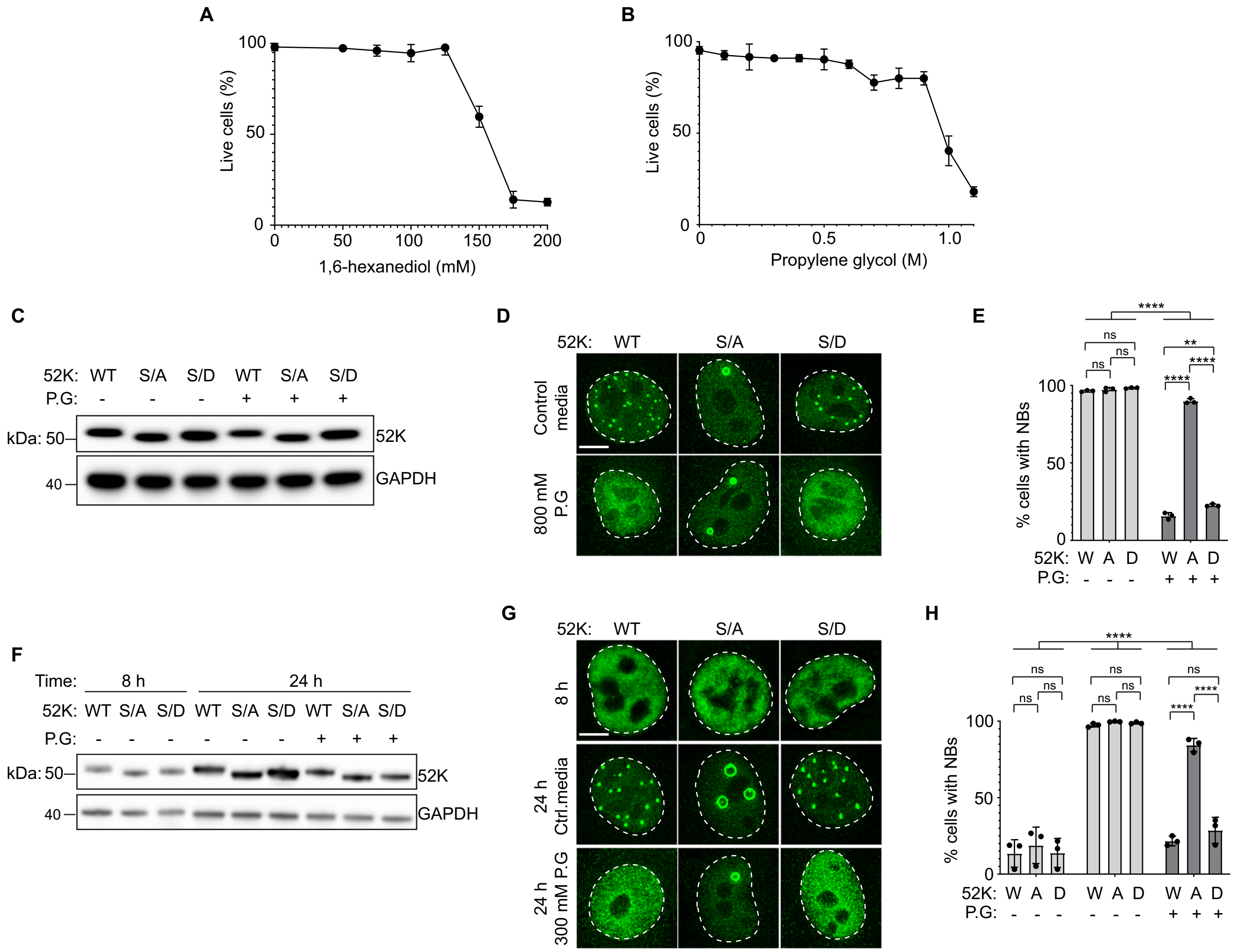

Figure EV6. Phosphorylation of the 52K IDR impacts sensitivity of nuclear bodies to diols.

(**A**) Percentage of live cells after incubation of A549 cells in the presence of the indicated concentration of 1,6-hexanediol for 24 h as determined by trypan blue staining. $n = 3$ independent replicates. (**B**) Percentage of live cells after incubation of A549 cells in the presence of the indicated concentration of propylene glycol for 24 h as determined by trypan blue staining. $n = 3$ independent replicates. (**C–E**) Expression of the 52K protein and nuclear body formation in transgenic A549 cells. Expression of wild-type 52K (WT), S28/75 A (S/A), or S28/75D (S/D) was induced by addition of doxycycline for 10 h before addition of 800 mM propylene glycol (P.G) for 10 min prior to analysis. $n = 3$ independent replicates. (**C**) Immunoblot showing levels of 52K. GAPDH is shown as a loading control. (**D**) Representative confocal microscope images showing the localization of 52K. Nuclei are outlined (dashed white line). Scale bar = 5 μm. (**E**) Percentage of cells with nuclear bodies. Each independent replicate consisted of 3 fields of view. (**F–H**) Transgenic A549 cells. Expression of wild-type 52K (WT), S28/75 A (S/A), or S28/75D (S/D) was induced by addition of doxycycline for 8 h before incubation with control media or media containing 300 mM propylene glycol (P.G) for an additional 16 h (24 h post induction). $n = 3$ independent replicates. (**F**) Immunoblot showing levels of 52K. High exposure panel shows the lower levels of expression at 8 h. GAPDH is shown as a loading control. (**G**) Representative images showing the localization of 52K. Nuclei are outlined (dashed white line). Scale bar = 5 μm. (**H**) Percentage of cells with nuclear bodies. Each independent replicate consisted of 3 fields of view. Data information: Data are presented as mean ± standard deviation (**A**, **B**, **E**, **H**). Two-way ANOVA with Tukey's multiple comparison's tests (**E**, **H**). NS $P > 0.05$, $^{**}P < 0.01$, $^{****}P < 0.0001$.

© The Author(s)
